# On the mechanism of imine elimination from Fischer tungsten carbene complexes

**DOI:** 10.3762/bjoc.12.125

**Published:** 2016-06-27

**Authors:** Philipp Veit, Christoph Förster, Katja Heinze

**Affiliations:** 1 Institute of Inorganic and Analytical Chemistry, Johannes Gutenberg-University, Duesbergweg 10-14, 55128 Mainz, Germany

**Keywords:** carbene complexes, ferrocene, imine, mechanism, tungsten

## Abstract

(Aminoferrocenyl)(ferrocenyl)carbene(pentacarbonyl)tungsten(0) (CO)_5_W=C(NHFc)Fc (**W(CO)_5_(*E*-2)**) is synthesized by nucleophilic substitution of the ethoxy group of (CO)_5_W=C(OEt)Fc (**M(CO)_5_(1^Et^)**) by ferrocenyl amide Fc-NH^–^ (Fc = ferrocenyl). **W(CO)_5_(*E*-2)** thermally and photochemically eliminates bulky *E*-1,2-diferrocenylimine (***E*-3**) via a formal 1,2-H shift from the N to the carbene C atom. Kinetic and mechanistic studies to the formation of imine ***E*-3** are performed by NMR, IR and UV–vis spectroscopy and liquid injection field desorption ionization (LIFDI) mass spectrometry as well as by trapping experiments for low-coordinate tungsten complexes with triphenylphosphane. **W(CO)_5_(*E*-2)** decays thermally in a first-order rate-law with a Gibbs free energy of activation of Δ*G*^‡^_298K_ = 112 kJ mol^−1^. Three proposed mechanistic pathways are taken into account and supported by detailed (time-dependent) densitiy functional theory [(TD)-DFT] calculations. The preferred pathway is initiated by an irreversible CO dissociation, followed by an oxidative addition/pseudorotation/reductive elimination pathway with short-lived, elusive seven-coordinate hydrido tungsten(II) intermediates ***cis*(N,H)-W(CO)_4_(H)(*Z*-15)** and ***cis*(C,H)-W(CO)_4_(H)(*Z*-15)**.

## Introduction

**Scheme 1:**
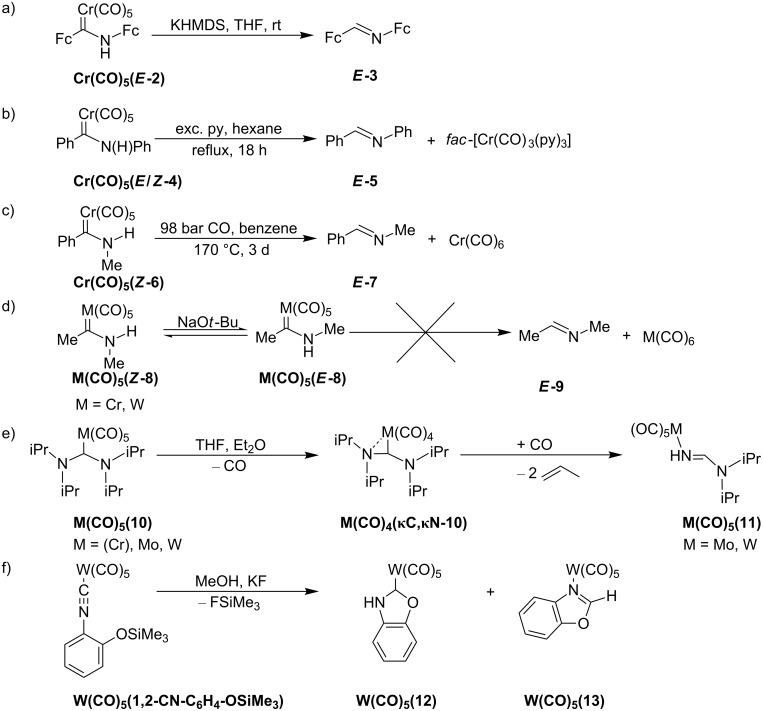
Imine formation and isomerization reactions from NH carbene complexes **Cr(CO)_5_(*E*-2)** (a) [27], **Cr(CO)_5_(*E*/*Z*-4)** (b) [44], **Cr(CO)_5_(*Z*-6)** (c) [42], **M(CO)_5_(*Z*-8)** (d) [45–46], **M(CO)_5_(10)** (e) [50–52] and during Si–O cleavage in the isonitrile complex **W(CO)_5_(1,2-CN-C_6_H_4_-OSiMe_3_)** (f) [53].

Since the first example of a Fischer carbene complex (CO)_5_W=C(OMe)Me [1] in 1964, these compounds have evolved into a huge substance class with versatile applications as chemical multitalents in organic synthesis [2–5] as well as in light-driven organic reactions [6–8]. Carbene complexes of pentacarbonyl metal fragments (M = Cr, Mo, W) have further proven to be effective carbene transfer agents to late transition metals in transmetalation reactions [9–15]. The manifold synthetic access routes to carbene complexes even allows the assembly of multicarbene and multimetal carbene complexes [16–17]. First representatives of multimetal carbene complexes **M(CO)_5_(1^R^)** bear α-ferrocenyl alkoxy carbenes :C(OR)Fc (**1^R^**, M = Cr, Mo, W; R = Me, Et; Fc = ferrocenyl) [18–22]. Nucleophilic substitution of the alkoxy substituent OR by amines gives access to α-ferrocenylamino Fischer carbene complexes [18,20–21,23–27], according to the classical Fischer route [28–31]. In contrast to conventional aromatic substituents, the Fc unit in **M(CO)_5_(1^R^)** is characterized by its redox activity and its large cylindrical steric bulk [32–33]. The electrochemical behaviour of ferrocenyl carbene complexes has been extensively investigated [25–27,34–39]. A second ferrocenyl unit can be incorporated by employing aminoferrocene (Fc-NH_2_) [40–41] in a nucleophilic substitution reaction [27]. The trimetallic complex **Cr(CO)_5_(*E*-2)** with the (aminoferrocenyl)ferrocenylcarbene ligand ***E*-2** is readily synthesized from **Cr(CO)_5_(1^Et^)** by nucleophilic substitution of the ethoxy group with in situ generated ferrocenyl amide Fc-NH^−^. Unlike the facile synthesis of the diphenyl derivative **Cr(CO)_5_(*E*-4)** from **Cr(CO)_5_(1^Et^)** and aniline [30,42], the preparation of the diferrocenyl derivative **Cr(CO)_5_(*E*-2)** from bulky Fc-NH_2_ [40–41] requires the presence of a base to increase the nucleophilicity of Fc-NH_2_ by deprotonation. In the presence of base, **Cr(CO)_5_(*E*-2)** decomposes readily in solution at room temperature releasing *E*-1,2-diferrocenylimine [43] ***E*-3** (Scheme 1a) [27]. Base assisted imine formation of NH carbene complexes typically occurs under rather harsh conditions. Thermal treatment of the significantly less encumbered complex **Cr(CO)_5_(*E*/*Z*-4)** for 18 h in a 1:10 (v:v) pyridine (py)/hexane mixture yields imine ***E*-5** and *fac*-[Cr(CO)_3_(py)_3_] as side-product (Scheme 1b) [44]. Formation of the imine ***E*-7** and Cr(CO)_6_ from the carbene complex **Cr(CO)_5_(*Z*-6)** requires heating to 170 °C for 3 days under CO pressure (Scheme 1c) [42]. At room temperature and in the presence of base (KO*t*-Bu), **M(CO)_5_(*Z*-8)** (M = Cr, W) simply isomerize to a mixture of *E*/*Z* isomers **M(CO)_5_(*E*-8)**/**M(CO)_5_(*Z*-8)** without ligand loss or formation of imine ***E*-9** (Scheme 1d) [45–46]. It appears that the bulky diferrocenylcarbene ***E*-2** facilitates formation of imine ***E*-3**. Mechanistically, a base-assisted 1,2-H shift can be conceived either at the coordinated carbene or at the free carbene [47–48] after ligand exchange at chromium (by py or CO) for **Cr(CO)_5_(*E*/*Z*-4)** and **Cr(CO)_5_(*Z*-6)**. Both pathways are compatible with the formation of the metal-containing products *fac*-[Cr(CO)_3_(py)_3_] and Cr(CO)_6_ by dissociation of the imines ***E*-5** or ***E*-7** or by dissociation of the carbenes ***E*-4** or ***E*-6**, respectively (Scheme 1b,c) [42,44].

The related pentacarbonyl complexes of bis[di(isopropyl)amino]carbene **10** [49] **M(CO)_5_(10)** (M = Cr, Mo, W) readily decarbonylate at room temperature to give the tetracarbonyl complexes **M(CO)_4_(κC,κN-10)** with a side-on coordinated carbene ligand (Scheme 1e) [50–52]. Under CO atmosphere, the molybdenum and tungsten complexes **M(CO)_4/5_(10)**, (M = Mo, W) eliminate two equivalents of propene giving the imine complexes **M(CO)_5_(11)**. Formation of the imine complex tungsten(benzoxazole)(pentacarbonyl) **W(CO)_5_(13)** has been reported by Tamm and Hahn during the synthesis of the carbene complex tungsten(benzoxazolin-2-ylidene)(pentacarbonyl) **W(CO)_5_(12)** (Scheme 1f) [53].

In principle, the formation of imines from NH carbene complexes can occur by three conceivable fundamental pathways. The first pathway starts with the dissociation of the carbene followed by a 1,2-H shift at the free carbene (elimination–migration). The second one operates via a hydrogen atom shift at the coordinated carbene followed by dissociation of the resulting imine (migration–elimination). A third conceivable pathway could start with CO loss, followed by H atom migration. To the best of our knowledge, the mechanism of the imine formation from NH carbene complexes is not yet established.

In the absence of a base, the bulky diferrocenylcarbene complex **Cr(CO)_5_(*E*-2)** is stable even in refluxing toluene and hence, a simple migration–elimination or elimination–migration reaction is not anticipated in this case. We report here the heavier tungsten analogue **W(CO)_5_(*E*-2)** which is thermally reactive and smoothly forms the imine ***E*-3** without the need of prior deprotonation. This apparently simpler reaction allows the investigation of the mechanism of imine formation from NH carbene complexes.

Herein, the synthesis and characterization of **W(CO)_5_(*E*-2)** followed by detailed mechanistic studies regarding the formation of imine ***E*-3** are presented including mass spectrometric, NMR, IR and UV–vis spectroscopic kinetic studies in combination with (TD)-DFT methods.

## Results and Discussion

### Synthesis of **W(CO)_5_(*E*-2)**

**Scheme 2:**
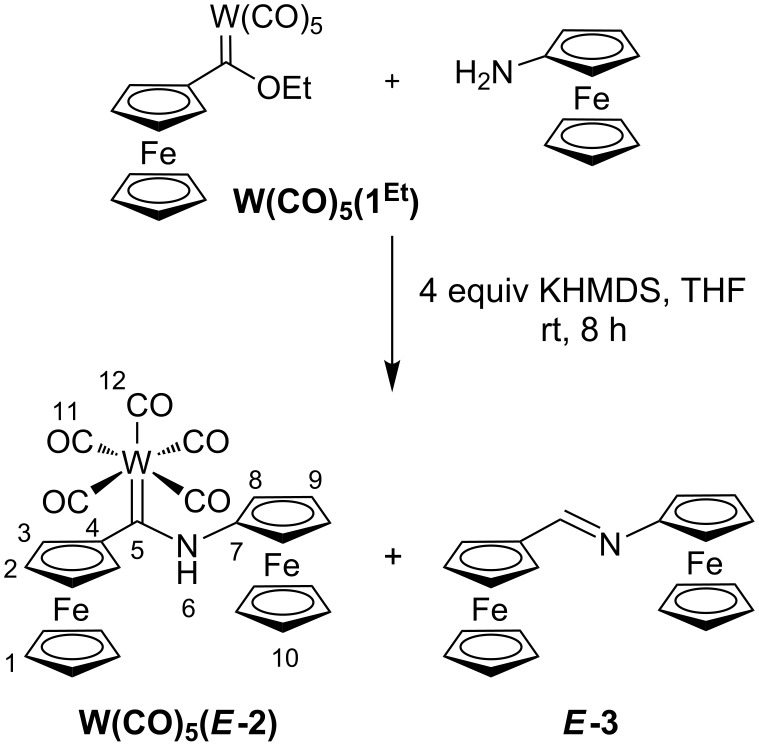
Synthesis of **W(CO)_5_(*E*-2)** from **W(CO)_5_(1^Et^)** [20–21] and aminoferrocene [40–41] with concomitant formation of *E*-1,2-diferrocenylimine ***E*-3** [43] as side-product and atom numbering of **W(CO)_5_(*E*-2)** for NMR assignments.

The diferrocenyl NH carbene complex **W(CO)_5_(*E*-2)** is obtained by treating **W(CO)_5_(1^Et^)** [20–21] with aminoferrocene (Fc-NH_2_) [40–41] in the presence of potassium hexamethyldisilazide (KHMDS) in tetrahydrofuran at room temperature (Scheme 2). In an analogous reactivity to **Cr(CO)_5_(*E*-2)** (Scheme 1a) [27], the formation of the imine ***E*-3** is observed as a side-reaction under the alkaline conditions. Due to this reactivity, **W(CO)_5_(*E*-2)** is obtained in only 28% yield as a deep-red crystalline compound after purification via column chromatography.

### Characterization of **W(CO)_5_(*E*-2)**

The composition and purity of **W(CO)_5_(*E*-2)** is ascertained by mass spectrometry, showing the expected molecular ion peak at *m*/*z* = 721 with appropriate isotopic pattern, and elemental analysis (Experimental section and Supporting Information File 1). At increasing temperature in the FD mass spectrometer, peaks at *m*/*z* = 397 appear which can be assigned to a molecular ion of the composition C_21_H_19_NFe_2_. A tiny peak cluster at *m*/*z* = 693, assignable to the loss of CO from **W(CO)_5_(*E*-2)**, and peaks at higher *m*/*z* ratios, assignable to tungsten clusters, are also observed when traces of oxygen/water were present. Using ^1^H and ^13^C NMR spectroscopy as well as 2D NMR (^1^H,^1^H COSY, ^1^H,^1^H NOESY, ^13^C,^1^H HSQC, ^13^C,^1^H HMBC techniques), all ^1^H and ^13^C NMR resonances of **W(CO)_5_(*E*-2)** are assigned based on coupling patterns and NOE contacts (Experimental section and Supporting Information File 1). Only the resonances of the unsubstituted C_5_H_5_ ligands (H^1^, H^10^ and C^1^, C^10^) could not be discriminated. The proton resonances are found in a similar region as for other (pentacarbonyl)tungsten complexes **W(CO)_5_(*E*-14^R^)** with the α-ferrocenyl NH carbene ligand :C(NHR)Fc ***E*-14^R^** (R = Me, Et, *n*-Pr [23], *n*-Bu [25], *n*-Pent [21]). Due to additional ring-current effects and non-classical NH···Fe hydrogen bonding [54–59] of the NH-Fc moiety, the resonance for the amine proton NH^6^ (δ = 10.50 ppm in CD_2_Cl_2_) is shifted to lower field as compared to that of alkylamine substituted NH carbene complexes **M(CO)_5_(*E*-14^R^)** (δ = 9.00–9.11 ppm in CDCl_3_) [21,23]. The NH···Fe interaction is also supported by the low-energy NH stretching vibration of **W(CO)_5_(*E*-2)** at 3240 cm^−1^ in CD_2_Cl_2_, which matches to that of **Cr(CO)_5_(*E*-2)** (3233 cm^–1^) [27] (Experimental section and Supporting Information File 1). A weak absorption band at 3439 cm^−1^ is tentatively assigned to some **W(CO)_5_(*Z*-2)** isomer lacking the NH···Fe interaction. In the solid state (KBr) the NH stretching vibration appears at 3335 cm^−1^ (Experimental section and Supporting Information File 1). The C–N–H bending vibration is observed as a single sharp relatively strong band at 1508 cm^−1^. These IR data reveal that the main isomer in solution as well in the solid state is the *E* isomer in accordance with the IR data of **W(CO)_5_(*E*/*Z*-8)** [46]. The carbonyl region of IR spectra of **W(CO)_5_(*E*-2)** are in accordance with those of **Cr(CO)_5_(*E*-2)** [27] and related amino(ferrocenyl)carbene(pentacarbonyl)tungsten complexes **W(CO)_5_(*E*-14^R^)** (R = Me, Et, *n*-Pr [23], *n*-Bu [25], *n*-Pent [21]). The UV–vis spectrum of **W(CO)_5_(*E*-2)** (Supporting Information File 1) is similar to that of **Cr(CO)_5_(*E*-2)** [27] and to those of carbene(pentacarbonyl)metal complexes (Cr, W) [60–61].

Thermolysis of **W(CO)_5_(*E*-2)** in refluxing toluene gives imine ***E*-3** [43] after ca. 24 h in almost quantitative yield, as monitored by ^1^H NMR spectroscopy. Accordingly, **W(CO)_5_(*E*-2)** is a suitable candidate to investigate the imine formation from NH carbene complexes in a simple one-component system under relatively mild conditions and, importantly, in the absence of a base.

### DFT studies on the formation of imine ***E*-3** from **W(CO)_5_(*E*-2)**

Three conceivable reaction pathways for the formation of imine ***E*-3** have been considered. For each pathway, density functional theory (DFT) calculations on the B3LYP/LANL2DZ (IEF-PCM toluene) level of theory have been performed to localize minimum structures and energies of the intermediates which are connected by transition states. The Gibbs free energies are reported at 298 K.

**Scheme 3:**
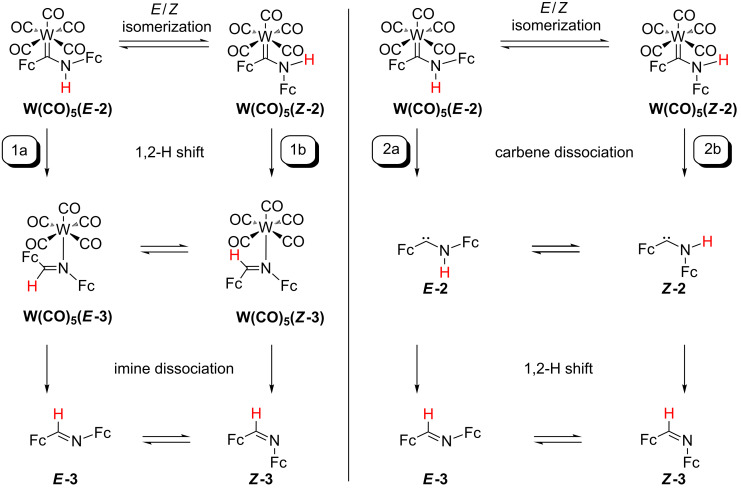
Reaction pathways 1a/1b (migration–elimination) and 2a/2b (elimination–migration) for the formation of imine ***E*-3** from **W(CO)_5_(*E*-2)**.

The first pathway comprises the migration–elimination mechanism involving a 1,2-H shift at the coordinated carbene ligand ***E*-2** or ***Z*-2** in **W(CO)_5_(*E*-2)** or **W(CO)_5_(*Z*-2)** followed by dissociation of the respective imine ***E*-3** (pathway 1a, Scheme 3) or ***Z*-3** (pathway 1b, Scheme 3), respectively. In the latter case, ***Z*-3** isomerizes to the thermodynamically preferred isomer ***E*-3**. The second pathway (carbene elimination–migration) starts with the elimination of the carbenes ***E*-2** or ***Z*-2** followed by an 1,2-H shift to give the imines ***E*-3** (pathway 2a, Scheme 3) or ***Z*-3** (pathway 2b, Scheme 3). In the latter case, a ***Z*-3** → ***E*-3** isomerization follows.

**Scheme 4:**
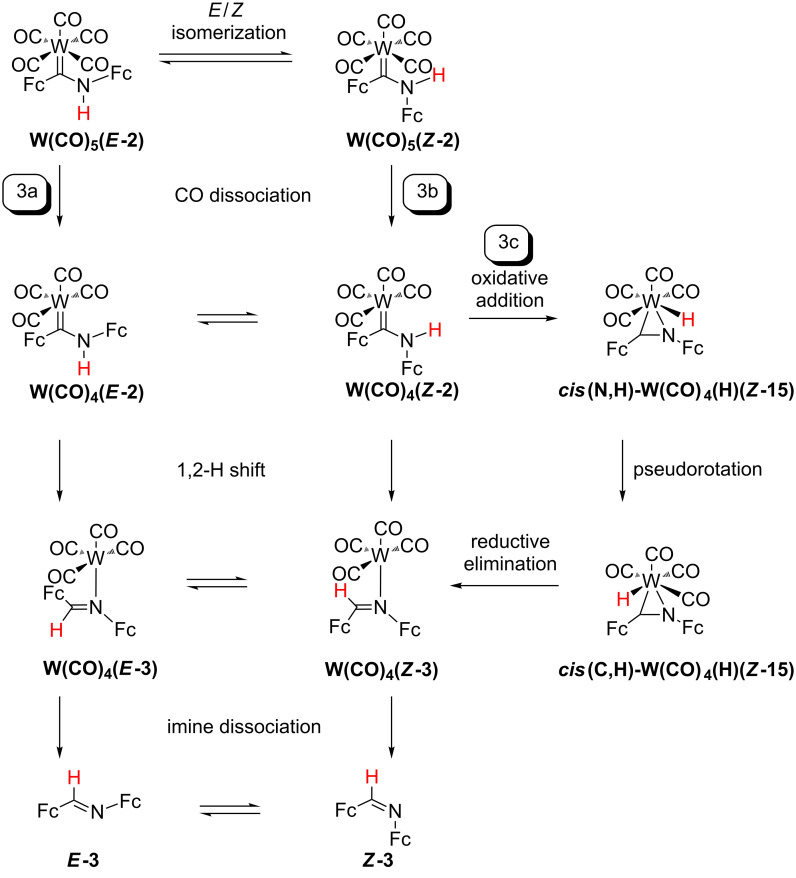
Reaction pathways 3a/3b/3c (CO dissociation) for the formation of imine ***E*-3** from **W(CO)_5_(*E*-2)**.

The initial step of the third pathway is a CO dissociation yielding the tetracarbonyl complexes **W(CO)_4_(*E*-2)** or **W(CO)_4_(*Z*-2)**. This elimination is followed by a hydrogen atom shift at the coordinated carbene ligands ***E*-2** or ***Z*-2** (pathway 3a and 3b, Scheme 4) giving **W(CO)_4_(*E*-3)** or **W(CO)_4_(*Z*-3)**, respectively. Furthermore, the free coordination site in **W(CO)_4_(*E*-2)** or **W(CO)_4_(*Z*-2)** offers an oxidative addition/pseudorotation/reductive elimination pathway via the hydrido tungsten(II) complexes **W(CO)_4_(H)(*Z*-15)** with the formally anionic ligand [Fc-C=N-Fc]^–^ (**15^–^**) as further alternative (pathway 3c, Scheme 4).

The calculated Gibbs free energies for **W(CO)_5_(*E*-2)** and **W(CO)_5_(*Z*-2)** are basically identical (Supporting Information File 1, Figure S16, Scheme 3). The calculated barrier for the *E*/*Z* isomerization **W(CO)_5_(*E*-2)** → **W(CO)_5_(*Z*-2)** amounts to Δ*G*^‡^ = 108 kJ mol^−1^. This barrier is significantly higher than that reported for (methoxy)(methyl)carbene(pentacarbonyl)chromium(0) Cr(CO)_5_(C(OMe)Me) (52 kJ mol^−1^ (experimental) and ca. 61 kJ mol^−1^ (theoretical)) due to the larger steric bulk of the (aminoferrocenyl)ferrocenylcarbene **2**, the higher π-donating character of the amino substituent vs the alkoxy substituent thus increasing the C(carbene)–X double bond character (X = N, O) [62–64] and the loss of some attractive NH**^…^**Fe interaction (H**^…^**Fe(Fc-C) = 2.98 Å) in **W(CO)_5_(*E*-2)** [27,54–59]. The Gibbs free energy of activation for the 1,2-H shift in **W(CO)_5_(*Z*-2)** to give the imine complex **W(CO)_5_(*Z*-3)** amounts to Δ*G*^‡^ = 333 kJ mol^−1^ which is prohibitively large. For **W(CO)_5_(*E*-2)** → **W(CO)_5_(*E*-3)**, this barrier is somewhat smaller (Δ*G*^‡^ = 284 kJ mol^−1^), yet this 1,2-H shift initially only leads to a van-der-Waals adduct of the imine ***E*-3** [**W(CO)_5_^…^*E*-3]**. Hence, this hydrogen atom shift is coupled with a W–C(carbene) bond dissociation.

The turn over frequency (TOF) of catalytic cycles can be estimated from the energies of the TOF-determining transition state (TDTS) and the TOF-determining intermediate (TDI) [65]. The given energy difference between TDTS and TDI is the maximum energy span between a given intermediate and all following transition states of the cycle and can be understood as the overall Gibbs free energy of activation of the whole catalytic cycle [65]. This procedure can be translated to competing reaction paths. For pathways 1a and 1b (Scheme 3), the rate-determining intermediate (RDI) is **W(CO)_5_(*Z*-2)** and the rate-determining transition states (RDTS’s) are TS(**W(CO)_5_(*E*-2)** → **W(CO)_5_^…^*E*-3**) (pathway 1a) and TS(**W(CO)_5_(*Z*-2)** → **W(CO)_5_(*Z*-3)** (pathway 1b) giving the overall Gibbs free energies of activation Δ*G*^‡^*_total_* = 287 kJ mol^−1^ and Δ*G*^‡^*_total_* = 333 kJ mol^−1^, respectively. The lower energy pathway 1a is associated with the dissociation of the carbene ligand (Supporting Information File 1, Figure S16). Hence, the initial dissociation of the carbenes ***E*-2** and ***Z*-2** is considered in pathways 2a and 2b (Scheme 3).

Dissociation of the carbenes ***E*-2**/***Z*-2** from **W(CO)_5_(*E*-2)**/**W(CO)_5_(*Z*-2)** is calculated endergonic (Δ*G* = 141 kJ mol^−1^ and Δ*G* = 167 kJ mol^−1^, respectively, Scheme 3). Transition states for the carbene dissociation could not be identified. Hence, this initial dissociative step is probably not the one with the lowest energy. Nonetheless, the 1,2-H shift in the free carbenes has been calculated as well (Scheme 3).

The carbene ***E*-2** is 23 kJ mol^−1^ more stable than the ***Z*-2** isomer (Supporting Information File 1, Figure S17). The interconversion between these isomers ***E*-2** → ***Z*-2** (Δ*G*^‡^ = 130 kJ mol^−1^) proceeds via a bending vibration of the Cp–C(carbene)–N moiety. This reaction coordinate is fully analogous to the proposed mechanism of the *E*/*Z* isomerization of imines [66–67]. During the 1,2-H-shift of ***E*-2** to ***E*-3**, the migrating hydrogen atom interacts with the empty p_π_-type orbital of the carbene carbon atom (Δ*G*^‡^ = 250 kJ mol^−1^), which is in accordance with the established mechanism of 1,2-migration reactions of carbenes [47–48,68–71]. Interestingly, the 1,2-H-shift of ***Z*-2** (***Z*-2** → ***Z*-3**: Δ*G*^‡^ = 179 kJ mol^−1^) with a lower barrier occurs within the C–C(carbene)–N plane via a direct interaction of the n_σ_ orbital at the carbene carbon atom with the hydrogen 1s orbital*.* Because of the non-crossing rule, this path is symmetry forbidden for aromatic carbenes [47,72]. The calculated barrier of the *E*/*Z* isomerization ***Z*-3** → ***E*-3** (Δ*G*^‡^ = 52 kJ mol^−1^) is in good agreement with experimental data for other imines with similar steric bulk, e.g., (Fc)_2_C=NAr, leading to the global minimum ***E*-3** of pathways 2a and 2b (Scheme 3) [66–67]. ***E*-2** is the RDI for pathways 2a and 2b. The transition states TS(***E*-2** → ***E*-3**) (Δ*G*^‡^*_total_* = 250 kJ mol^−1^, pathway 2a) and TS(***Z*-2** → ***Z*-3**) (Δ*G*^‡^*_total_* = 202 kJ mol^−1^, pathway 2b) are the RDTS’s. The 1,2-H-shift of the free carbenes **2** preferably proceeds via pathway 2b (Supporting Information File 1, Figure S17).

**Figure 1:**
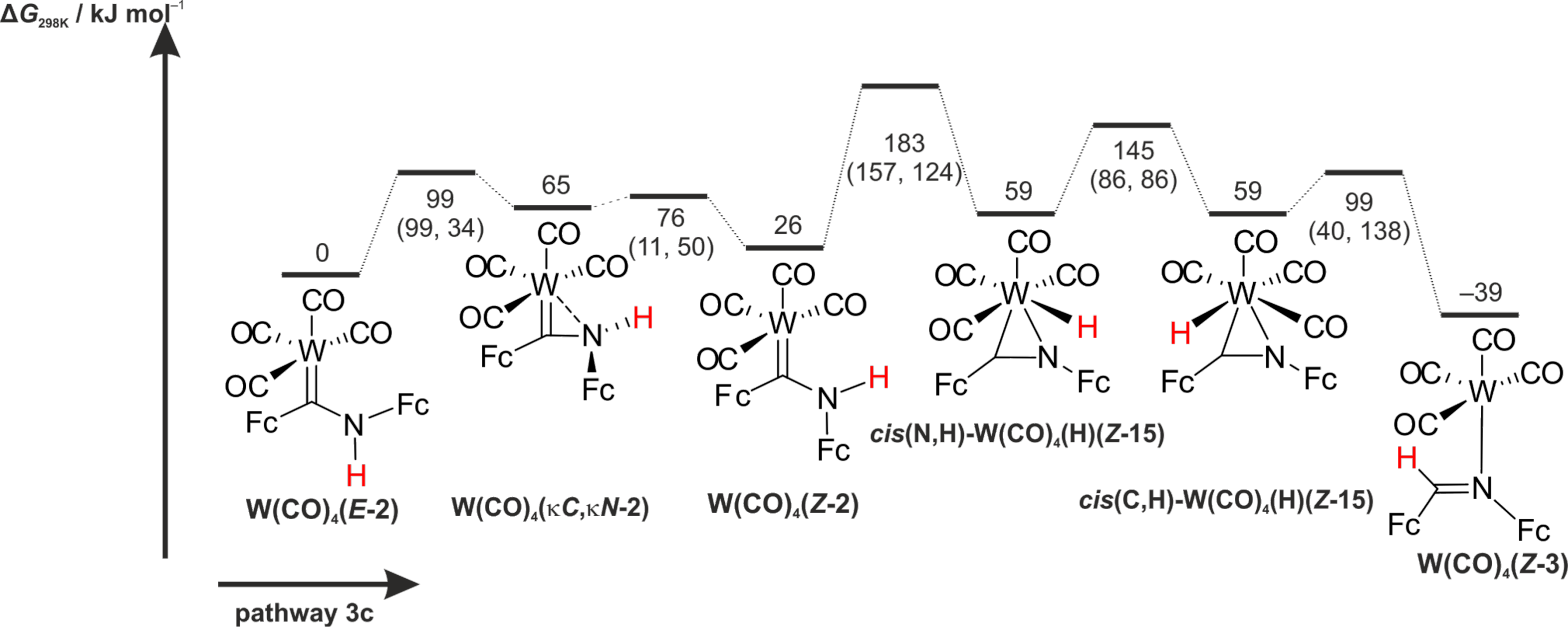
DFT calculated oxidative addition/pseudorotation/reductive elimination pathway 3c from **W(CO)_4_(*E*-2)** to **W(CO)_4_(*Z*-3)**.

**Figure 2:**
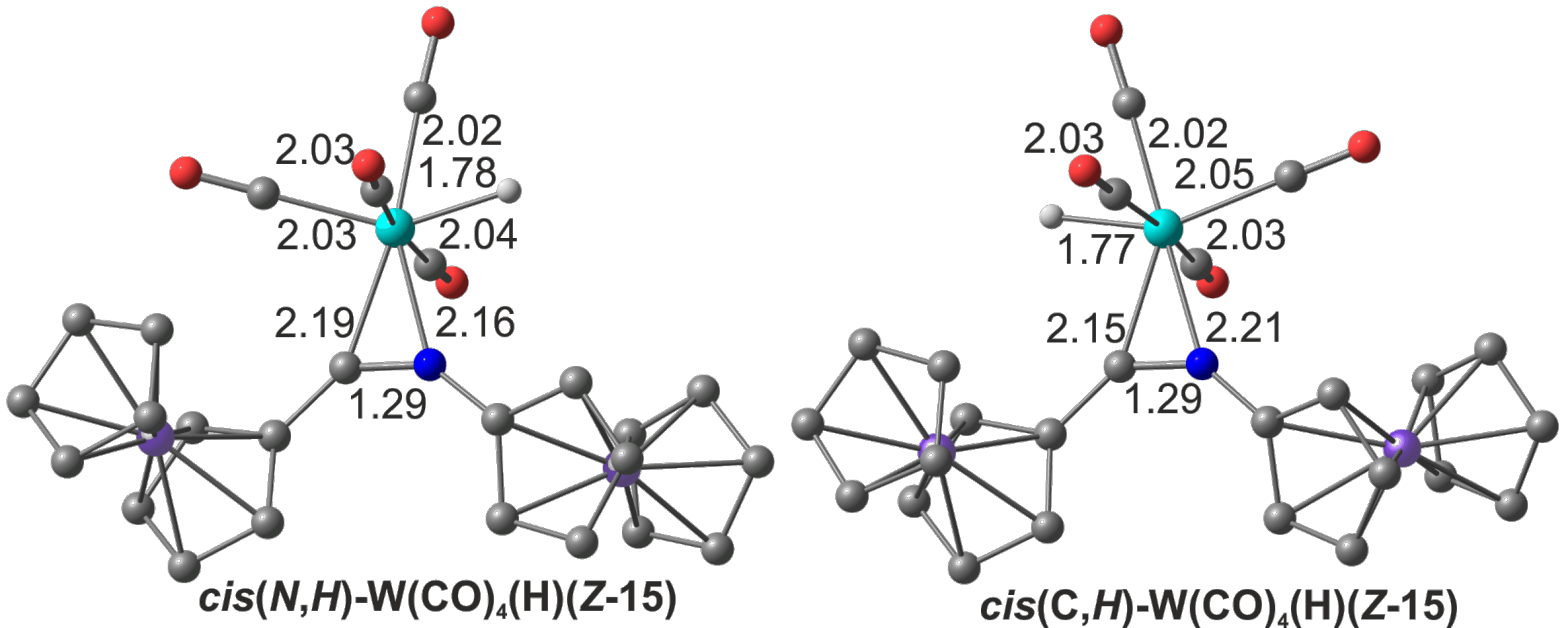
DFT calculated geometries of the two hydrido intermediates ***cis*(*N*,*H*)-W(CO)_4_(H)(*Z*-15)** and ***cis*(*C*,*H*)-W(CO)_4_(H)(*Z*-15)** and selected bond distances in Å.

Compared to the carbene dissociation, significantly smaller endergonicities are calculated for the dissociation of a carbonyl ligand (pathways 3a and 3b, Scheme 4) giving the tetracarbonyl complexes **W(CO)_4_(*E*-2)** and **W(CO)_4_(*Z*-2)** with Δ*G* = 86 kJ mol^−1^ and 115 kJ mol^−1^, respectively (Scheme 4). The **W(CO)_4_(*E*-2)** isomer is stabilized with respect to **W(CO)_4_(*Z*-2)** by 26 kJ mol^−1^. The *E*/*Z* isomerization of **W(CO)_4_(2)** proceeds via an intermediate **W(CO)_4_(κ*C*,κ*N*-2)** with a side-on coordination of the carbene ligand **2** exploiting the free coordination site at tungsten similar to **M(CO)_4_(κC,κN-10)** [50–52] (Scheme 1, Supporting Information File 1, Figure S18). The barrier for this *E*/*Z* carbene isomerization amounts to only Δ*G*^‡^ = 99 kJ mol^−1^. This represents the lowest barrier for the ***E*-2**/***Z*-2** isomerization calculated in the systems **W(CO)_5_(*E*-2/*Z*-2)**, **W(CO)_4_(*E*-2/*Z*-2)** and ***E*-2**/***Z*-2** (vide supra). However, the following 1,2-H shifts have high barriers of 294 and 248 kJ mol^−1^ for **W(CO)_4_(*E*-2)** → **W(CO)_4_(*E*-3)** and **W(CO)_4_(*Z*-2)** → **W(CO)_4_(*Z*-3)**, respectively. For the former reaction and similar to the 1,2-H shift in **W(CO)_5_(*E*-2)** (vide supra), the van-der-Waals adduct **[W(CO)_4_^…^*E*-3]** is the initial product implying the dissociation of the W–C(carbene) bond. The RDI for pathways 3a and 3b is **W(CO)_4_(*E*-2)**. The RDTS’s are TS(**W(CO)_4_(*E*-2)** → **[W(CO)_4_^…^*E*-3]**) (Δ*G*^‡^*_total_* = 294 kJ mol^−1^, pathway 3a) and TS(**W(CO)_4_(*Z*-2)** → **W(CO)_4_(*Z*-3)**) (Δ*G*^‡^*_total_* = 274 kJ mol^−1^, pathway 3b). Both pathways 3a and 3b are quite energy demanding and require even more energy than the ***E*-2** → ***E*-3** and ***Z*-2** → ***Z*-3** hydrogen atom migrations in the free carbenes (vide supra). The free coordination site at tungsten in **W(CO)_4_(*Z*-2)** provides a third sequence for the formation of imine ***E*-3** (Scheme 4, Figure 1). Oxidative addition of the NH bond to the unsaturated tungsten center (Δ*G*^‡^ = 157 kJ mol^−1^) gives the seven-coordinate hydrido tungsten(II) complex ***cis*(N,H)-W(CO)_4_(H)(*Z*-15)** with formally anionic [Fc-C=N-Fc]^−^ (**15^−^**) and hydrido ligands (Scheme 4, Figure 1 and Figure 2).

A similar oxidative addition has been proposed in the literature for the iron (aminophenyl)phenylcarbene complex [Cp(CO)(S(SiEt_3_))Fe(**4**)] leading to an intermediate hydrido species followed by the elimination of *E*-1,2-diphenylimine ***E*-5** [73]. Pseudorotation of ***cis*(N,H)-W(CO)_4_(H)(*Z*-15)** to the isoenergetic rotamer ***cis*(C,H)-W(CO)_4_(H)(*Z*-15)** (Δ*G*^‡^ = 86 kJ mol^−1^) enables a low-energy reductive elimination (Δ*G*^‡^ = 40 kJ mol^−1^) to give the imine complex **W(CO)_4_(*Z*-3)** (Figure 1).

The overall Gibbs free energy of activation amounts to only Δ*G*^‡^_t_*_otal_* = 183 kJ mol^−1^ with the RDI **W(CO)_4_(*E*-2)** and the RDTS TS(**W(CO)_4_(*Z*-2)** → ***cis*(*N*,*H*)-W(CO)_4_(H)(*Z*-15)**) (pathway 3c) for this preferred reaction sequence (Figure 1 and Supporting Information File 1, Figure S19).

All overall Gibbs free energies of activation for the discussed pathways 1a/1b and 3a/3b in the coordination sphere of the metal center are higher than for the carbene → imine isomerization in the metal-free systems ***E*-2** → ***E*-3** and ***Z*-2** → ***Z*-3** (pathways 2a/2b). This suggests that W(CO)_5_ or W(CO)_4_ coordination to ***E*-2** or ***Z*-2** kinetically stabilizes the carbene ligand. All pathways 1a/1b, 2a/2b and 3a/3b have large overall Gibbs free energies of activation with Δ*G*^‡^*_total_* > 200 kJ mol^−1^. The alternative pathway 3c via CO dissociation, oxidative addition, pseudorotation and reductive elimination features the lowest overall Gibbs free energy of activation of Δ*G*^‡^*_total_* = 183 kJ mol^−1^. Although activation barriers for CO and carbene ligand dissociation steps could not be determined by DFT calculations, the formation of tetracarbonyl complexes is very probable, while the carbene dissociation is less likely. The experimentally determined barrier for CO dissociation from tungsten hexacarbonyl amounts to 193 kJ mol^−1^ [74]. According to the calculations, the **W(CO)_4_(*Z*-2)** isomer is accessible from the thermodynamically preferred **W(CO)_4_(*E*-2)** isomer. The following oxidative addition pathway 3c from **W(CO)_4_(*Z*-2)** via the hydrido complexes ***cis*(*N*,*H*)-W(CO)_4_(H)(*Z*-15)** and ***cis*(*C*,*H*)-W(CO)_4_(H)(*Z*-15)** provides the lowest energy pathway for the formation of the imines ***Z*-3** and ***E*-3**. Important calculated bond distances in these key intermediates amount to W–C(carbene) = 2.19, 2.15 Å, W–N = 2.16, 2.21 Å, W–H = 1.78, 1.77 Å and C–N = 1.29, 1.29 Å for ***cis*(*N*,*H*)-W(CO)_4_(H)(*Z*-15)** and ***cis*(*C*,*H*)-W(CO)_4_(H)(*Z*-15)**, respectively (Figure 2). These hydrido intermediates act as hydrogen atom shuttle from the nitrogen to the carbon atom in NH carbene tetracarbonyl tungsten complexes. Oxidative additions of XY bonds to low-coordinate W(CO)*_n_* fragments is a common reactivity pattern for tungsten carbonyl complexes [75–80] and appears to be operative in the present case as well.

### Experimental studies on the formation of imine ***E*-3** from **W(CO)_5_(*E*-2)**

Heating of a toluene solution of **W(CO)_5_(*E*-2)** results in the formation of the imine ***E*-3** according to ^1^H NMR spectroscopy (monitored by the NH proton resonance of **W(CO)_5_(*E*-2)** at δ = 10.16 ppm and the CH proton resonance of ***E*-3** at δ = 8.33 ppm; Supporting Information File 1, Figures S20–S24). The appearance of a resonance at δ = 9.68 ppm is assigned to a trace amount of **W(CO)_5_(*Z*-2)**. A dark precipitate (possibly tungsten nanoparticles [81–82]) forms during the thermolysis. The half-lives at 60, 70, 80, 90 and 100 °C amount to 145.9, 39.4, 28.9, 16.2 and 12.2 h. The time traces fit to a first order kinetics as anticipated in the absence of a base. An Eyring–Polanyi plot gives an activation enthalpy of Δ*H*^‡^ = 54.5 ± 10.4 kJ mol^−1^ and an activation entropy of Δ*S*^‡^ = –193 ± 30 J mol^−1^ K^−1^ (Supporting Information File 1, Figure S25). These values give a Gibbs free energy of activation of Δ*G*^‡^_298K_ = 112 kJ mol^−1^.

The ^1^H NMR spectra during thermolysis provide no hint for a long-lived intermediate and the reaction cleanly proceeds from the starting material **W(CO)_5_(*E*-2)** to the product ***E*-3**. No hydride resonances have been detected up to δ = −30 ppm in the ^1^H NMR spectra. This suggests that subsequent reactions after ligand dissociation proceed faster and the irreversible ligand dissociation is the rate-determining step.

Attempts to intercept low-coordinate tungsten intermediates were conducted by thermolysis of **W(CO)_5_(*E*-2)** in the presence of triphenylphosphane. In case of aminocarbenes, experiments on the synthesis and decomposition of carbene(tetracarbonyl)(phosphane) complexes of chromium and tungsten revealed the exclusive formation of ***cis*-M(CO)_4_(PR_3_)(carbene)** (R = *n*-Bu, Ph) [83–85]. **M(CO)_5_(PR_3_)** and ***trans*-M(CO)_4_(PR_3_)_2_** (M = Cr, W) have been detected as side-products [83–84].

**Scheme 5:**
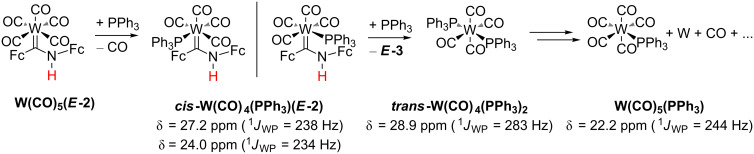
Proposed reaction sequence from **W(CO)_5_(*E*-2)** to **W(CO)_5_(PPh_3_)** in the presence of triphenylphosphane.

**W(CO)_5_(PPh_3_)** gives a ^31^P resonance at δ = 20.9 ppm (^1^*J*_WP_ = 243 Hz) in CDCl_3_ and ***trans*-W(CO)_4_(PR_3_)_2_** at δ = 27.4 ppm (^1^*J*_WP_ = 282 Hz) [86], while reported W(CO)_4_(PPh_3_)(carbene) complexes resonate in CD_2_Cl_2_ at δ = 24–25 ppm (^1^*J*_WP_ = 232–236 Hz) (*cis*) and at δ = 23 ppm (^1^*J*_WP_ = 209 Hz) (*trans*) and exhibit significantly smaller ^1^*J*_WP_ coupling constants [85]. A ^31^P NMR resonance of a toluene-*d*_8_ solution of **W(CO)_5_(*E*-2)** with one equivalent PPh_3_ heated to 100 °C for 1 h was observed at δ = 27.3 ppm with ^183^W satellites (^1^*J*_WP_ = 238 Hz) fitting to the carbene tetracarbonyl phosphane complex ***cis*-W(CO)_4_(PPh_3_)(*E*-2)** (23%) in addition to residual PPh_3_ (δ = −4.2 ppm, 73%) (Scheme 5, Supporting Information File 1, Figure S13). A less intense resonance (4%) at δ = 28.9 ppm (^1^*J*_WP_ = 283 Hz) is assigned to ***trans-*W(CO)_4_(PPh_3_)_2_** [86]. At later stages of the reaction, isomerization to another *cis* isomer of ***cis*-W(CO)_4_(PPh_3_)(*E*-2)** with δ = 24.0 ppm (^1^*J*_WP_ = 234 Hz), according to the ^1^*J*_WP_ coupling constant [85], probably occurs. After 4 h another ^31^P resonance appears at δ = 22.2 ppm (^1^*J*_WP_ = 244 Hz). Comparison of ^1^*J*_WP_ coupling constants confirms the presence of **W(CO)_5_(PPh_3_)** [86], with up to 67% spectroscopic yield after 25 h. Hence, the ^31^P NMR data suggest the following sequence as a main pathway: The sequence starts with initial loss of a CO ligand from **W(CO)_5_(*E*-2)**, followed by formation of ***cis*-W(CO)_4_(PPh_3_)(*E*-2)** complexes. Substitution of imine ***E*-3** by a second PPh_3_ ligand gives ***trans*-W(CO)_4_(PPh_3_)_2_**. Finally, **W(CO)_5_(PPh_3_)** is formed, presumably along with elemental tungsten (Scheme 5).

FD mass spectrometry confirms the most probable decomposition of **W(CO)_4_(PPh_3_)(*E*-2)** to ***trans*-W(CO)_4_(PPh_3_)_2_** (*m*/*z* = 820), **W(CO)_5_(PPh_3_)** (*m*/*z* = 586) and imine ***E*-3** (*m*/*z* = 397) (Supporting Information File 1, Figure S14). The carbonyl and CN stretching frequencies (2072, 2018, 1982, 1938, 1889, 1614 cm^−1^) and relative intensities of partially overlapping bands obtained by IR spectroscopy (Supporting Information File 1, Figure S15) fit to a mixture of **W(CO)_5_(PPh_3_)** (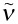 = 2072, 1982, 1938 cm^−1^) [87–88], **W(CO)_4_(PPh_3_)_2_** in *cis* (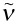 = 2018 cm^−1^) and *trans* (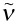 = 1938, 1889 cm^−1^) configuration [89–90] as well as imine ***E*-3** (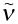 = 1614 cm^−1^). Hence, the initial thermally induced dissociation of a CO ligand is more favourable than carbene dissociation in agreement with the DFT calculations (vide supra).

Similarly, photochemical activation (400 nm LEDs) in toluene produces imine ***E*-3** in 31% yield after 120 h from **W(CO)_5_(*E*-2)** already at room temperature, while only 1% ***E*-3** is formed in the dark at room temperature. This observation additionally supports the hypothesis that the key initial step is the dissociation of CO from **W(CO)_5_(*E*-2)** to give **W(CO)_4_(*E*-2)** (Scheme 4).

Attempts to observe the tetracarbonyl intermediates in the absence of PPh_3_ by LIFDI mass spectrometry were unsuccessful. The mass spectra recorded at several time intervals during the heating procedure (reflux in toluene under strictly inert conditions) display the peak of the starting material at *m*/*z* = 721 and the peak of the imine product ***E*-3** at *m*/*z* = 397. The former peak decreases while the latter one increases during the heating process (Supporting Information File 1, Figure S26). No other intermediates appear in the FD mass spectra. Tentatively, the concomitantly formed tungsten species aggregate under these conditions and form the observed dark precipitate. This further supports the hypothesis that no intermediates accumulate during the reaction and that the rate-determining step is the CO ligand dissociation.

IR spectroscopic monitoring of a **W(CO)_5_(*E*-2)** solution in 1,2-dichloroethane under reflux (ca. 84 °C) shows that **W(CO)_5_(*E*-2)** simply decays to a carbonyl-free species (likely the dark precipitate) and no soluble CO-containing intermediates such as **W(CO)_4_(*E-*2)**, **W(CO)_4_(*Z*-2)** or hydrido carbonyl complexes are detected (Supporting Information File 1, Figure S27).

In full accordance with the above observations, UV–vis spectra recorded during the thermal treatment of **W(CO)_5_(*E*-2)** in toluene (100 °C) show the clean decay of the characteristic bands of **W(CO)_5_(*E*-2)** at 360 and 391 nm. The ferrocene based absorption band around 500 nm remains essentially constant indicating the stability of the Fc units. Isosbestic points are observed at 337 and 500 nm corroborating the clean conversion of **W(CO)_5_(*E*-2)** to ***E*-3** without long-lived soluble intermediates (Supporting Information File 1, Figure S28). The final UV–vis spectrum after 6 h closely resembles that of the calculated TD-DFT spectrum of imine ***E*-3** (Supporting Information File 1, Figure S29). All spectroscopic and analytical data suggest that the imine formation is faster than the CO dissociation.

## Conclusion

The thermally induced formation of *E*-1,2-diferrocenylimine ***E*-3** from the NH carbene pentacarbonyl tungsten complex **W(CO)_5_(*E*-2)** was investigated by density functional theory methods and mechanistic experimental studies (NMR, IR, UV–vis spectroscopy, FD mass spectrometry, kinetic studies, trapping of intermediates). All available data support the initial dissociation of a CO ligand to give the tetracarbonyl complex **W(CO)_4_(*E*-2)**. Isomerization to the **W(CO)_4_(*Z*-2)** isomer allows for an oxidative addition of the NH bond to give the seven-coordinate hydrido tungsten(II) complex ***cis*(N,H)-W(CO)_4_(H)(*Z*-15)**. After pseudorotation to the ***cis*(C,H)-W(CO)_4_(H)(*Z*-15)** rotamer, a reductive elimination yields the imine complex **W(CO)_4_(*Z*-3)**. All other conceivable pathways, namely 1,2-H shifts within the free carbene or within the carbonyl complexes **W(CO)_5_(*E*-2)** or **W(CO)_4_(*E*-2)**, are significantly more energy demanding. The possibility of a seven-coordinate tungsten(II) intermediate opens the oxidative addition/pseudorotation/reductive elimination pathway shuttling the hydrogen atom from the nitrogen atom via the W atom to the carbene carbon atom. This pathway is unfeasible for homologous chromium complexes and explains the resistance of **Cr(CO)_5_(*E*-2)** towards thermal ***E*-3** formation. A base-assisted pathway for imine formation is operative both for **Cr(CO)_5_(*E*-2)** and **W(CO)_5_(*E*-2)**, but the thermal imine formation is only feasible for **W(CO)_5_(*E*-2)**.

## Experimental

**General procedures:** All reactions were performed under argon atmosphere unless otherwise noted. A glovebox of the type UniLab/MBraun (Ar 4.8, O_2_ < 1 ppm, H_2_O < 1 ppm) was used for storage and weighing of sensitive compounds. All analytical samples that required the absence of oxygen were prepared in the same glovebox. Dichloromethane and 1,2-dichloroethane were dried with CaH_2_ and distilled prior to use. THF and toluene were distilled from potassium. All reagents were used as received from commercial suppliers (ABCR, Acros Organics, Alfa Aesar, Fischer Scientific, Fluka and Sigma-Aldrich). Deuterated solvents were purchased from euriso-top. (Ethoxy)(ferrocenyl)carbene(pentacarbonyl)tungsten(0) **W(CO)_5_(1^Et^)** [21] and Fc–NH_2_ [40–41] were prepared according to literature procedures.

NMR spectra were recorded on a Bruker Avance DRX 400 spectrometer at 400.31 MHz (^1^H), 100.07 MHz (^13^C{^1^H}) and 162.05 MHz (^31^P{^1^H}). All resonances are reported in ppm vs the solvent signal as internal standard [CD_2_Cl_2_ (^1^H: δ = 5.32 ppm; ^13^C: δ = 53.8 ppm), toluene-*d*_8_ (^1^H: δ = 2.08 ppm)] [91] and versus external H_3_PO_4_ (85%) (^31^P: δ = 0 ppm). IR spectra were recorded with a BioRad Excalibur FTS 3100 spectrometer as KBr disks or by using KBr cells in CH_2_Cl_2_ or in CD_2_Cl_2_. Electrochemical experiments were carried out on a BioLogic SP-50 voltammetric analyzer by using a platinum working electrode, a platinum wire as counter electrode and a 0.01 M Ag/AgNO_3_ electrode as reference electrode. The measurements were carried out at a scan rate of 100 mV s^−1^ for cyclic voltammetry experiments and at 50 mV s^−1^ for square wave voltammetry experiments in 0.1 M [*n*-Bu_4_N][B(C_6_F_5_)_4_] as supporting electrolyte in THF. Potentials are referenced against the decamethylferrocene/decamethylferrocenium couple (*E*_½_ = −525 ± 5 mV vs ferrocene/ferrocenium under our experimental conditions) and are given relative to the ferrocene/ferrocenium couple. UV–vis/NIR spectra were recorded on a Varian Cary 5000 spectrometer by using 1.0 cm cells (Hellma, suprasil). FD mass spectra were recorded on a Thermo Fisher DFS mass spectrometer with a LIFDI upgrade. Elemental analyses were performed by the microanalytical laboratory of the chemical institutes of the University of Mainz.

Density functional theory calculations were carried out with the Gaussian09/DFT series [92] of programs. The B3LYP [93] formulation of density functional theory was used employing the LANL2DZ [94–97] basis set. No symmetry constraints were imposed on the molecules. The presence of energy minima of the ground states was checked by analytical frequency calculations. The calculated transition states exhibit a single imaginary frequency and they were additionally verified by intrinsic reaction coordinate (IRC) calculations. Solvent modelling was done employing the integral equation formalism polarizable continuum model (IEFPCM, toluene). The approximate free energies at 298 K were obtained through thermochemical analysis of the frequency calculation, using the thermal correction to the Gibbs free energy as reported by Gaussian09.

**(Aminoferrocenyl)(ferrocenyl)carbene(pentacarbonyl)tungsten(0) (W(CO)_5_(*E*-2)):** 402 mg (2.0 mmol) of Fc-NH_2_ and 1132 mg (2.0 mmol) of **W(CO)_5_(1^Et^)** where dissolved in dry THF (40 mL). 1595 mg (8.0 mmol) of potassium hexamethyldisilazide (KHMDS) in dry THF (40 mL) were added within 5.5 h while stirring at room temperature. The reaction was monitored by TLC to check the reaction progress and to stop the reaction before extensive imine formation occurs. After 8 h, the solvent was removed under reduced pressure and an aqueous saturated NaHCO_3_ solution (100 mL) was added. The aqueous phase was extracted with dichloromethane (3 × 100 mL) and the combined organic phases were washed with aqueous saturated NaHCO_3_ solution (2 × 100 mL) and brine (2 × 100 mL). The organic phase was dried over MgSO_4_. After evaporation of the solvent under reduced pressure, a crude red product was obtained (1.04 mg). Purification by column chromatography (SiO_2_; 40 cm × 5.5 cm; petroleum ether (40/60):CH_2_Cl_2_ 1:1; *R*_f_(Fc–NH_2_) = 0.0, *R*_f_(***E*-3**) = 0.5, *R*_f_(**W(CO)_5_(*E*-2)**) = 0.8) yielded 403 mg (0.56 mmol, 28%) of deep red crystalline needles. ^1^H NMR (CD_2_Cl_2_): δ 10.50 (s, 1H, H6), 4.73 (pt, 2H, H8), 4.71 (pt, 2H, H3), 4.62 (pt, 2H, H2), 4.37 (s, 5H, H1/10), 4.33 (pt, 2H, H9), 4.32 (s, 5H, H1/10) ppm; ^13^C NMR (CD_2_Cl_2_) δ 259.6 (C5), 204.4 (C12), 199.3 (C11_, _^1^*J*_WC_ = 127 Hz), 99.7 (C7), 97.7 (C4), 72.1 (C2), 70.7 (C3), 70.6 (C1/10), 70.2 (C1/10), 69.1 (C8), 67.8 (C9) ppm; MS (FD) *m/z* (int.): 721.0 (100, [M]^+^); IR (KBr) 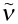: 3335 (m, NH), 3107 (s, CH), 2058 (vs, CO), 1977 (vs, CO), 1899 (br, CO), 1508 (s), 1350 (m), 1238 (m), 1057 (m), 822 (m), 600 (s), 579 (m), 480 (m) cm^−1^; IR (CH_2_Cl_2_) 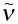: 2060 (vs, CO *A*_1_), 1975 (s, CO *B*_1_), 1921 (br, CO *E*, *A*_1_), 1503 (m) cm^−1^; IR (CD_2_Cl_2_) 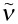: 3439 (w, NH(W(CO)_5_(*Z*-2))), 3240 (m, NH(W(CO)_5_(*E*-2))) cm^−1^; UV–vis (CH_2_Cl_2_) λ_max_ (ε): 290 sh (15370), 355 (11020), 387 (11680), 468 sh (2570 M^−1^ cm^−1^) nm; CV (THF, vs FcH/FcH^+^): *E*_1/2_ = −2.38 V (qrev.), *E*_p,ox_ = 0.26, 0.48 V, *E*_p,red_ = 0.17, –0.15, –0.76 V; Anal. calcd for C_26_H_19_Fe_2_NO_5_W (720.95): C, 43.31; H, 2.66; N, 1.94; found: C, 43.30; H, 2.69; N, 1.91.

## Supporting Information

File 1 Experimental spectra and DFT derived data.
