# Supplementary material for: On the mechanism of imine elimination from Fischer tungsten carbene complexes
Source: Beilstein J Org Chem. 2016 Jun 27;12:1322–33. doi: 10.3762/bjoc.12.125 (PMC4979739; doi:10.3762/bjoc.12.125)
Supplement: File 1 — Experimental spectra and DFT derived data. [file Beilstein_J_Org_Chem-12-1322-s001.pdf]

Supporting Information File

for

On the mechanism of imine elimination from Fischer tungsten carbene complexes

Philipp Veit[1], Christoph Förster[1*] and Katja Heinze[1]*

Address: [1]Institute of Inorganic and Analytical Chemistry, Johannes Gutenberg-University, Duesbergweg 10-14, 55128 Mainz, Germany

Email: Christoph Förster - cfoerster@uni-mainz.de; Katja Heinze - katja.heinze@uni.mainz.de

* Corresponding authors

In memoriam Prof. Dr. Peter Hofmann

Experimental spectra and DFT derived data

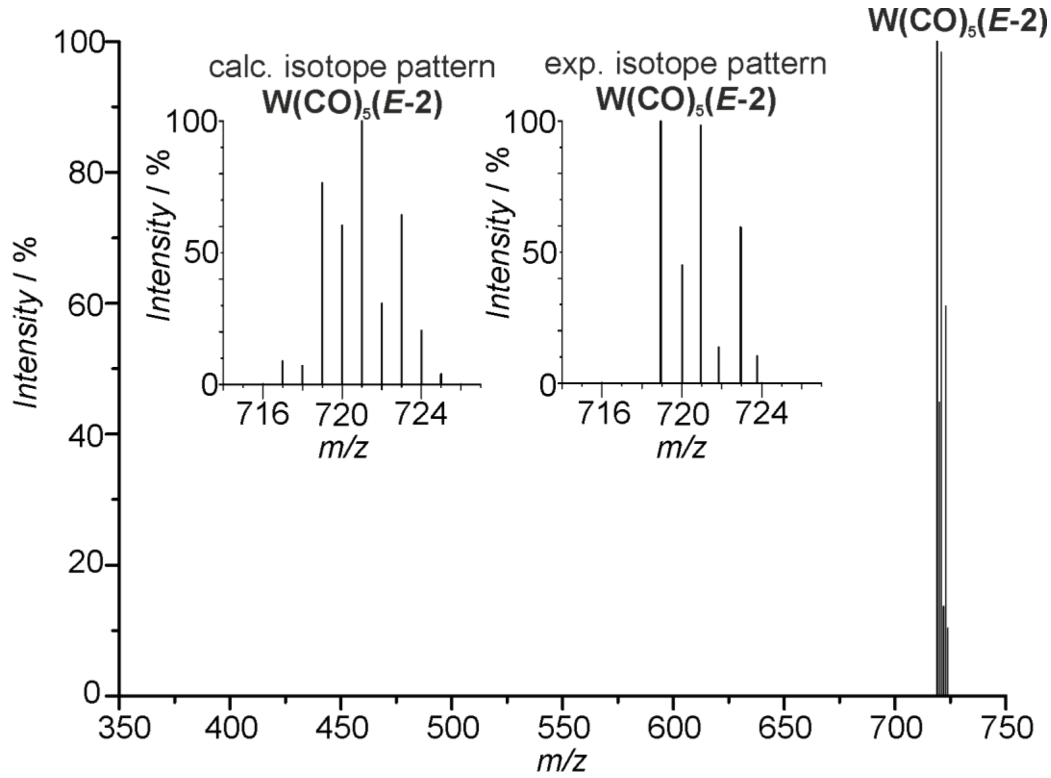

Figure S1: FD mass spectrum of **W(CO)$_5$(*E*-2)** in toluene.

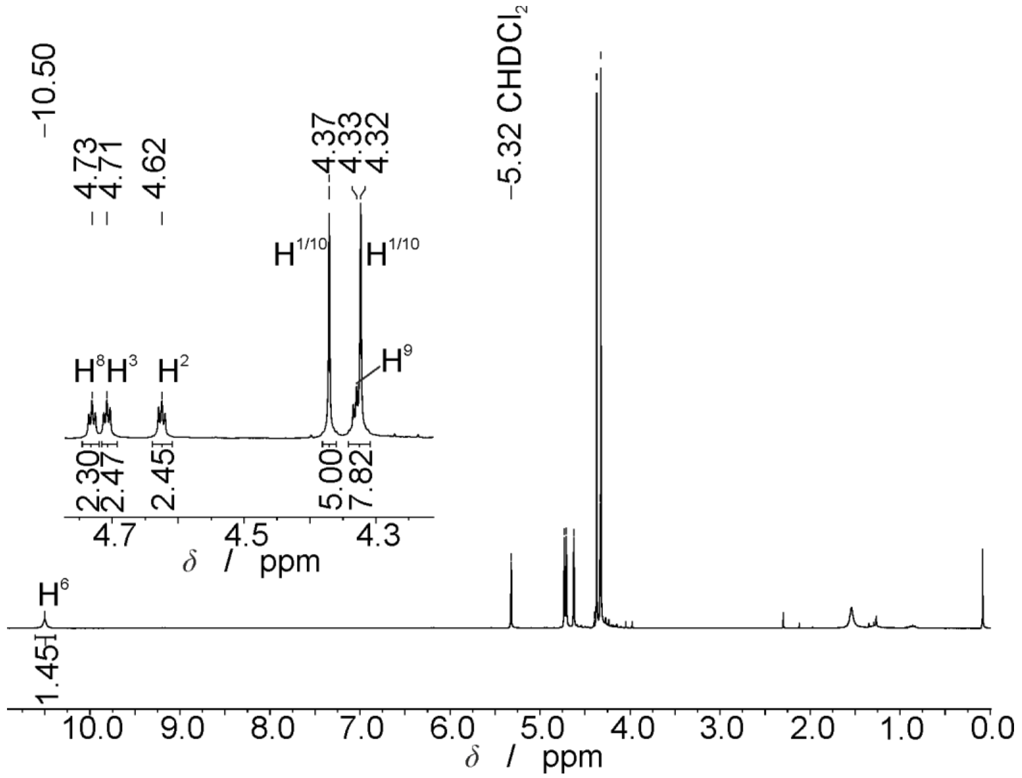

Figure S2: ^{1}H NMR spectrum of **W(CO)$_5$(*E*-2)** in CD_2Cl_2.

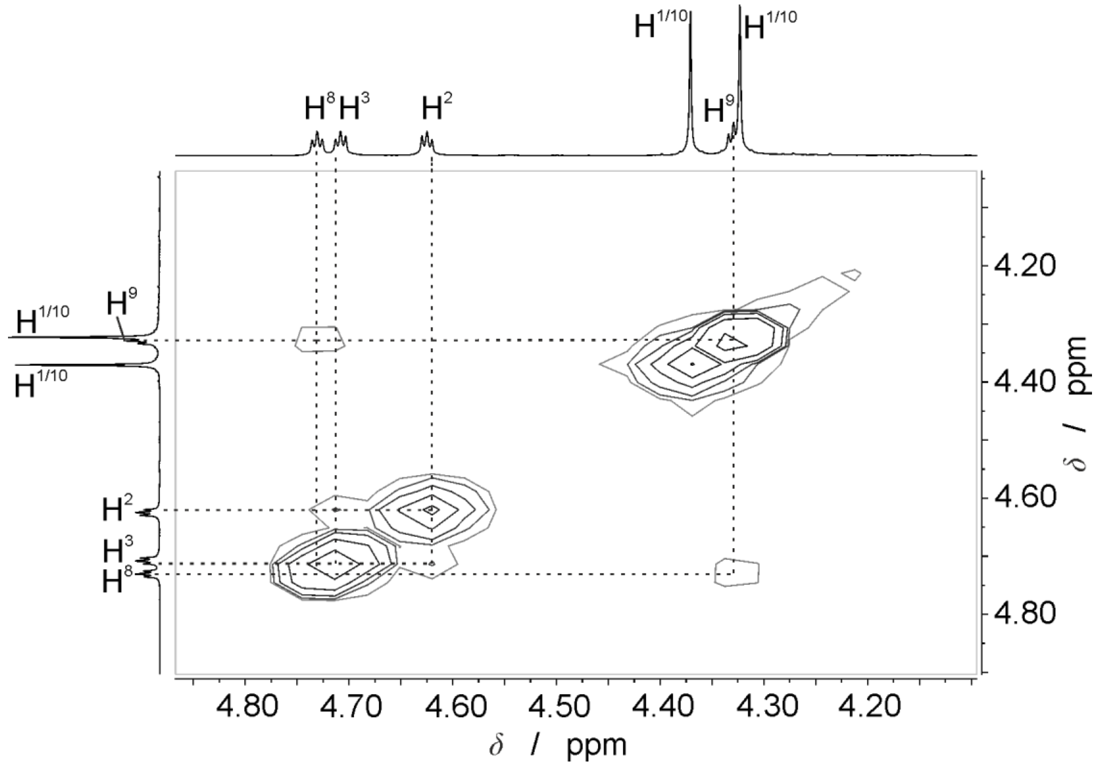

Figure S3: ^{1}H–^{1}H COSY of **W(CO)$_5$(*E*-2)** in CD_2Cl_2.

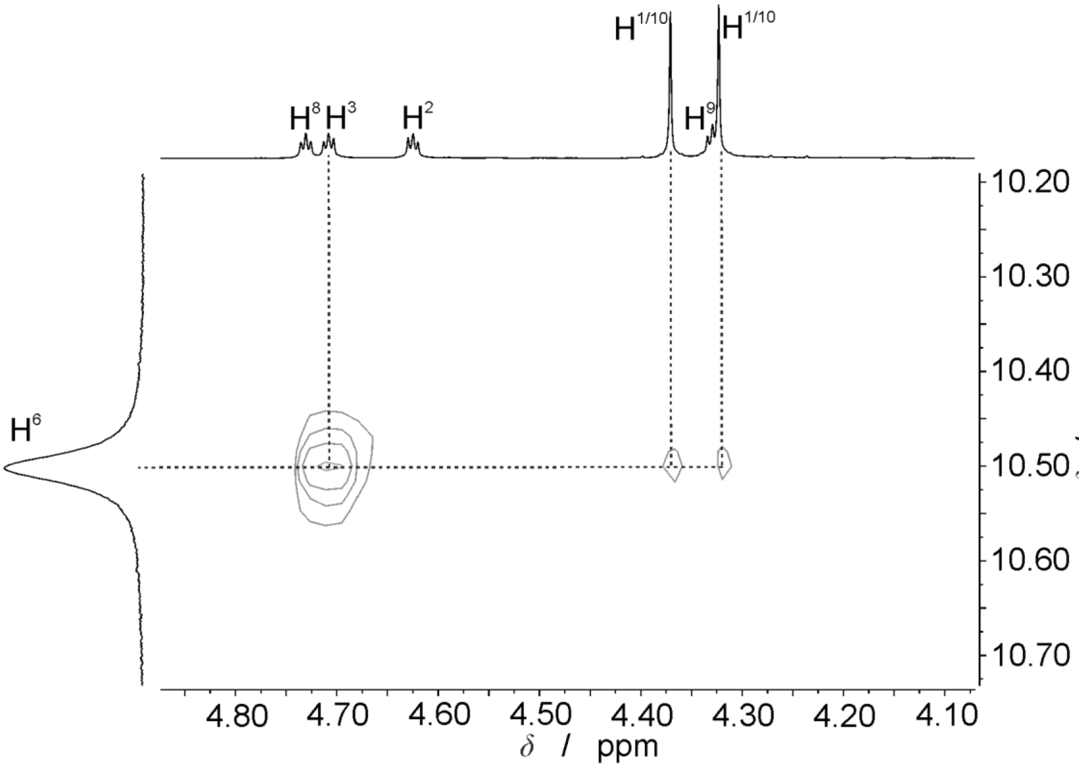

Figure S4: ^{1}H–^{1}H NOESY of **W(CO)$_5$(*E*-2)** in CD_2Cl_2.

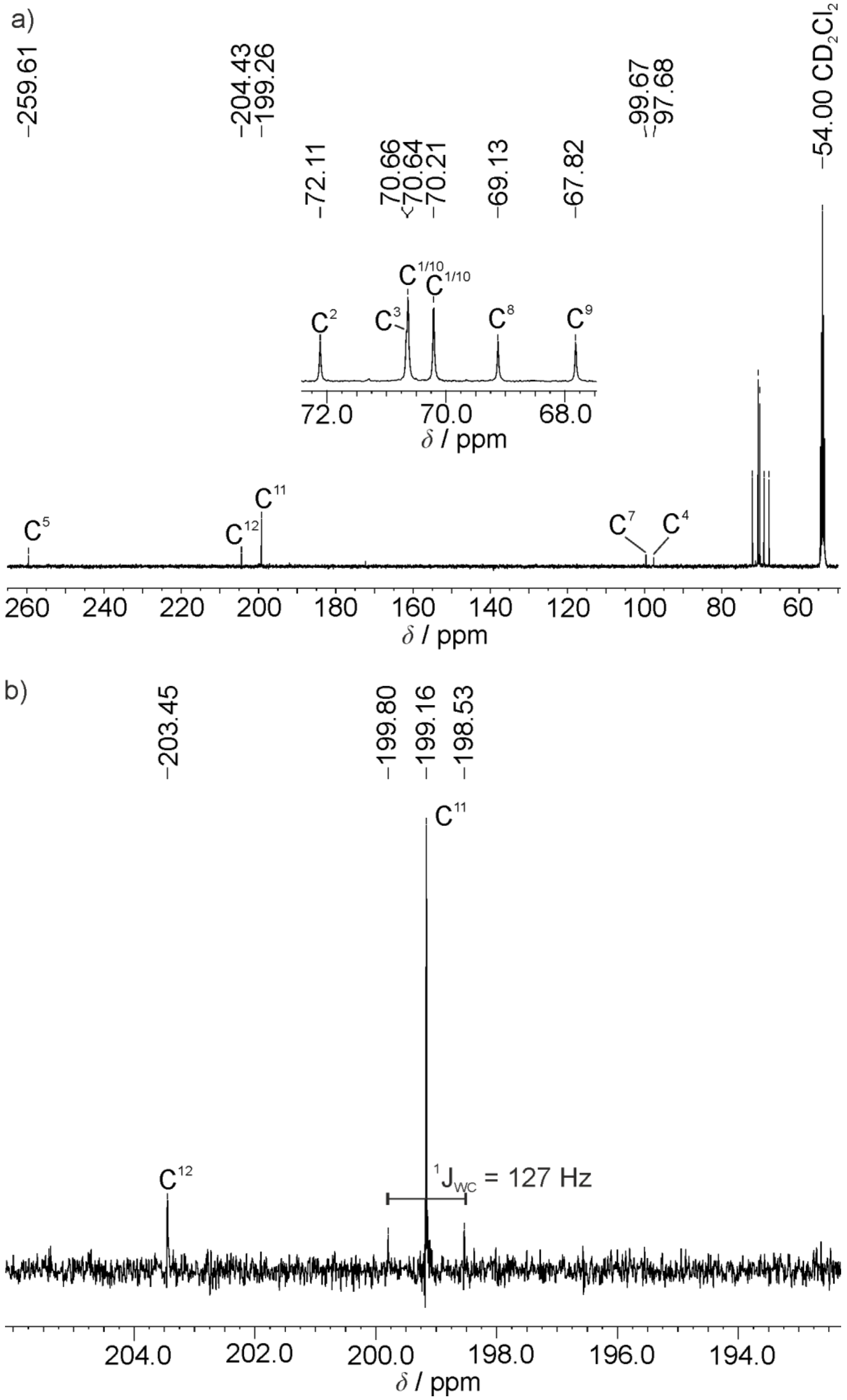

Figure S5: a) $^{13}C\{^1H\}$ NMR spectrum of **W(CO)$_5$(*E*-2)** in CD_2Cl_2 and b) zoom into the CO region showing the ^{183}W satellites in the $^{13}C\{^1H\}$ NMR spectrum of **W(CO)$_5$(*E*-2)** in d_8-toluene.

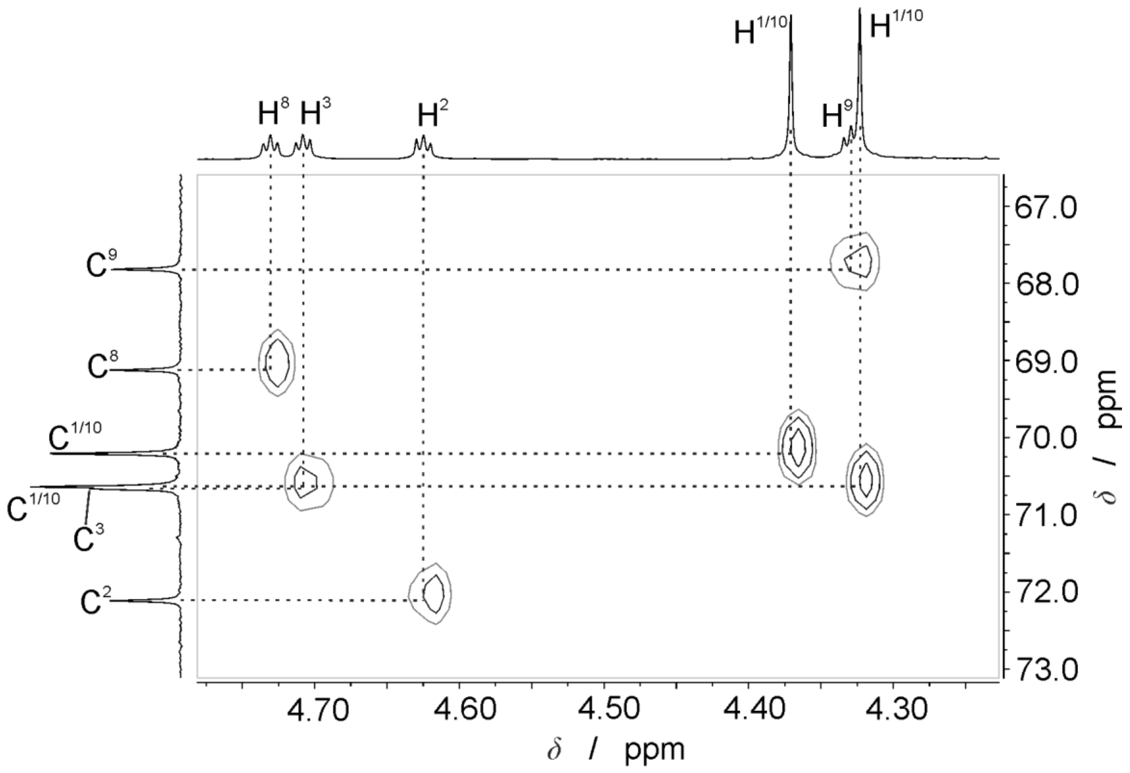

Figure S6: ^{13}C–^{1}H HSQC of **W(CO)$_5$(*E*-2)** in CD_2Cl_2.

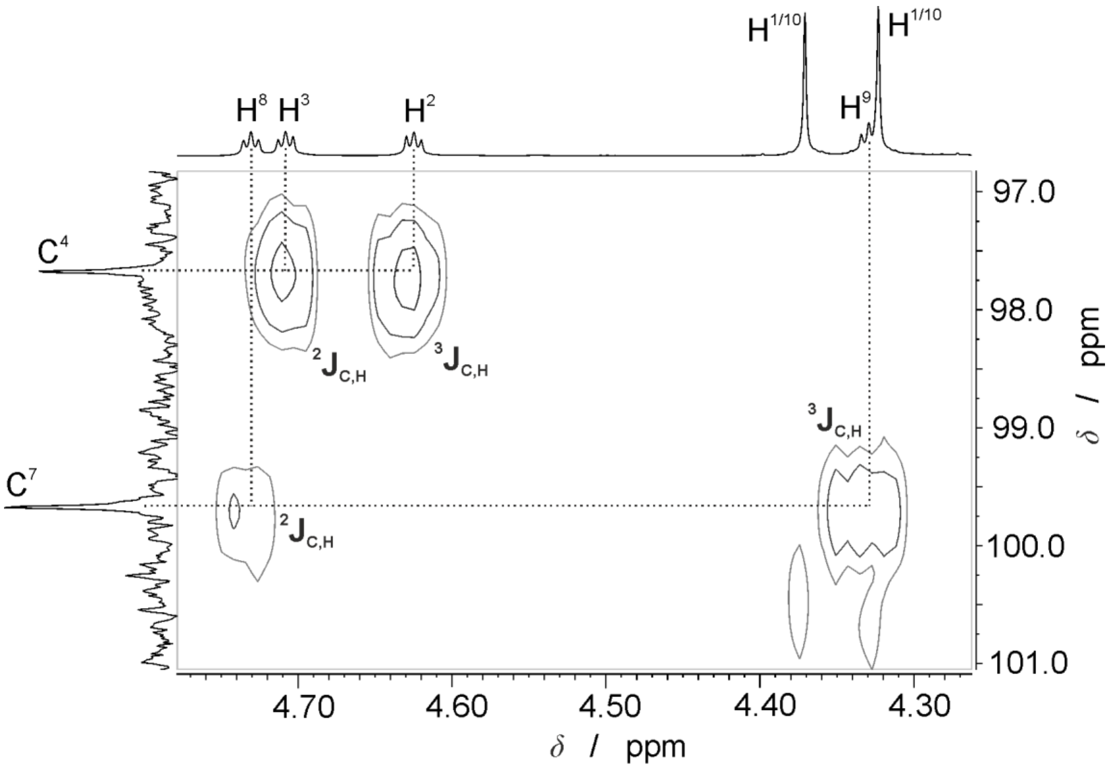

Figure S7: ^{13}C–^{1}H HMBC of **W(CO)$_5$(*E*-2)** in CD_2Cl_2.

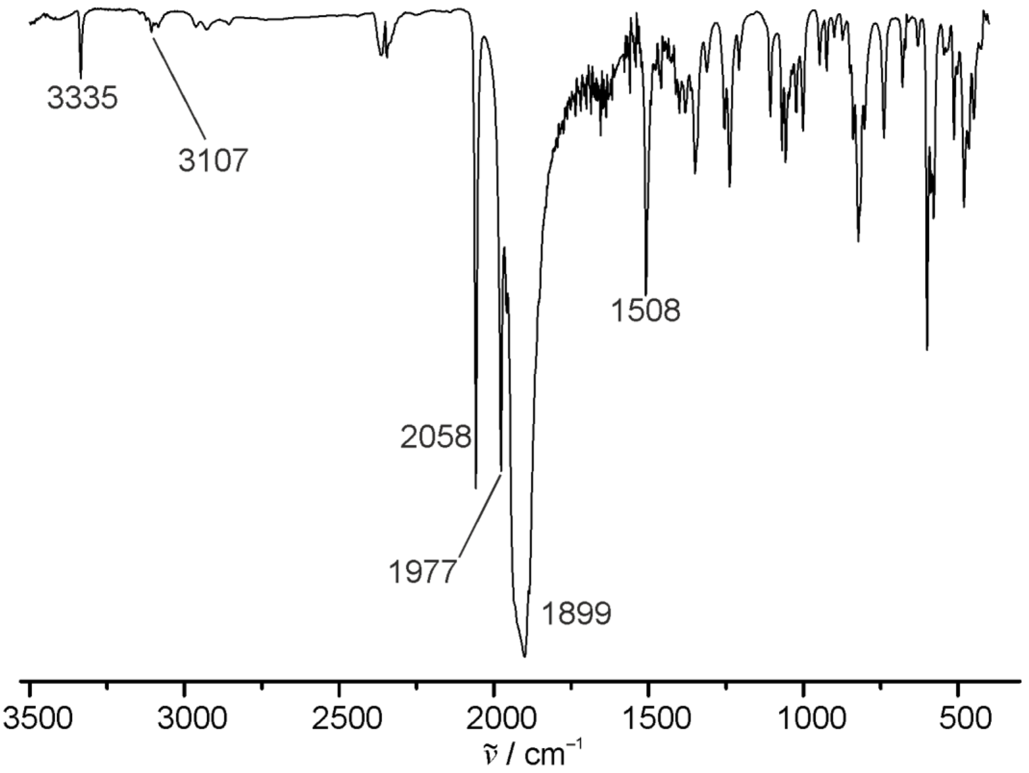

Figure S8: IR spectrum of **W(CO)$_5$(*E*-2)** as KBr disk.

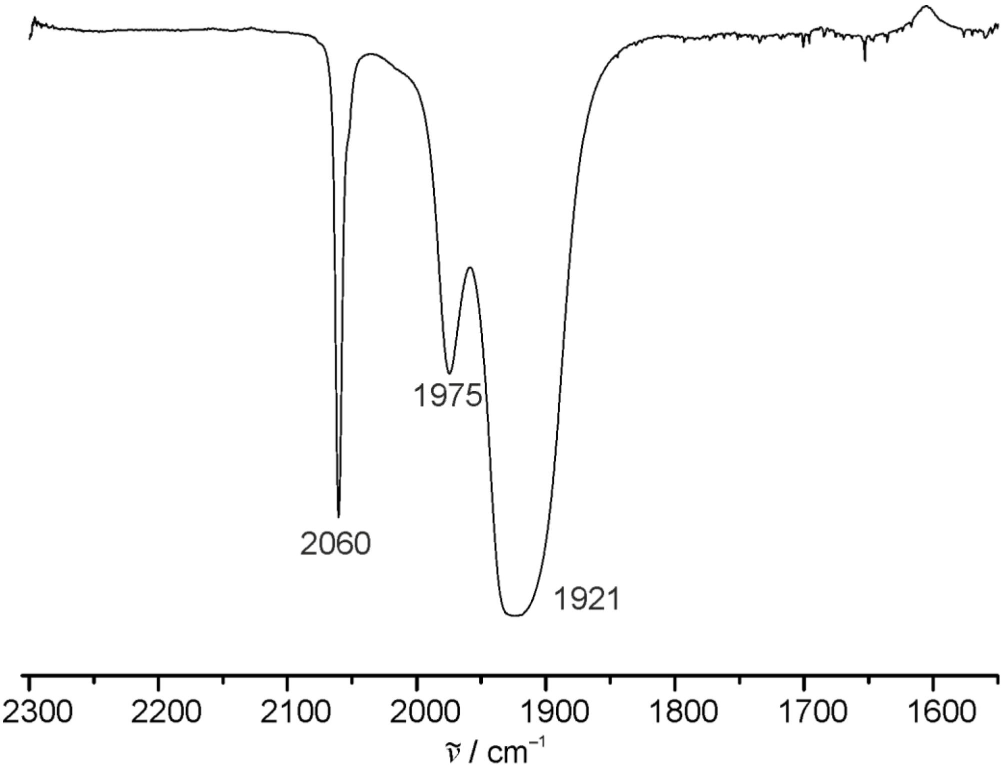

Figure S9: IR spectrum of **W(CO)$_5$(*E*-2)** in CH_2Cl_2 (CO region).

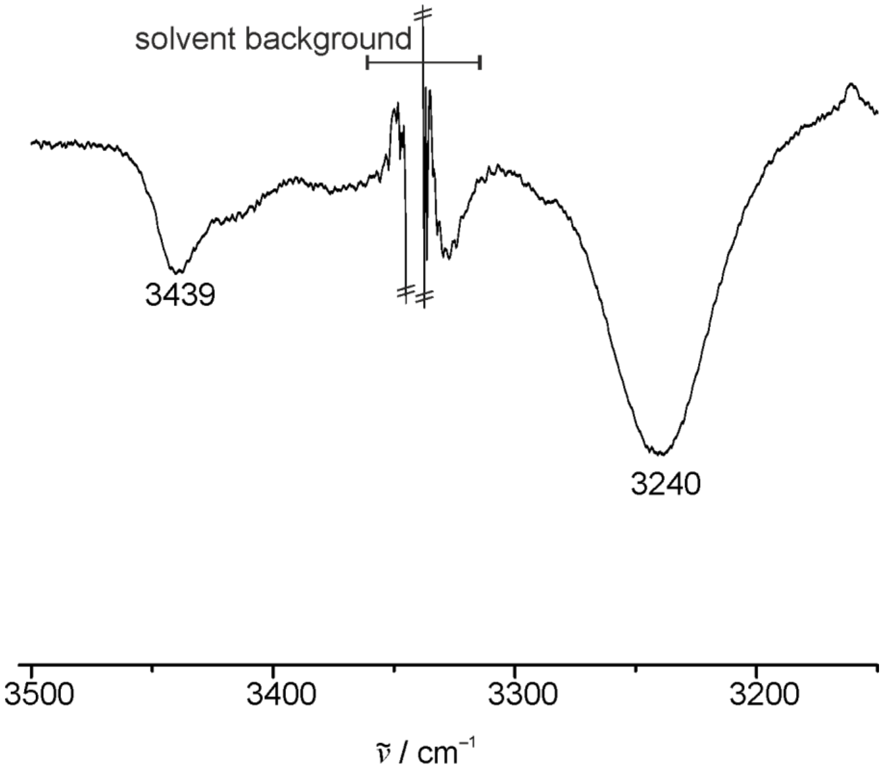

Figure S10: IR spectrum of **W(CO)$_5$(*E*-2)** in CD_2Cl_2 (NH region).

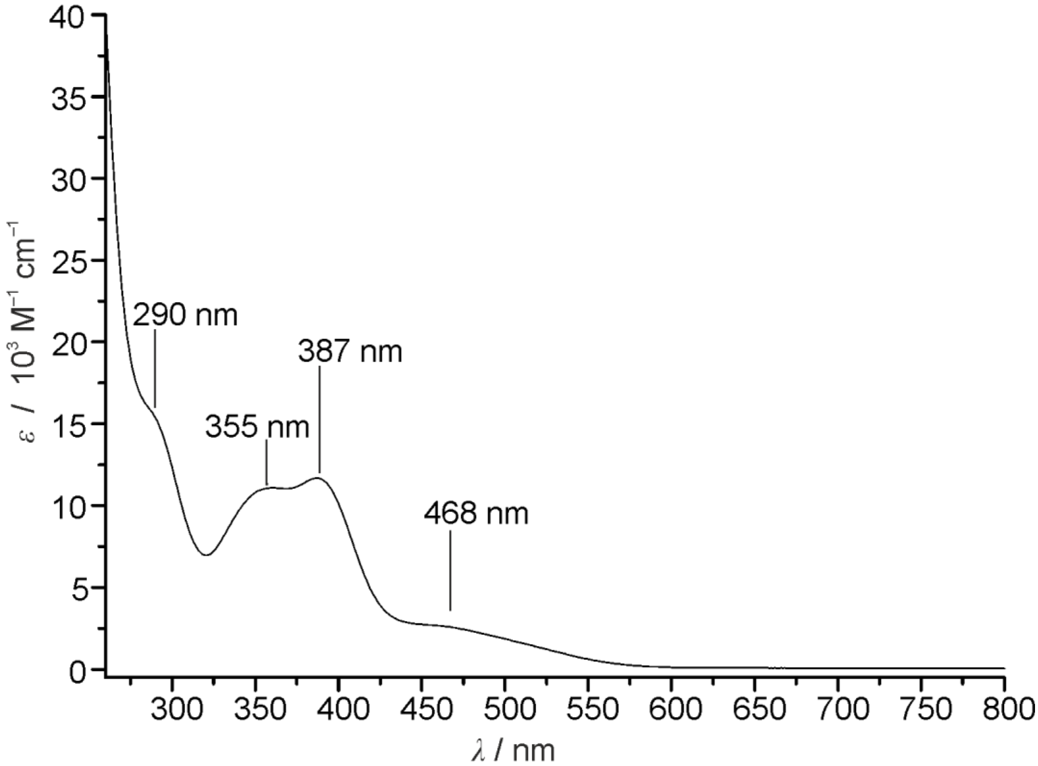

Figure S11: UV–vis spectrum of **W(CO)$_5$(*E*-2)** in CH_2Cl_2.

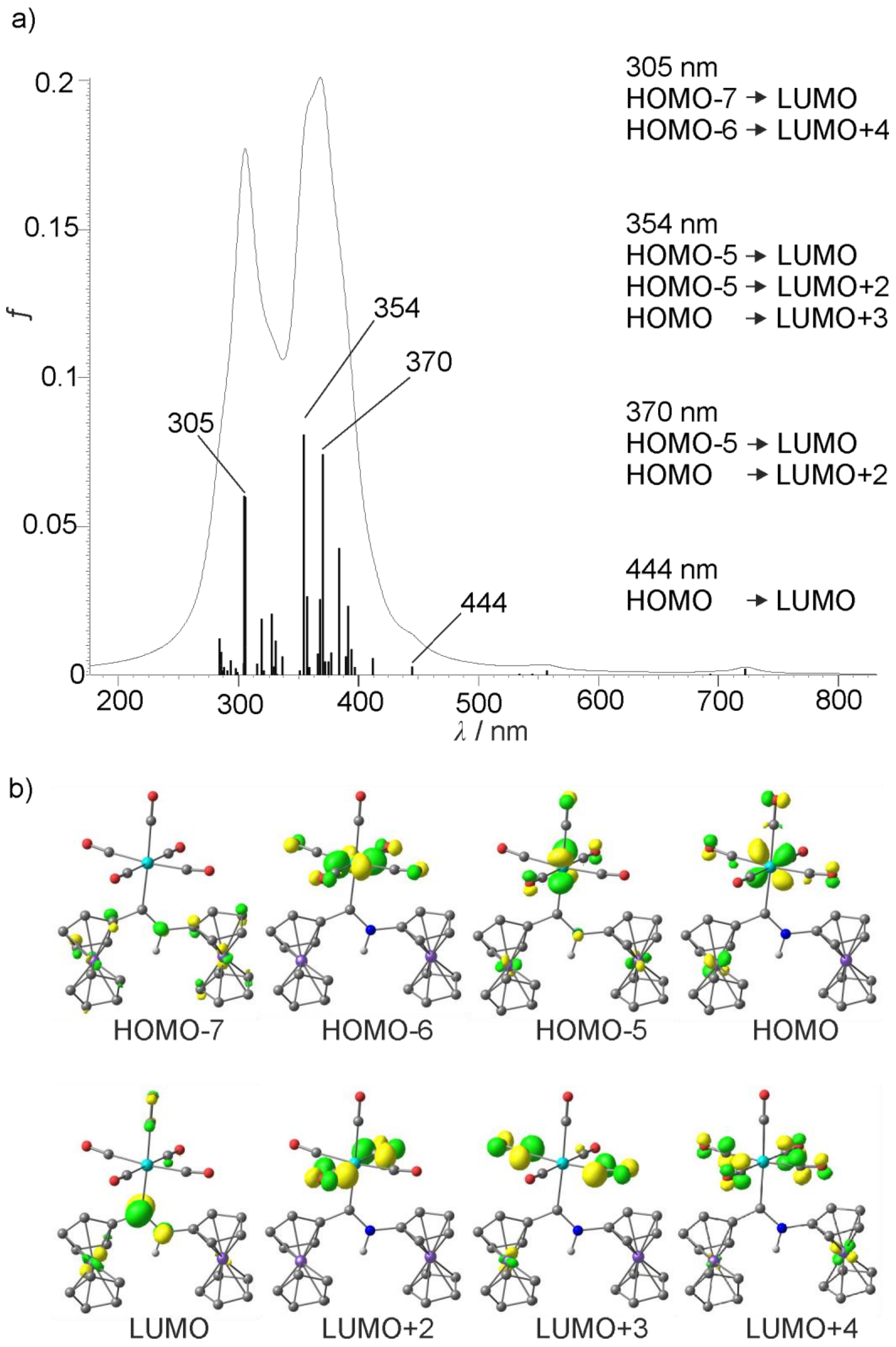

Figure S12: (a) TD-DFT calculated UV/Vis spectrum of **$W(CO)_5$(*E*-2)** with major orbital contributions to the indicated transitions and (b) corresponding orbitals (isosurface values 0.08 a.u.).

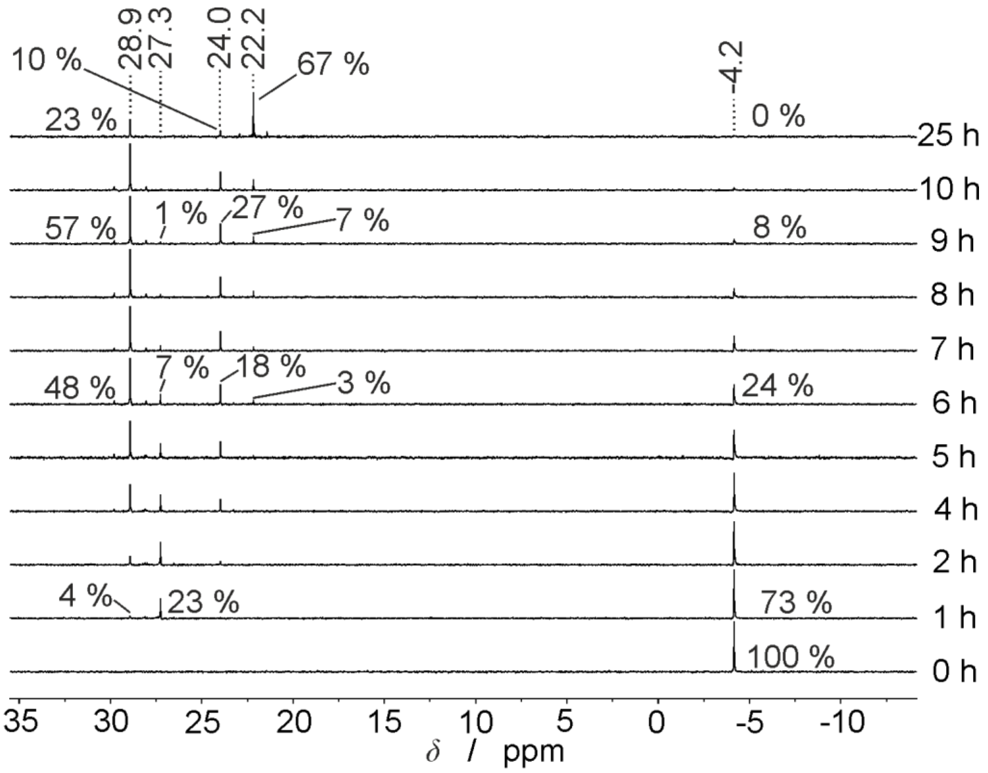

Figure S13: $^{31}P\{^1H\}$ NMR spectra of a solution of **W(CO)$_5$(*E*-2)** and 1 equiv PPh_3 in d_8-toluene during heating to 100 °C.

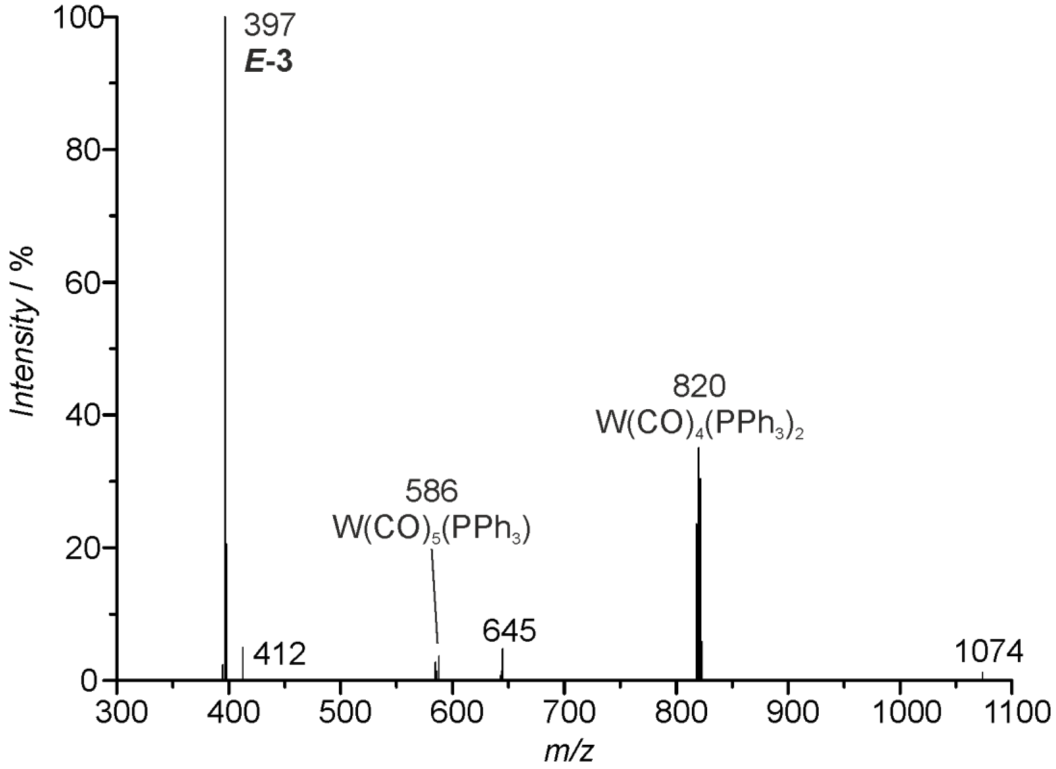

Figure S14: FD mass spectrum of a solution of **W(CO)$_5$(*E*-2)** and 1 equiv PPh_3 in d_8-toluene after heating to 100 °C for 25 h.

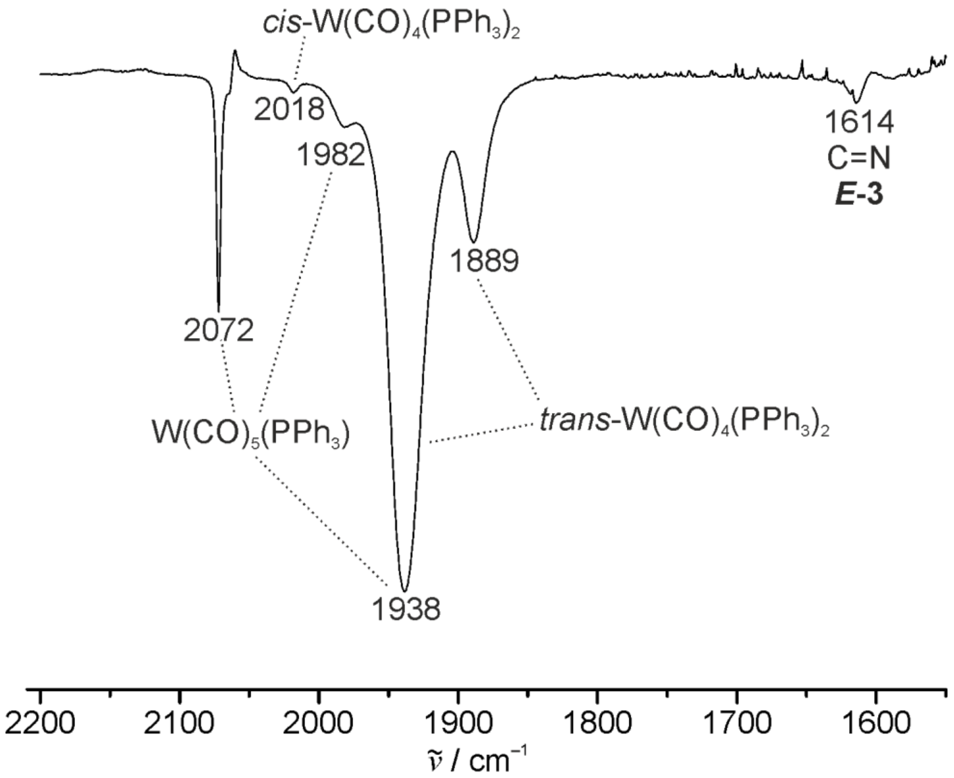

Figure S15: IR spectrum of a solution of **$W(CO)_5$(*E*-2)** and 1 equiv PPh_3 in d_8-toluene after heating to 100 °C for 25 h.

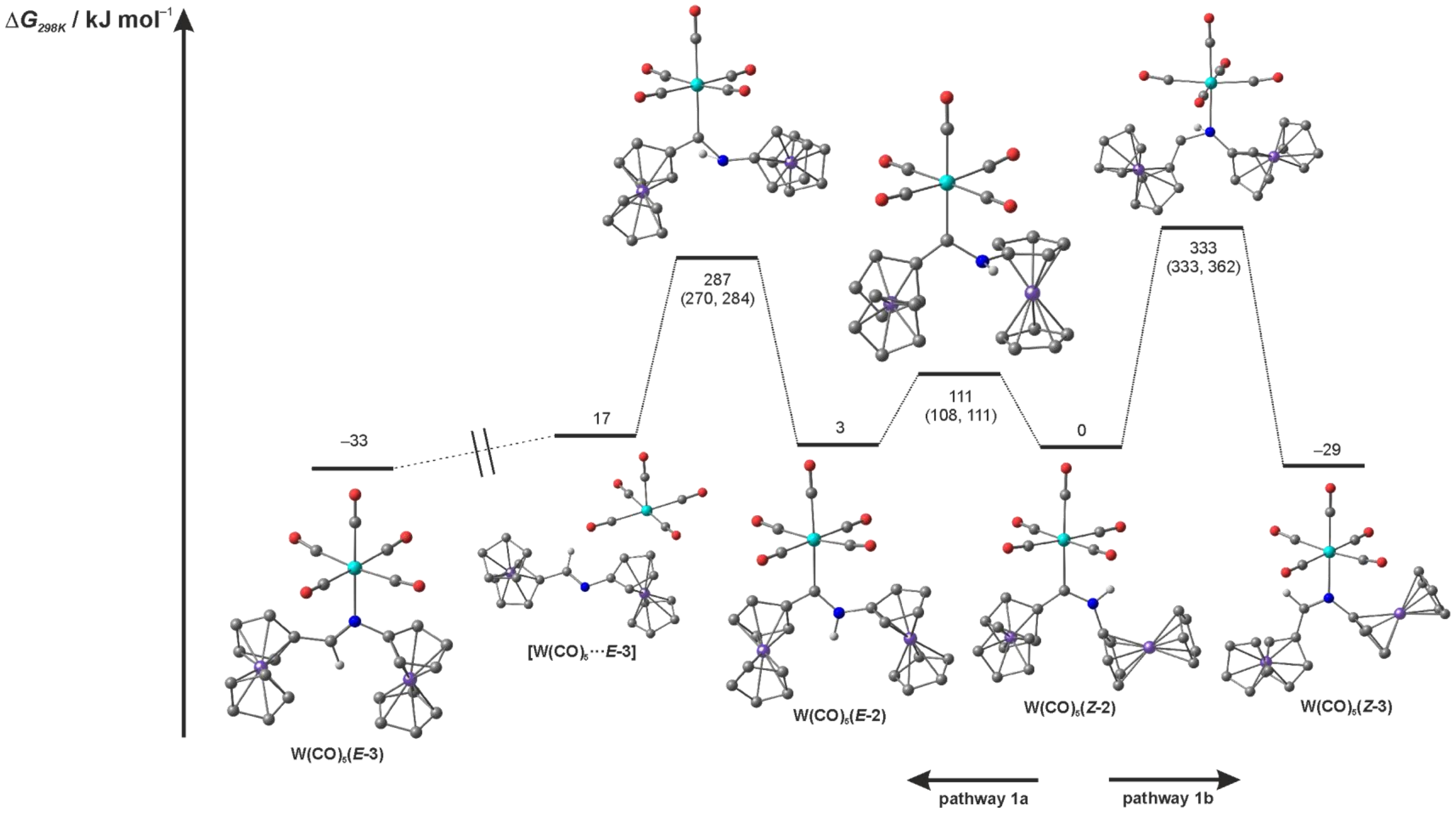

Figure S16: DFT calculated reaction pathway 1a from **$W(CO)_5$(*Z*-2)** to **$W(CO)_5$(*E*-3)** (left) and pathway 1b from **$W(CO)_5$(*Z*-2)** to **$W(CO)_5$(*Z*-3)** (right).

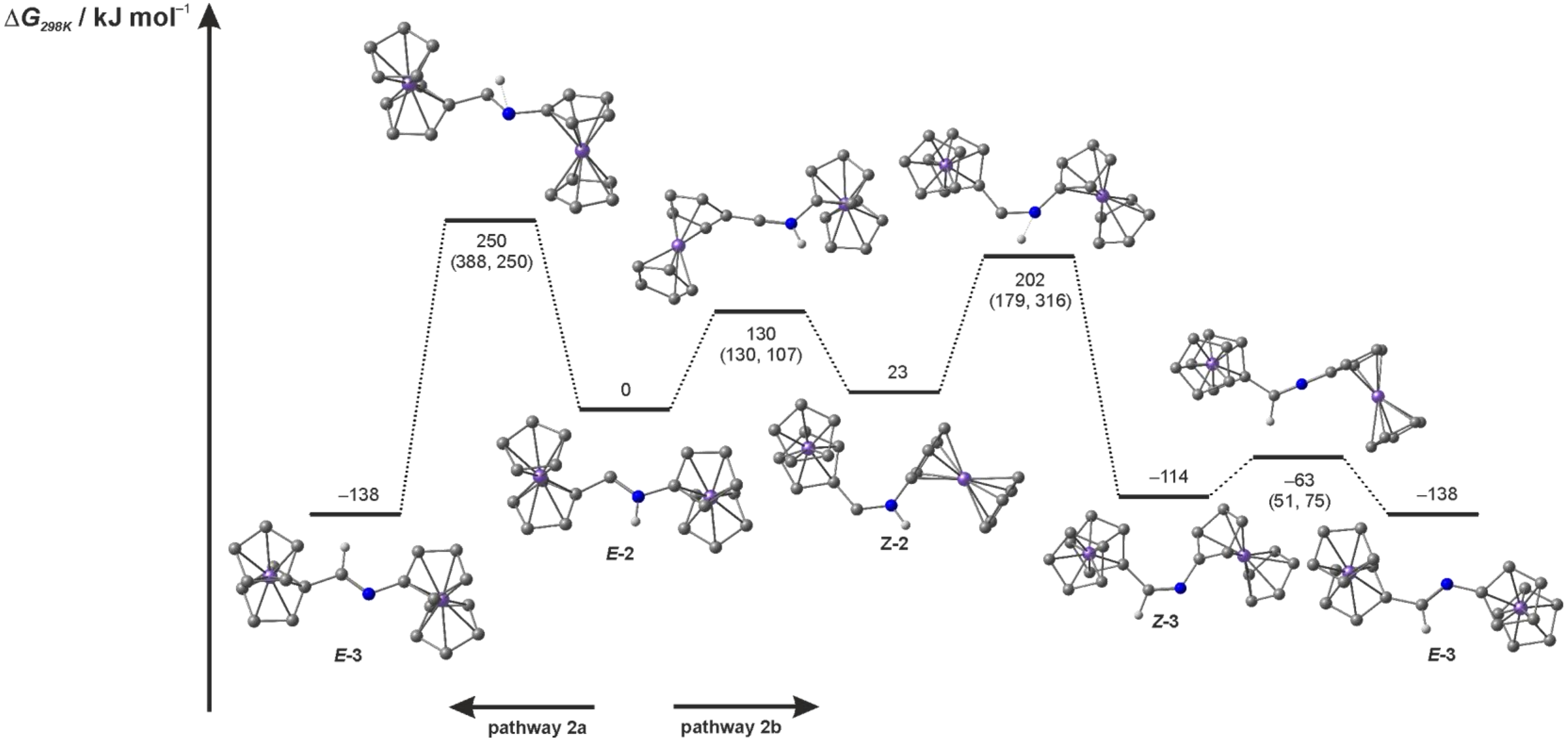

Figure S17: DFT calculated reaction pathway 2a from carbene ***E*-2** to imine ***E*-3** (left) and pathway 2b from carbene ***E*-2** to imine ***E*-3** via imine ***Z*-3** (right).

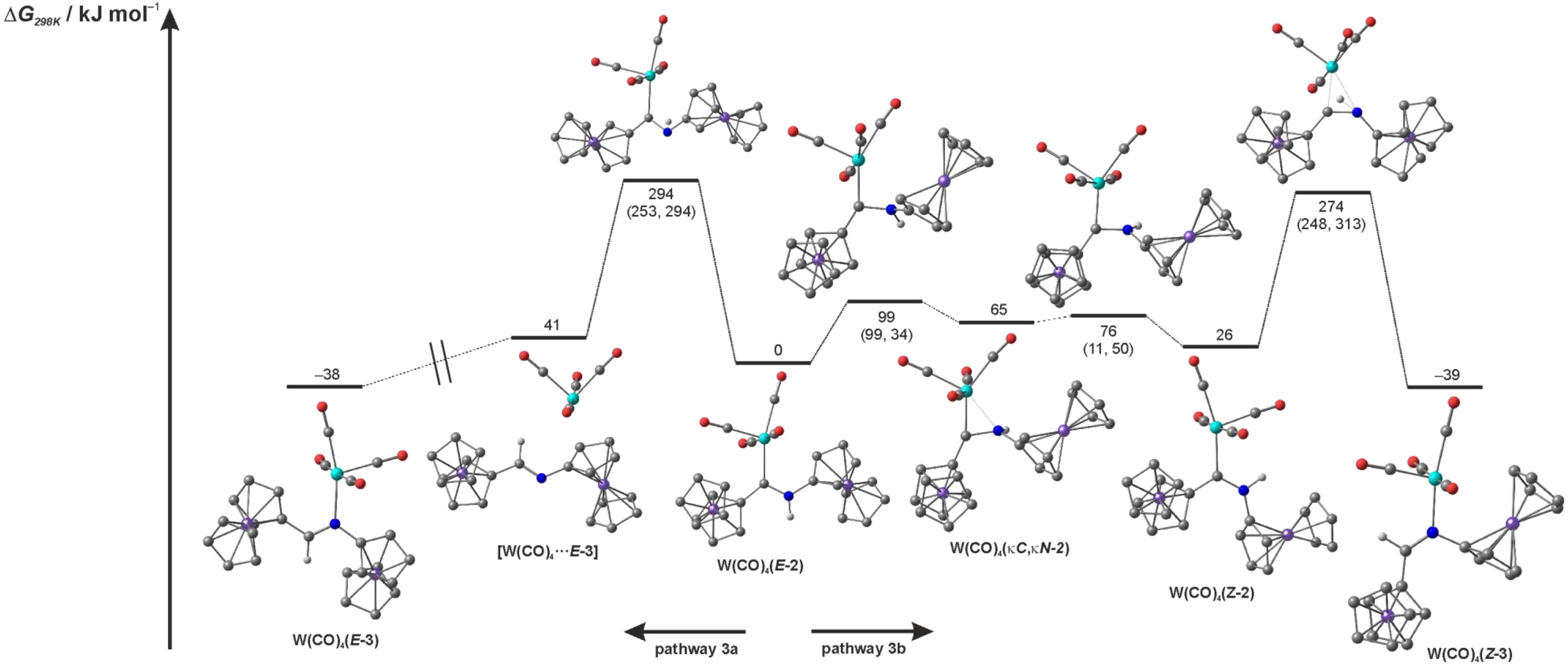

Figure S18: DFT calculated reaction pathway 3a from **W(CO)$_4$(*E*-2)** to **W(CO)$_4$(*E*-3)** (left) and pathway 3b **W(CO)$_4$(*E*-2)** to **W(CO)$_4$(*Z*-3)** (right).

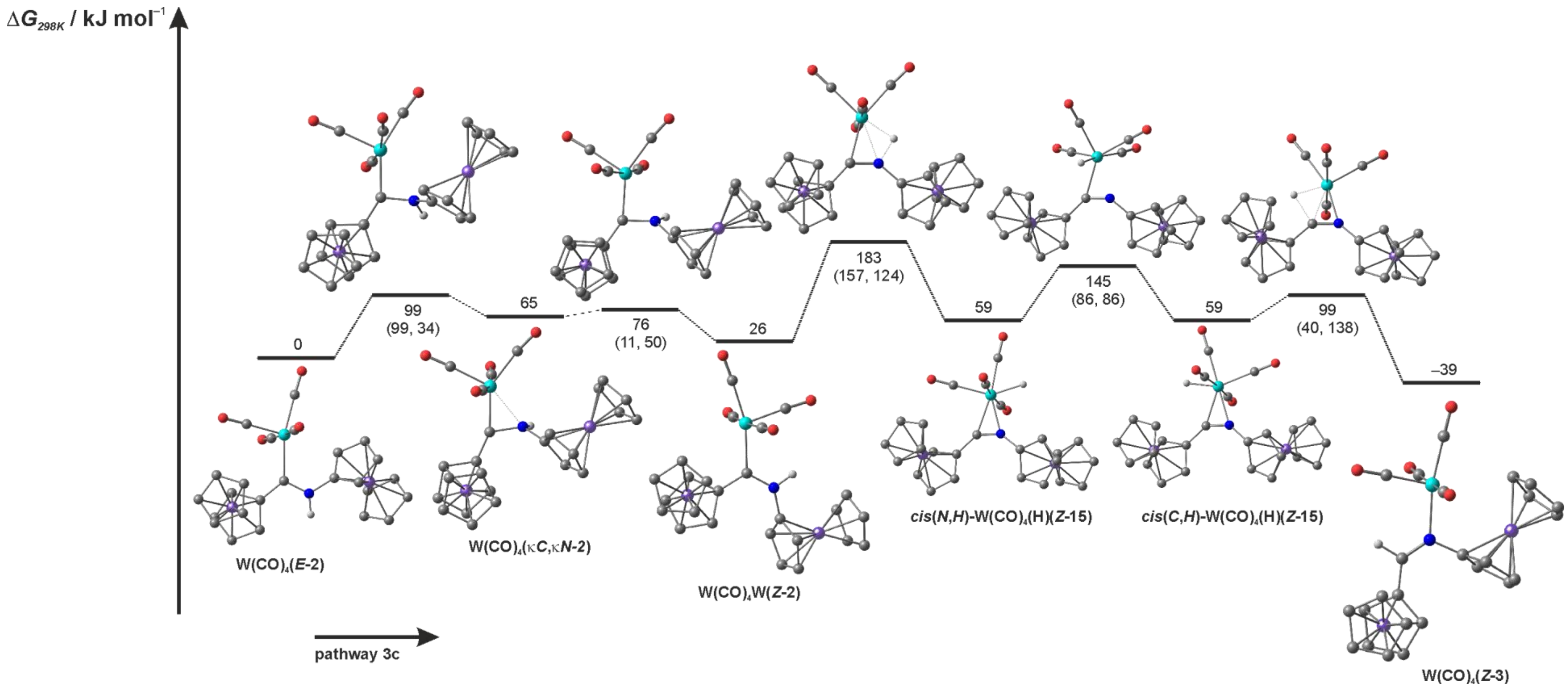

Figure S19: DFT calculated reaction pathway 3c from **$W(CO)_4$(*E*-2)** to **$W(CO)_4$(*Z*-3)** via oxidative addition/reductive elimination.

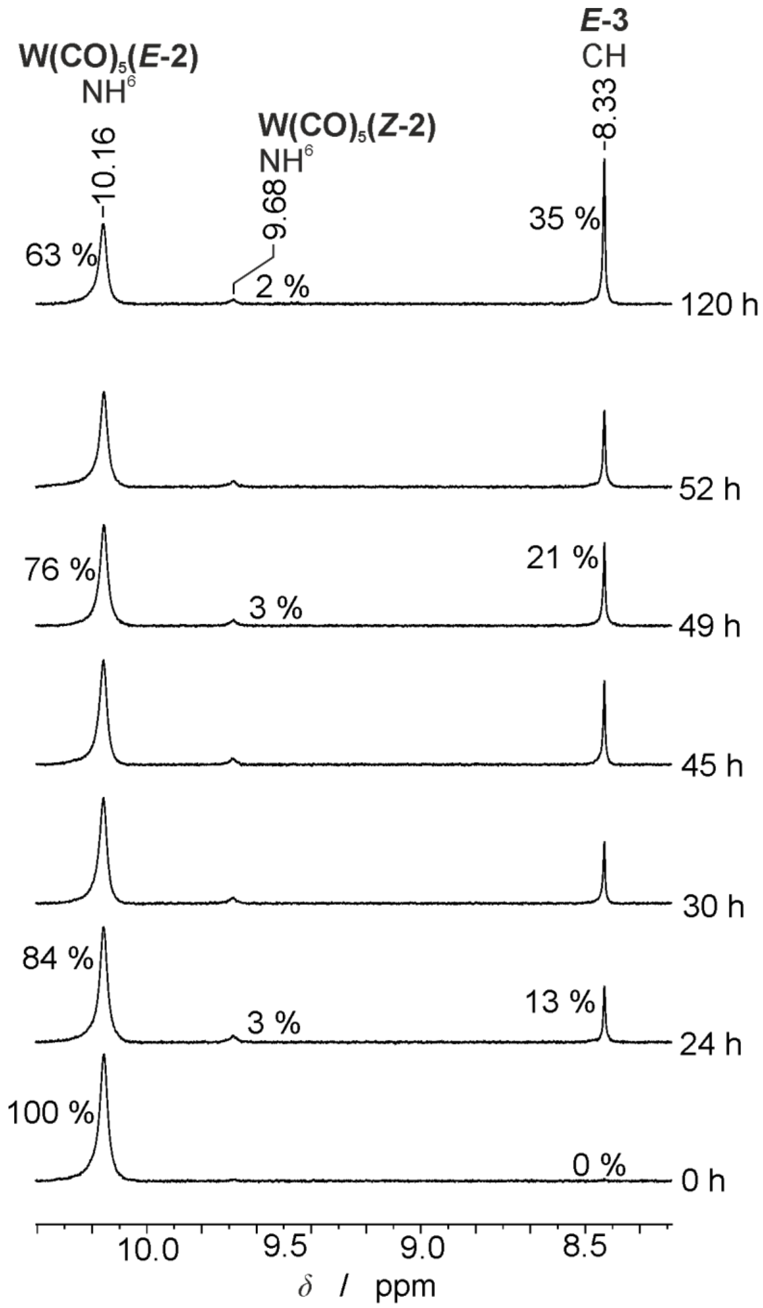

Figure S20: ^{1}H NMR spectroscopic reaction monitoring of the thermolysis of **W(CO)$_5$(*E*-2)** in d$_8$-toluene at 60 °C.

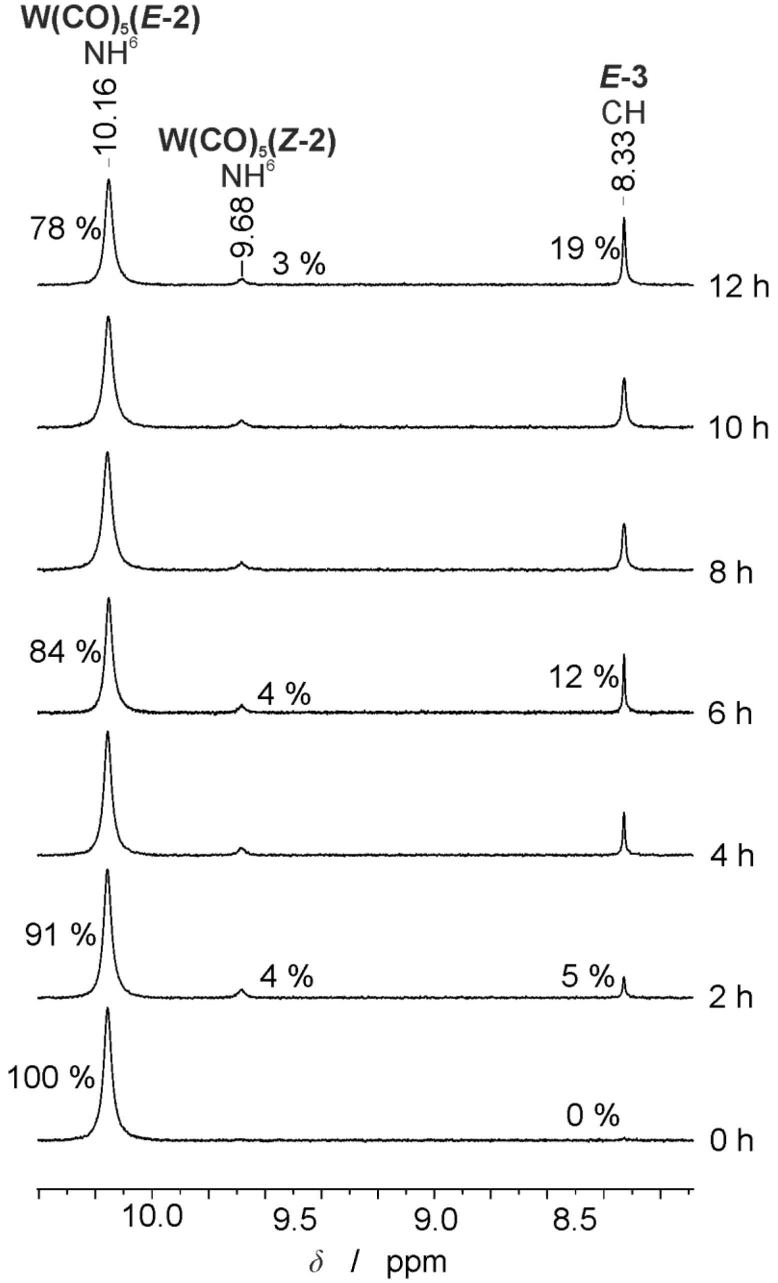

Figure S21: ^{1}H NMR spectroscopic reaction monitoring of the thermolysis of **W(CO)$_5$(*E*-2)** in d_8-toluene at 70 °C.

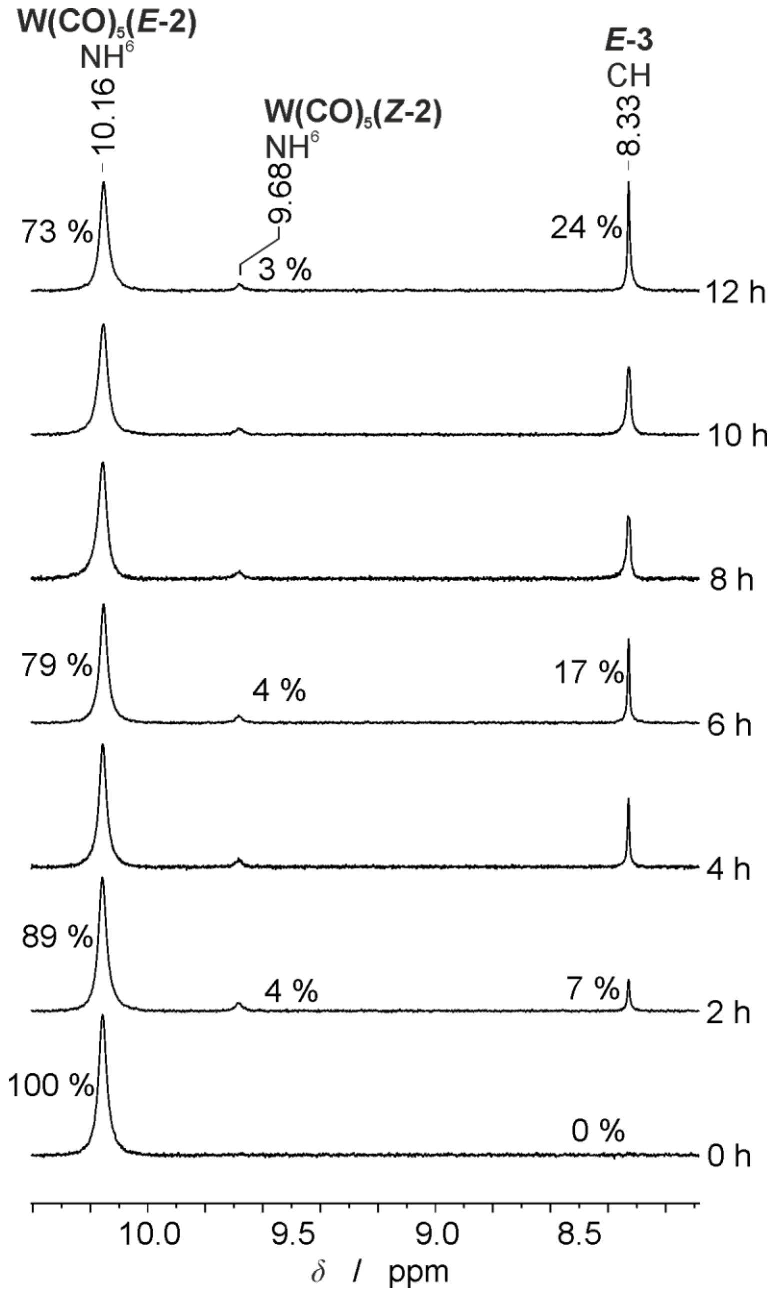

Figure S22: ^{1}H NMR spectroscopic reaction monitoring of the thermolysis of **$W(CO)_5$(*E*-2)** in d_8-toluene at 80 °C.

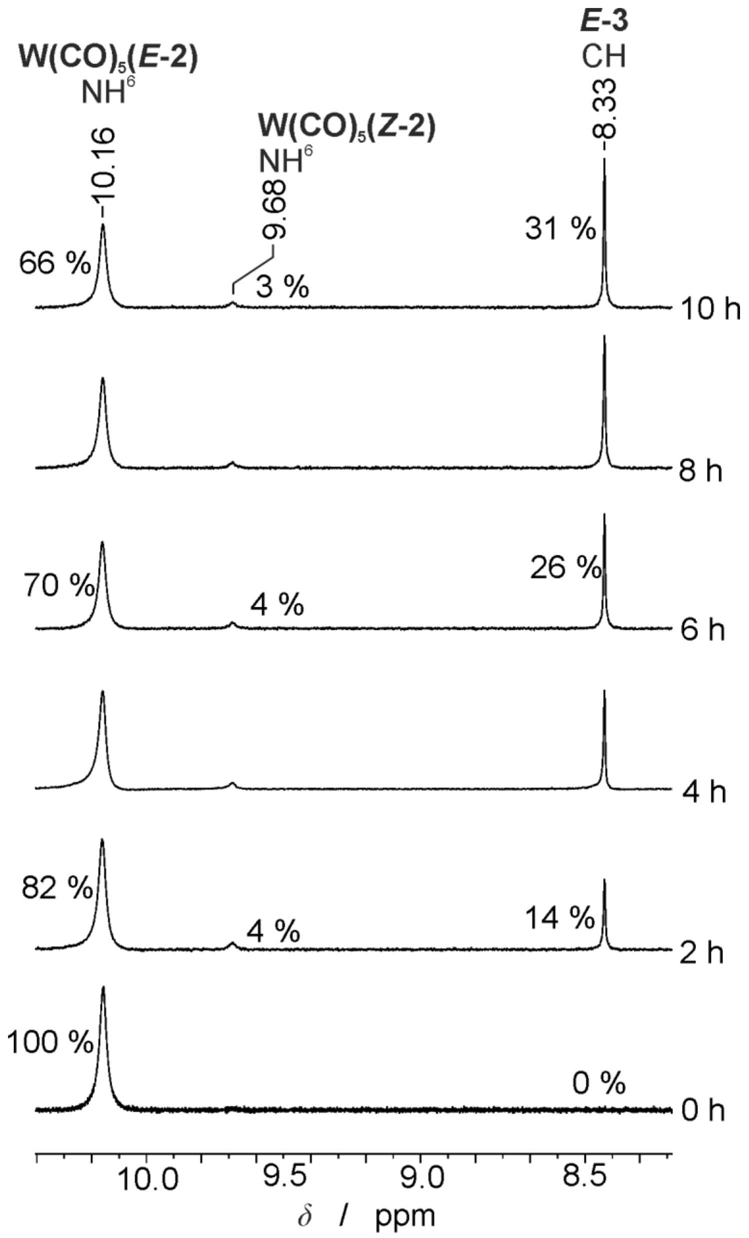

Figure S23: ^{1}H NMR spectroscopic reaction monitoring of the thermolysis of **$W(CO)_5$(*E*-2)** in d_8-toluene at 90 °C.

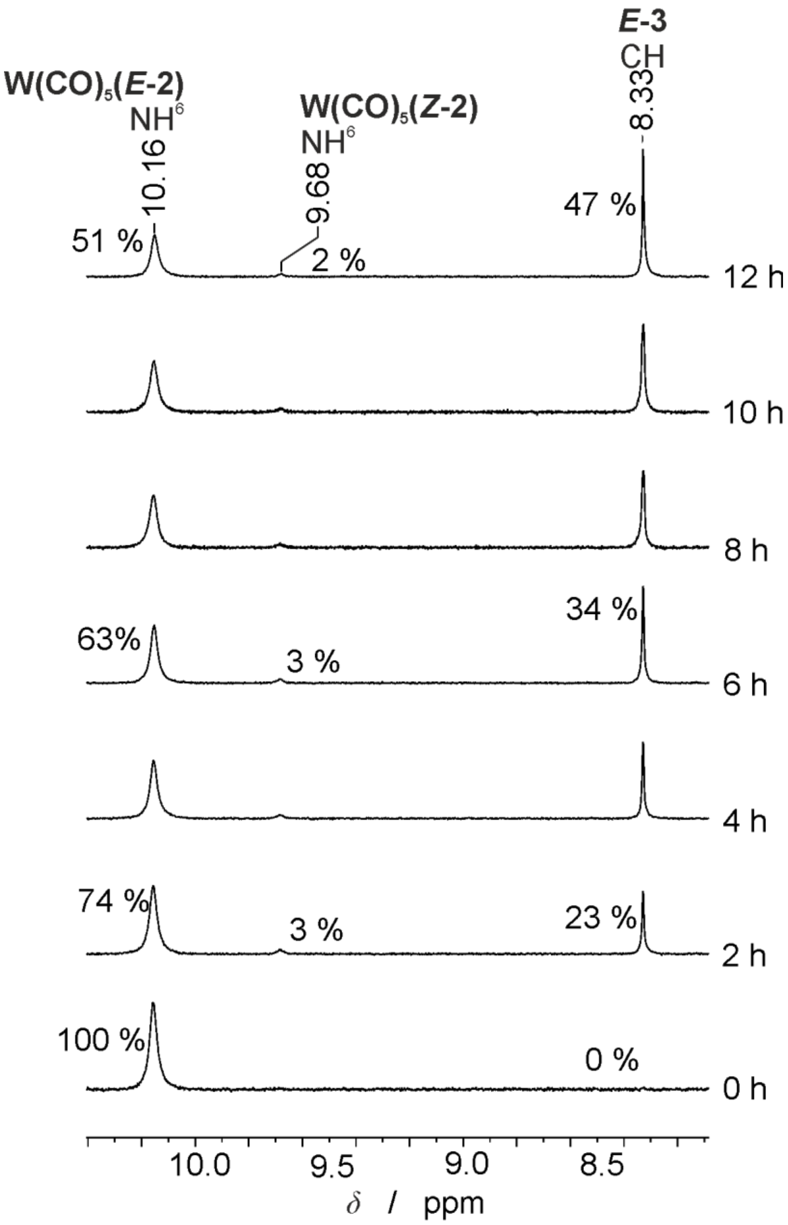

Figure S24: ^{1}H NMR spectroscopic reaction monitoring of the thermolysis of **W(CO)$_5$(*E*-2)** in d_8-toluene at 100 °C.

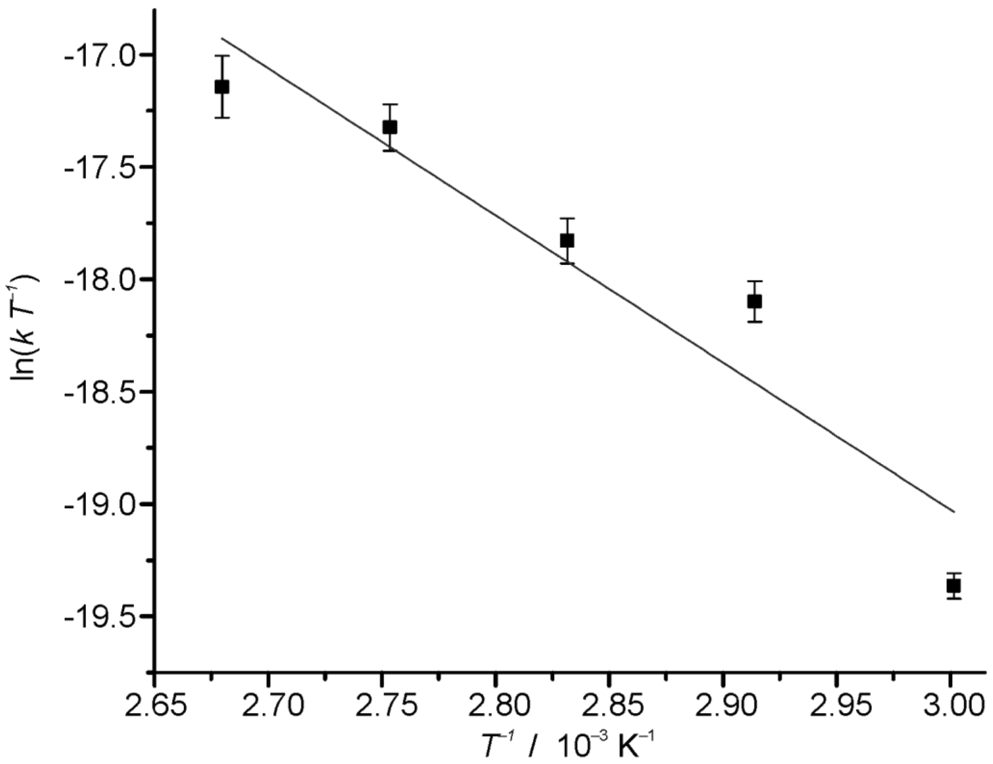

Figure S25: Eyring analysis of the ^{1}H NMR spectroscopic reaction monitoring of the thermolysis of **W(CO)$_5$(*E*-2)** in d_8-toluene at 60 °C, 70 °C, 80 °C, 90 °C and 100 °C.

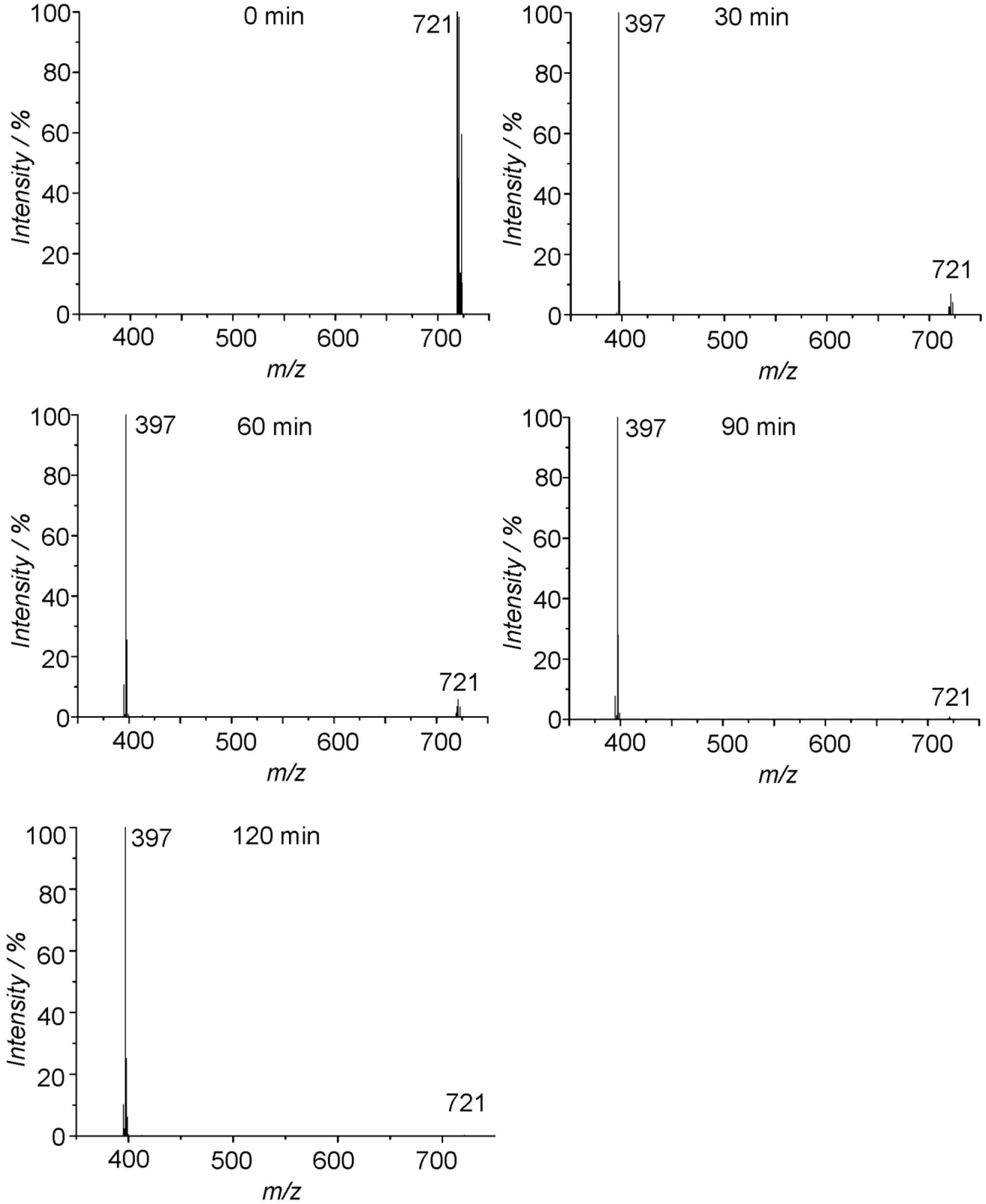

Figure S26: FD mass spectrometric reaction monitoring of the thermolysis of **$W(CO)_5$(*E*-2)** in toluene at 100 °C.

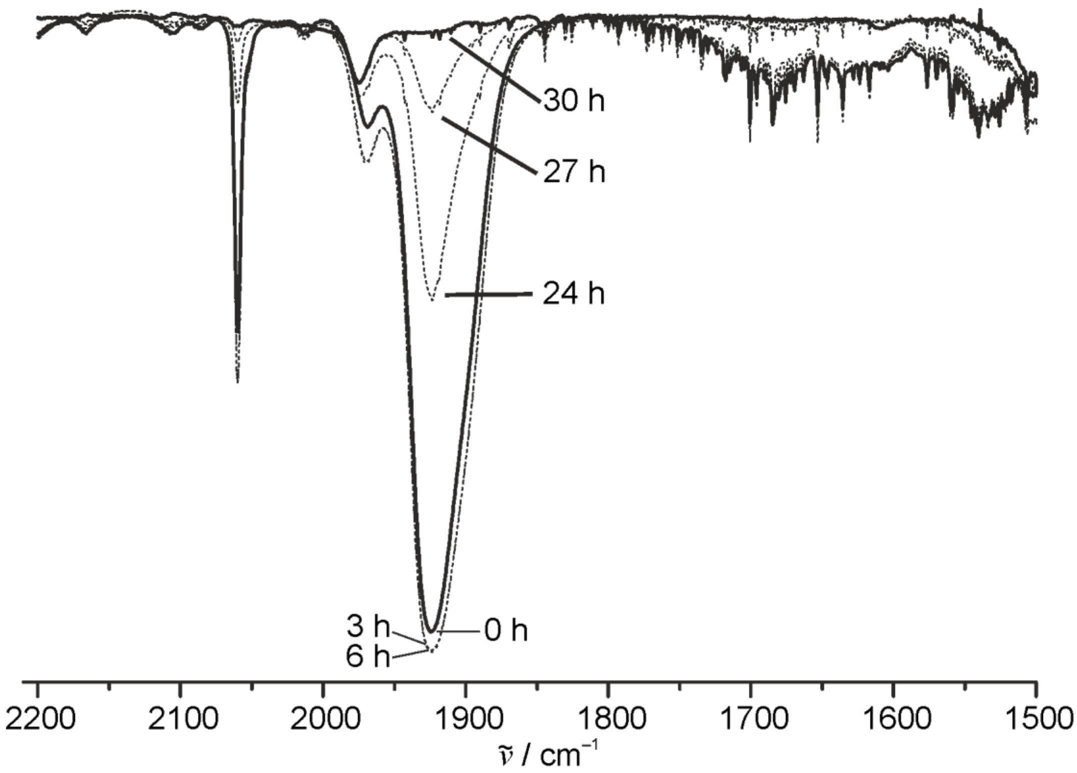

Figure S27: IR spectroscopic reaction monitoring of the thermolysis of **W(CO)$_5$(*E*-2)** in 1,2-dichloroethane at 84 °C.

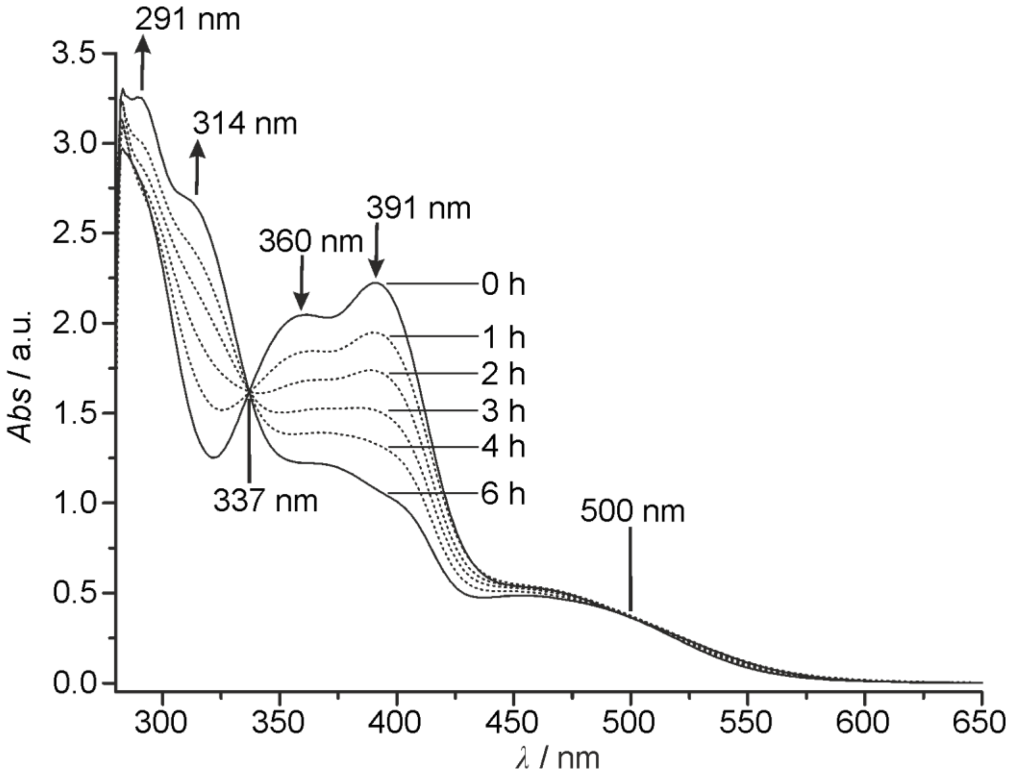

Figure S28: UV–vis spectroscopic reaction monitoring of the thermolysis of **W(CO)$_5$(*E*-2)** in toluene at 100 °C.

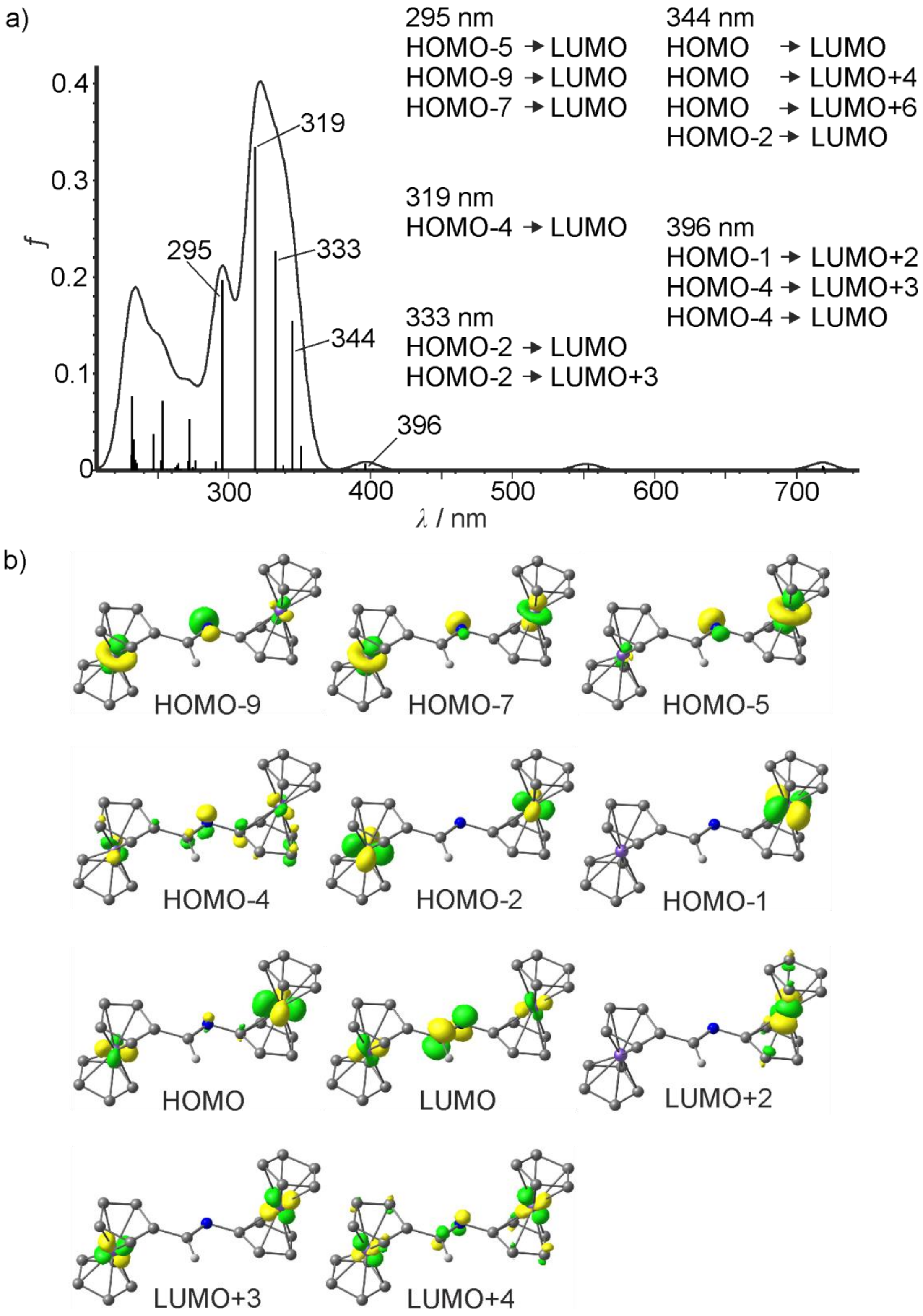

Figure S29: (a) TD-DFT calculated UV–vis spectrum of ***E*-3** with major orbital contributions to the indicated transitions and (b) corresponding orbitals (isosurface values 0.08 a.u.).

Cartesian Coordinates of DFT calculated structures

***E*-2**

Fe	-3.543162000	0.176245000	0.004948000
Fe	3.555481000	-0.185262000	0.002842000
C	-0.429386000	0.219846000	0.534909000
C	-1.597273000	0.990936000	0.091232000
C	-2.472624000	1.673695000	1.030899000
H	-2.310549000	1.701416000	2.098780000
C	-3.543183000	2.289948000	0.301749000
H	-4.351478000	2.870351000	0.723778000
C	-3.362492000	1.987416000	-1.100792000
H	-4.017379000	2.297008000	-1.903873000
C	-2.176587000	1.190098000	-1.240650000
H	-1.806479000	0.794182000	-2.177727000
C	-3.472072000	-1.896325000	0.469734000
H	-2.566381000	-2.433218000	0.714440000
C	-4.017671000	-1.706523000	-0.852268000
H	-3.605521000	-2.096925000	-1.772464000
C	-5.212046000	-0.903025000	-0.730284000
H	-5.848737000	-0.581891000	-1.543059000
C	-5.401673000	-0.596933000	0.667411000
H	-6.203034000	-0.002412000	1.083806000
C	-4.324111000	-1.208006000	1.408857000
H	-4.175111000	-1.154235000	2.478035000
C	1.570708000	-0.905327000	-0.295616000
C	2.093744000	-1.404843000	0.947980000
H	1.650776000	-1.231294000	1.915626000
C	3.271666000	-2.179790000	0.643123000
H	3.893247000	-2.691307000	1.364442000
C	3.481691000	-2.143001000	-0.784429000
H	4.282508000	-2.625896000	-1.326157000
C	2.430611000	-1.343280000	-1.368405000
H	2.296758000	-1.133183000	-2.421105000
C	3.537025000	1.932951000	0.027050000
H	2.657472000	2.543605000	-0.122915000
C	4.434229000	1.462693000	-1.002541000
H	4.350913000	1.667509000	-2.061033000
C	5.462375000	0.669946000	-0.371208000
H	6.280371000	0.172690000	-0.873999000
C	5.200183000	0.649482000	1.048527000
H	5.785597000	0.131644000	1.795617000
C	4.010234000	1.429932000	1.293965000
H	3.544516000	1.596139000	2.255239000
N	0.386235000	-0.139714000	-0.470760000
H	0.218480000	0.128908000	-1.450103000

***Z*-2**

Fe	3.237542000	-0.011804000	-0.013216000
Fe	-3.238681000	0.049308000	-0.189018000
C	0.740543000	-0.528526000	1.808891000

C	1.639267000	-1.119404000	0.807656000
C	2.918220000	-1.664227000	1.252150000
H	3.219846000	-1.712699000	2.288257000
C	3.663378000	-2.101295000	0.110119000
H	4.647756000	-2.547985000	0.121678000
C	2.871184000	-1.827116000	-1.068840000
H	3.167215000	-2.028252000	-2.089490000
C	1.634779000	-1.229546000	-0.656014000
H	0.848019000	-0.908962000	-1.319891000
C	3.086130000	2.013411000	0.602992000
H	2.312156000	2.387101000	1.258317000
C	3.015950000	1.929247000	-0.836156000
H	2.189191000	2.251544000	-1.453541000
C	4.243733000	1.331200000	-1.306921000
H	4.496619000	1.125312000	-2.337731000
C	5.069382000	1.045615000	-0.157944000
H	6.048476000	0.587119000	-0.177786000
C	4.352793000	1.465560000	1.023166000
H	4.697282000	1.376359000	2.043756000
C	-1.188497000	-0.276152000	0.163915000
C	-1.352044000	0.788758000	-0.798431000
H	-1.003396000	1.804748000	-0.677393000
C	-2.051178000	0.244672000	-1.935900000
H	-2.334621000	0.788835000	-2.825923000
C	-2.315047000	-1.151653000	-1.675909000
H	-2.831905000	-1.833214000	-2.336697000
C	-1.783534000	-1.475616000	-0.376206000
H	-1.808461000	-2.438591000	0.113616000
C	-4.203892000	0.548261000	1.630304000
H	-3.735612000	0.619844000	2.602833000
C	-4.804333000	-0.636705000	1.064660000
H	-4.848797000	-1.610772000	1.531615000
C	-5.330073000	-0.292972000	-0.234801000
H	-5.830720000	-0.967220000	-0.915766000
C	-5.055674000	1.104249000	-0.472261000
H	-5.316534000	1.661096000	-1.361565000
C	-4.359909000	1.624589000	0.680291000
H	-4.015384000	2.641287000	0.809011000
N	-0.507233000	-0.166804000	1.436868000
H	-1.067946000	0.242889000	2.178160000

***E*-3**

Fe	3.561997000	0.179846000	0.012418000
Fe	-3.565445000	-0.182186000	0.009169000
C	0.431624000	0.172932000	-0.628213000
C	1.623336000	0.968180000	-0.352840000
C	2.561084000	1.443426000	-1.353801000
H	2.480627000	1.280258000	-2.420128000
C	3.594712000	2.185254000	-0.682271000
H	4.435981000	2.672516000	-1.154670000
C	3.311399000	2.161988000	0.735325000

H	3.909558000	2.624528000	1.507867000
C	2.104321000	1.407999000	0.940972000
H	1.619062000	1.201999000	1.883294000
C	3.497879000	-1.941293000	-0.038523000
H	2.608981000	-2.523318000	-0.238879000
C	3.938069000	-1.491706000	1.259603000
H	3.441196000	-1.685097000	2.199918000
C	5.150881000	-0.728309000	1.078877000
H	5.721899000	-0.248039000	1.861279000
C	5.457769000	-0.705130000	-0.331474000
H	6.300493000	-0.206561000	-0.790041000
C	4.434799000	-1.453710000	-1.022895000
H	4.380882000	-1.620291000	-2.089808000
C	-1.567470000	-0.913919000	0.009740000
C	-2.212654000	-1.184760000	-1.267452000
H	-1.900753000	-0.823329000	-2.237397000
C	-3.356748000	-2.022929000	-1.015198000
H	-4.044078000	-2.396345000	-1.761771000
C	-3.438840000	-2.261041000	0.407440000
H	-4.193558000	-2.849363000	0.909943000
C	-2.347001000	-1.567978000	1.038427000
H	-2.113421000	-1.542595000	2.092925000
C	-3.535335000	1.908243000	0.355027000
H	-2.634954000	2.488134000	0.502698000
C	-4.296558000	1.249255000	1.389352000
H	-4.071365000	1.259823000	2.446611000
C	-5.402983000	0.565555000	0.762733000
H	-6.152360000	-0.027533000	1.268517000
C	-5.324210000	0.799457000	-0.659687000
H	-6.006657000	0.416159000	-1.405721000
C	-4.169528000	1.629919000	-0.911152000
H	-3.836081000	1.977984000	-1.879042000
N	-0.410295000	-0.163787000	0.305407000
H	0.288998000	-0.120185000	-1.678652000

***Z*-3**

Fe	-3.379184000	0.140678000	0.035444000
Fe	3.387821000	-0.161259000	0.141316000
C	-0.561854000	0.469061000	-1.343379000
C	-1.473708000	1.005019000	-0.319903000
C	-1.619648000	0.668880000	1.085793000
H	-1.000629000	-0.019611000	1.638973000
C	-2.733145000	1.411155000	1.611562000
H	-3.092888000	1.372574000	2.630221000
C	-3.295849000	2.203547000	0.543080000
H	-4.144604000	2.867764000	0.623424000
C	-2.533586000	1.946266000	-0.647297000
H	-2.697211000	2.388078000	-1.620986000
C	-3.596237000	-1.614153000	-1.139426000
H	-2.847517000	-1.995652000	-1.819952000
C	-4.638667000	-0.670514000	-1.466504000

H	-4.816605000	-0.231280000	-2.438275000
C	-5.401756000	-0.416629000	-0.267380000
H	-6.247104000	0.251974000	-0.181290000
C	-4.828746000	-1.200914000	0.800281000
H	-5.169904000	-1.222907000	1.825945000
C	-3.711895000	-1.941053000	0.260971000
H	-3.070672000	-2.615834000	0.810607000
C	1.268161000	-0.433595000	-0.055058000
C	1.577536000	0.453909000	1.049827000
H	1.246909000	1.477516000	1.148434000
C	2.388143000	-0.275695000	1.994550000
H	2.775160000	0.110975000	2.927152000
C	2.603340000	-1.604291000	1.471072000
H	3.171025000	-2.391517000	1.946831000
C	1.941123000	-1.692383000	0.193127000
H	1.907955000	-2.548683000	-0.464969000
C	4.145261000	0.667126000	-1.655456000
H	3.535721000	0.894695000	-2.518722000
C	4.790894000	-0.594966000	-1.385500000
H	4.762705000	-1.469844000	-2.020135000
C	5.469018000	-0.487459000	-0.114998000
H	6.038917000	-1.268288000	0.369586000
C	5.240583000	0.841527000	0.401169000
H	5.609998000	1.231027000	1.339847000
C	4.422109000	1.554599000	-0.551433000
H	4.071656000	2.572617000	-0.451764000
N	0.564434000	-0.176634000	-1.252173000
H	-0.914968000	0.631316000	-2.366094000

TS(***E*-2** → ***Z*-2**)

Fe	-3.676553000	0.039408000	0.165068000
Fe	3.622431000	0.041301000	-0.040415000
C	-0.371644000	-0.630342000	-0.381909000
C	-1.456470000	-0.611209000	0.447299000
C	-2.476462000	-1.655079000	0.598269000
H	-2.461654000	-2.605819000	0.083705000
C	-3.351431000	-1.298006000	1.703758000
H	-4.112664000	-1.936858000	2.129678000
C	-3.014470000	0.035672000	2.119282000
H	-3.471869000	0.600612000	2.919699000
C	-1.927556000	0.504683000	1.273918000
H	-1.431151000	1.461930000	1.354138000
C	-3.990865000	0.715317000	-1.829582000
H	-3.242819000	0.743495000	-2.610342000
C	-4.292996000	1.777224000	-0.897449000
H	-3.797887000	2.737864000	-0.847514000
C	-5.369394000	1.337742000	-0.043090000
H	-5.821782000	1.910241000	0.755386000
C	-5.732094000	-0.002622000	-0.439887000
H	-6.503998000	-0.613035000	0.009148000
C	-4.878525000	-0.387288000	-1.537290000

H	-4.900272000	-1.338104000	-2.052838000
C	1.997429000	-1.139013000	-0.687868000
C	2.374369000	-1.537042000	0.641394000
H	1.733297000	-1.515329000	1.510205000
C	3.742594000	-1.985769000	0.576376000
H	4.326075000	-2.349208000	1.410269000
C	4.200374000	-1.856683000	-0.786724000
H	5.184125000	-2.110319000	-1.155250000
C	3.116730000	-1.326276000	-1.577155000
H	3.132934000	-1.129673000	-2.640554000
C	3.073429000	2.088497000	-0.033829000
H	2.109294000	2.478761000	-0.329687000
C	4.211766000	1.887804000	-0.899467000
H	4.251520000	2.102329000	-1.958433000
C	5.288904000	1.350938000	-0.102612000
H	6.273681000	1.085487000	-0.461259000
C	4.816240000	1.218873000	1.254769000
H	5.384343000	0.836256000	2.091201000
C	3.447155000	1.675114000	1.297356000
H	2.811623000	1.697062000	2.171595000
N	0.687556000	-0.678428000	-1.100466000
H	0.688954000	-0.380327000	-2.090236000

TS(***E*-3** → ***Z*-3**)

Fe	-3.526762000	0.047475000	-0.051280000
Fe	3.559105000	0.039501000	0.153673000
C	-0.600028000	-0.698678000	-1.070145000
C	-1.941970000	-1.187556000	-0.712778000
C	-3.063656000	-1.281135000	-1.628433000
H	-3.042472000	-1.027518000	-2.679719000
C	-4.192422000	-1.787513000	-0.896065000
H	-5.178768000	-1.973344000	-1.297248000
C	-3.779927000	-2.000523000	0.473312000
H	-4.407309000	-2.368421000	1.272989000
C	-2.396692000	-1.627947000	0.590260000
H	-1.788534000	-1.670809000	1.482350000
C	-2.881518000	2.066262000	0.061426000
H	-1.890563000	2.414106000	-0.195755000
C	-3.308077000	1.611811000	1.362670000
H	-2.695305000	1.564279000	2.251932000
C	-4.695863000	1.224177000	1.265546000
H	-5.303800000	0.832378000	2.069098000
C	-5.125280000	1.438802000	-0.095581000
H	-6.111301000	1.235988000	-0.490017000
C	-4.003202000	1.957923000	-0.840973000
H	-4.003234000	2.219243000	-1.890024000
C	1.462292000	-0.646536000	0.511599000
C	1.865749000	0.455894000	1.367143000
H	1.343650000	1.398670000	1.449897000
C	2.999079000	0.023978000	2.161149000
H	3.480867000	0.603924000	2.935902000

C	3.359992000	-1.303024000	1.735632000
H	4.164115000	-1.908026000	2.130620000
C	2.451512000	-1.696838000	0.677050000
H	2.441855000	-2.643057000	0.154933000
C	3.752459000	0.724411000	-1.846539000
H	2.962631000	0.739826000	-2.584980000
C	4.673245000	-0.362783000	-1.607038000
H	4.685404000	-1.310450000	-2.128059000
C	5.570482000	0.031878000	-0.548020000
H	6.371922000	-0.567170000	-0.137682000
C	5.204085000	1.364331000	-0.127695000
H	5.682783000	1.939775000	0.652843000
C	4.081214000	1.789897000	-0.927946000
H	3.572096000	2.740990000	-0.850495000
N	0.400587000	-0.673979000	-0.303037000
H	-0.517911000	-0.344878000	-2.114984000

TS(***E*-2** → ***E*-3**)

Fe	3.462730000	0.179715000	0.020682000
Fe	-3.476510000	-0.153535000	0.032591000
C	0.429537000	0.018354000	-1.030260000
C	1.566011000	0.852996000	-0.634424000
C	2.597945000	1.280676000	-1.567408000
H	2.629312000	1.011958000	-2.613772000
C	3.526035000	2.116281000	-0.863516000
H	4.403350000	2.588618000	-1.282766000
C	3.085575000	2.217828000	0.512898000
H	3.581979000	2.778436000	1.293053000
C	1.888967000	1.441762000	0.661838000
H	1.307240000	1.311893000	1.562350000
C	3.512105000	-1.942535000	0.148304000
H	2.696931000	-2.605554000	-0.106946000
C	3.760359000	-1.363861000	1.446309000
H	3.164264000	-1.515705000	2.335134000
C	4.945600000	-0.544346000	1.346337000
H	5.389190000	0.031437000	2.146692000
C	5.426040000	-0.617168000	-0.012682000
H	6.291388000	-0.103744000	-0.408337000
C	4.537810000	-1.479285000	-0.755111000
H	4.623443000	-1.732637000	-1.802412000
C	-1.588229000	-1.054254000	-0.191664000
C	-2.324237000	-1.148535000	-1.438362000
H	-2.014786000	-0.718879000	-2.378587000
C	-3.520740000	-1.901992000	-1.175422000
H	-4.290970000	-2.137603000	-1.896710000
C	-3.531798000	-2.270476000	0.223117000
H	-4.306796000	-2.832816000	0.724580000
C	-2.342562000	-1.741400000	0.834816000
H	-2.043030000	-1.836354000	1.868528000
C	-3.239534000	1.885051000	0.569429000
H	-2.284792000	2.371802000	0.711132000

C	-3.998044000	1.194022000	1.584328000
H	-3.717832000	1.085418000	2.622755000
C	-5.193175000	0.668778000	0.966990000
H	-5.964125000	0.093979000	1.461325000
C	-5.170938000	1.033286000	-0.429554000
H	-5.922052000	0.779179000	-1.164598000
C	-3.962264000	1.784718000	-0.675368000
H	-3.649155000	2.193471000	-1.625885000
N	-0.346937000	-0.400044000	0.076739000
H	0.466520000	-1.222683000	-0.491375000

TS(***Z*-2** → ***Z*-3**)

Fe	3.425413000	0.156490000	0.052268000
Fe	-3.428190000	-0.180324000	0.111108000
C	0.615652000	0.555833000	-1.277003000
C	1.509151000	1.017412000	-0.218978000
C	2.552722000	2.001850000	-0.467115000
H	2.699402000	2.515164000	-1.406582000
C	3.315058000	2.171167000	0.736863000
H	4.153409000	2.840168000	0.870736000
C	2.775685000	1.278310000	1.738429000
H	3.148111000	1.160121000	2.746699000
C	1.671556000	0.564075000	1.160306000
H	1.072159000	-0.185078000	1.654882000
C	3.664131000	-1.483102000	-1.274490000
H	2.908590000	-1.804936000	-1.977305000
C	3.798488000	-1.930965000	0.090375000
H	3.175664000	-2.664781000	0.582567000
C	4.907128000	-1.220788000	0.684621000
H	5.259263000	-1.327628000	1.701265000
C	5.454836000	-0.333707000	-0.313609000
H	6.288103000	0.341238000	-0.174744000
C	4.684610000	-0.493959000	-1.524537000
H	4.839403000	0.037213000	-2.453149000
C	-1.343886000	-0.599750000	-0.082849000
C	-2.114524000	-1.822293000	-0.114810000
H	-2.120640000	-2.532581000	-0.929233000
C	-2.826584000	-1.931608000	1.133752000
H	-3.480385000	-2.742919000	1.421322000
C	-2.521240000	-0.764884000	1.927959000
H	-2.911604000	-0.545817000	2.912109000
C	-1.617072000	0.068989000	1.174220000
H	-1.207000000	1.017534000	1.487688000
C	-4.040049000	1.084799000	-1.474788000
H	-3.385321000	1.456046000	-2.250928000
C	-4.300905000	1.724742000	-0.207728000
H	-3.882405000	2.663587000	0.127467000
C	-5.211375000	0.886734000	0.536471000
H	-5.593241000	1.086823000	1.528065000
C	-5.513390000	-0.270980000	-0.271485000
H	-6.160374000	-1.090552000	0.009423000

C	-4.788704000	-0.148763000	-1.514239000
H	-4.799949000	-0.859141000	-2.329198000
N	-0.534223000	-0.153998000	-1.162330000
H	-0.219146000	0.214526000	-2.280719000

W(CO)$_5$(*E*-2)

Fe	-0.203910000	3.472657000	0.068855000
O	-4.475485000	-3.391522000	-0.477936000
O	-4.057346000	0.120521000	2.101452000
Fe	3.645146000	-1.264184000	-0.002222000
W	-2.090487000	-1.299926000	-0.015994000
C	-0.495999000	0.233999000	0.293395000
C	-0.808154000	1.579135000	0.843561000
C	-1.968792000	2.409526000	0.602233000
H	-2.779379000	2.185092000	-0.073339000
O	-3.216525000	0.519450000	-2.424972000
C	-1.854085000	3.599052000	1.398629000
H	-2.567505000	4.410269000	1.426046000
O	-0.514426000	-3.072475000	-2.195591000
C	-0.624523000	3.523879000	2.149194000
H	-0.264121000	4.256904000	2.856763000
O	-0.950743000	-3.120813000	2.384366000
C	0.034988000	2.296901000	1.799040000
H	0.948825000	1.923040000	2.240329000
C	0.767815000	3.256639000	-1.808189000
H	0.927506000	2.322875000	-2.329750000
C	1.665489000	3.868925000	-0.855915000
H	2.629934000	3.488491000	-0.548035000
C	1.062834000	5.102080000	-0.407521000
H	1.483782000	5.790732000	0.311758000
C	-0.203988000	5.250131000	-1.082275000
H	-0.900924000	6.066263000	-0.952272000
C	-0.386588000	4.109447000	-1.947306000
H	-1.241866000	3.921038000	-2.580807000
C	1.569875000	-1.050514000	-0.330037000
C	1.822272000	-2.229905000	0.464222000
H	1.459400000	-2.414261000	1.463767000
C	2.644984000	-3.109256000	-0.324349000
H	3.003740000	-4.080686000	-0.015938000
C	2.912525000	-2.469075000	-1.588758000
H	3.498765000	-2.881115000	-2.397384000
C	2.256372000	-1.188517000	-1.593597000
H	2.238811000	-0.474963000	-2.405034000
C	4.412852000	0.426894000	1.019143000
H	3.851177000	1.295453000	1.337021000
C	5.091626000	0.268000000	-0.245954000
H	5.117116000	0.988253000	-1.052015000
C	5.737235000	-1.022574000	-0.244802000
H	6.317962000	-1.442827000	-1.054075000
C	5.458839000	-1.660201000	1.019652000
H	5.793590000	-2.642418000	1.322941000

C	4.641084000	-0.764848000	1.801810000
H	4.264741000	-0.954603000	2.797380000
C	-3.589924000	-2.617509000	-0.305010000
C	-3.301969000	-0.364731000	1.329308000
C	-2.799348000	-0.145008000	-1.536909000
C	-1.025910000	-2.379342000	-1.383487000
C	-1.361155000	-2.448243000	1.499981000
N	0.830383000	0.109745000	0.065692000
H	1.395723000	0.957872000	0.148946000

$W(CO)_5$(*Z*-2)

Fe	-1.187169000	3.071478000	0.040628000
O	-3.702808000	-4.185442000	-0.683437000
O	-3.304662000	-1.375958000	2.807263000
Fe	4.084240000	-0.187097000	0.036842000
W	-1.796751000	-1.699647000	-0.023762000
C	-0.362912000	0.008826000	0.466968000
C	-0.719508000	1.287440000	1.093509000
C	-2.097824000	1.696117000	1.361175000
H	-2.978333000	1.116425000	1.141264000
O	-3.933374000	0.182986000	-1.527321000
C	-2.093902000	2.985938000	1.973684000
H	-2.967852000	3.541190000	2.282899000
O	-0.333604000	-2.121910000	-2.858981000
C	-0.723031000	3.420244000	2.093717000
H	-0.388187000	4.358579000	2.513139000
O	0.228801000	-3.761696000	1.396105000
C	0.123072000	2.404798000	1.548352000
H	1.196274000	2.457463000	1.517115000
C	-0.958796000	2.826890000	-2.057951000
H	-0.658931000	1.908945000	-2.544392000
C	-0.075380000	3.864007000	-1.581674000
H	1.002906000	3.868859000	-1.653377000
C	-0.887914000	4.897905000	-0.985586000
H	-0.523815000	5.806913000	-0.527158000
C	-2.269869000	4.496546000	-1.092673000
H	-3.122235000	5.052201000	-0.727128000
C	-2.314577000	3.215428000	-1.754886000
H	-3.202946000	2.641484000	-1.975797000
C	2.142018000	0.502624000	0.447686000
C	2.784104000	1.394055000	-0.490326000
H	2.437508000	1.612397000	-1.489973000
C	3.954505000	1.928817000	0.157502000
H	4.657156000	2.624256000	-0.279239000
C	4.034386000	1.365899000	1.485387000
H	4.806025000	1.568424000	2.214384000
C	2.915970000	0.476691000	1.667377000
H	2.675724000	-0.094478000	2.552733000
C	4.008513000	-2.134956000	-0.797650000
H	3.120920000	-2.719144000	-1.001159000
C	4.773772000	-2.155168000	0.426081000

H	4.544866000	-2.735375000	1.309105000
C	5.895825000	-1.262577000	0.263341000
H	6.651914000	-1.051070000	1.006688000
C	5.824777000	-0.692067000	-1.060654000
H	6.517997000	0.022141000	-1.482988000
C	4.658067000	-1.230942000	-1.717011000
H	4.328935000	-1.000615000	-2.720710000
C	-2.998589000	-3.259044000	-0.437287000
C	-2.743488000	-1.471912000	1.770013000
C	-3.131862000	-0.479495000	-0.958759000
C	-0.865338000	-1.955794000	-1.811068000
C	-0.506582000	-2.987975000	0.879780000
N	0.942188000	-0.256596000	0.204254000
H	1.114987000	-1.160090000	-0.224342000

W(CO)$_5$(*E*-3)

Fe	-3.559728000	-0.902513000	0.049806000
O	3.659192000	-4.111790000	-0.366662000
O	0.714274000	-3.064155000	2.650432000
Fe	0.597950000	3.559970000	-0.047342000
W	1.778338000	-1.580190000	0.000132000
C	-0.806350000	0.435988000	0.672582000
C	-1.800295000	-0.504988000	1.154750000
C	-1.984561000	-1.940038000	1.001944000
H	-1.323328000	-2.625992000	0.504591000
O	-0.259647000	-2.999641000	-2.047875000
C	-3.223292000	-2.302101000	1.625566000
H	-3.632065000	-3.301326000	1.673205000
O	3.276482000	-0.313735000	-2.556530000
C	-3.834709000	-1.109299000	2.160237000
H	-4.777566000	-1.059726000	2.685626000
O	4.071189000	-0.340452000	1.891824000
C	-2.979712000	-0.001597000	1.860185000
H	-3.148680000	1.029474000	2.138900000
C	-3.463220000	-0.004195000	-1.871772000
H	-2.642911000	0.601040000	-2.232594000
C	-4.623836000	0.477536000	-1.161083000
H	-4.835468000	1.507056000	-0.908047000
C	-5.459157000	-0.659650000	-0.855766000
H	-6.398448000	-0.633136000	-0.321034000
C	-4.812578000	-1.840658000	-1.375269000
H	-5.181499000	-2.853678000	-1.295426000
C	-3.576729000	-1.436485000	-2.002402000
H	-2.857946000	-2.091523000	-2.474124000
C	1.055992000	1.467800000	-0.281499000
C	2.173919000	2.176818000	0.289020000
H	2.636043000	1.975129000	1.243457000
C	2.579363000	3.187843000	-0.655902000
H	3.396059000	3.882715000	-0.522488000
C	1.696879000	3.121688000	-1.794470000
H	1.740323000	3.750258000	-2.672324000

C	0.744467000	2.066934000	-1.558449000
H	-0.032233000	1.735729000	-2.233466000
C	-0.813952000	3.997275000	1.496402000
H	-1.202500000	3.309799000	2.234859000
C	-1.390392000	4.289780000	0.204512000
H	-2.288171000	3.852416000	-0.210248000
C	-0.568356000	5.289197000	-0.432836000
H	-0.731319000	5.715710000	-1.412809000
C	0.515041000	5.613176000	0.463931000
H	1.309716000	6.320366000	0.270979000
C	0.364128000	4.813199000	1.654360000
H	1.020320000	4.821164000	2.513515000
C	2.958856000	-3.157101000	-0.229270000
C	1.040543000	-2.486762000	1.669551000
C	0.456697000	-2.458218000	-1.273473000
C	2.692208000	-0.740678000	-1.621648000
C	3.203228000	-0.759670000	1.204816000
N	0.421995000	0.265282000	0.229382000
H	-1.152045000	1.471141000	0.704337000

$W(CO)_5$(*Z*-3)

Fe	-4.176630000	0.474238000	0.065461000
O	3.138082000	-4.350525000	-0.491399000
O	-0.078806000	-3.621818000	2.321054000
Fe	2.119530000	2.565509000	-0.140203000
W	1.293686000	-1.818911000	0.038049000
C	-1.241600000	-0.181488000	0.948497000
C	-2.368238000	0.714962000	1.150565000
C	-3.463962000	0.338379000	2.040388000
H	-3.487073000	-0.544317000	2.664696000
O	-0.909984000	-2.905469000	-2.037545000
C	-4.461775000	1.363941000	1.981116000
H	-5.387721000	1.381696000	2.537838000
O	2.885281000	-0.559208000	-2.459454000
C	-4.020437000	2.364085000	1.036104000
H	-4.566533000	3.253268000	0.754538000
O	3.315192000	-0.780781000	2.322708000
C	-2.742691000	1.971157000	0.517569000
H	-2.164849000	2.517745000	-0.209398000
C	-3.997139000	-1.000260000	-1.464604000
H	-3.109142000	-1.574765000	-1.688384000
C	-4.349736000	0.273150000	-2.042205000
H	-3.772820000	0.820316000	-2.774492000
C	-5.611880000	0.684873000	-1.474470000
H	-6.141279000	1.600518000	-1.698515000
C	-6.035864000	-0.333356000	-0.543942000
H	-6.938493000	-0.313250000	0.050980000
C	-5.035682000	-1.374043000	-0.534953000
H	-5.064883000	-2.278820000	0.055943000
C	0.299612000	1.374126000	0.067053000
C	0.424339000	1.896355000	-1.272198000

H	0.432756000	1.312352000	-2.180986000
C	0.459276000	3.334953000	-1.179861000
H	0.542148000	4.017770000	-2.013554000
C	0.394330000	3.698922000	0.216662000
H	0.422326000	4.702664000	0.616070000
C	0.319067000	2.485695000	0.988767000
H	0.232860000	2.409298000	2.063310000
C	4.010291000	1.553993000	-0.018570000
H	4.180543000	0.490663000	0.022671000
C	3.839418000	2.427982000	1.114394000
H	3.839423000	2.128285000	2.152665000
C	3.681565000	3.772003000	0.614803000
H	3.522243000	4.658357000	1.213215000
C	3.748575000	3.722936000	-0.827025000
H	3.646055000	4.565524000	-1.497013000
C	3.946466000	2.348905000	-1.217761000
H	4.031127000	1.976955000	-2.228701000
C	2.445050000	-3.401520000	-0.292704000
C	0.400551000	-2.915855000	1.497100000
C	-0.088804000	-2.499459000	-1.285197000
C	2.282816000	-0.943159000	-1.515611000
C	2.582472000	-1.130027000	1.462749000
N	-0.046133000	0.013770000	0.422178000
H	-1.436187000	-1.195704000	1.288622000

[W(CO)$_5$···(*E*-3)]

Fe	-4.975956000	-0.662836000	0.031431000
O	3.411457000	-4.493421000	0.233206000
O	1.714443000	-1.586899000	3.164277000
Fe	1.141711000	3.071266000	-0.211216000
W	2.662991000	-1.438743000	0.091932000
C	-2.247365000	0.814923000	0.670461000
C	-3.694813000	0.830936000	0.813695000
C	-4.433517000	0.187426000	1.886842000
H	-3.998524000	-0.347626000	2.720193000
O	-0.308909000	-2.231766000	-0.858150000
C	-5.834596000	0.408114000	1.655767000
H	-6.643669000	0.060665000	2.282561000
O	3.652876000	-1.400272000	-2.973401000
C	-5.973024000	1.174852000	0.437409000
H	-6.905826000	1.492752000	-0.006777000
O	5.659325000	-0.745430000	1.044947000
C	-4.659937000	1.431431000	-0.086057000
H	-4.412983000	1.978697000	-0.983508000
C	-3.840195000	-2.133654000	-1.006651000
H	-2.763557000	-2.129601000	-1.102572000
C	-4.774669000	-1.501780000	-1.905515000
H	-4.519966000	-0.946641000	-2.797469000
C	-6.108113000	-1.733638000	-1.400215000
H	-7.027856000	-1.381289000	-1.846185000
C	-5.996260000	-2.506269000	-0.186207000

H	-6.817551000	-2.834723000	0.435766000
C	-4.594295000	-2.750502000	0.058454000
H	-4.181152000	-3.300868000	0.892291000
C	-0.221927000	1.419016000	-0.361520000
C	0.760358000	1.208830000	0.679770000
H	0.563787000	1.074248000	1.733533000
C	2.081483000	1.178323000	0.053905000
H	3.018012000	1.295797000	0.588433000
C	1.892764000	1.479919000	-1.365831000
H	2.680114000	1.567660000	-2.099914000
C	0.488103000	1.612540000	-1.609289000
H	0.009672000	1.793486000	-2.560111000
C	0.033261000	4.816236000	0.226300000
H	-1.041267000	4.834859000	0.342879000
C	0.752721000	4.986886000	-1.013168000
H	0.309726000	5.167651000	-1.982555000
C	2.163305000	4.856359000	-0.733786000
H	2.964647000	4.931160000	-1.455672000
C	2.313323000	4.605633000	0.679309000
H	3.248243000	4.448741000	1.199995000
C	0.998388000	4.578676000	1.273295000
H	0.775369000	4.409178000	2.317407000
C	3.128302000	-3.336858000	0.178193000
C	2.059558000	-1.510597000	2.034944000
C	0.778917000	-1.922552000	-0.515940000
C	3.281336000	-1.389832000	-1.851533000
C	4.552879000	-0.974216000	0.695000000
N	-1.627716000	1.457055000	-0.279290000
H	-1.703612000	0.215382000	1.415365000

TS(**W(CO)$_5$(*E*-2)** → **W(CO)$_5$(*Z*-2)**)

Fe	-0.836534000	3.143062000	0.131118000
O	-4.249058000	-3.701932000	-0.252514000
O	-2.332835000	-1.330563000	3.183581000
Fe	3.978940000	-0.644243000	-0.049567000
W	-1.918853000	-1.485198000	-0.008814000
C	-0.370389000	0.036986000	0.122469000
C	-0.338272000	1.233547000	0.919985000
C	-1.425334000	1.842050000	1.696743000
H	-2.414967000	1.436623000	1.834280000
O	-4.220719000	0.719096000	-0.445430000
C	-0.943444000	3.054931000	2.271310000
H	-1.511802000	3.725638000	2.900285000
O	-1.381290000	-1.620935000	-3.182047000
C	0.431750000	3.247538000	1.856674000
H	1.060562000	4.082245000	2.133012000
O	0.080906000	-3.999765000	0.355034000
C	0.804899000	2.159603000	1.015589000
H	1.765228000	1.982135000	0.559608000
C	-1.469728000	3.083829000	-1.903431000
H	-1.543949000	2.186480000	-2.502623000

C	-0.301976000	3.921313000	-1.772095000
H	0.645895000	3.778111000	-2.271195000
C	-0.632979000	4.998431000	-0.869325000
H	0.031372000	5.791857000	-0.556147000
C	-1.998454000	4.818761000	-0.440284000
H	-2.534871000	5.454555000	0.250189000
C	-2.516603000	3.631701000	-1.077682000
H	-3.506987000	3.218735000	-0.953316000
C	1.846581000	-1.019341000	-0.371541000
C	2.264349000	-1.375614000	0.966765000
H	1.852381000	-0.977000000	1.883046000
C	3.277121000	-2.401320000	0.860494000
H	3.755555000	-2.904401000	1.688670000
C	3.531759000	-2.631429000	-0.538754000
H	4.239849000	-3.335389000	-0.952522000
C	2.666297000	-1.759047000	-1.301362000
H	2.602483000	-1.706378000	-2.379735000
C	4.453038000	1.393867000	-0.420598000
H	3.761308000	2.149802000	-0.766893000
C	5.258315000	0.539311000	-1.260850000
H	5.268277000	0.538423000	-2.342151000
C	6.047397000	-0.314071000	-0.406294000
H	6.746590000	-1.070507000	-0.735075000
C	5.727225000	0.008783000	0.964261000
H	6.147696000	-0.460502000	1.843010000
C	4.741759000	1.063261000	0.955440000
H	4.297205000	1.519733000	1.829259000
C	-3.384829000	-2.894642000	-0.166950000
C	-2.181549000	-1.388407000	2.012391000
C	-3.356764000	-0.074991000	-0.279810000
C	-1.584957000	-1.568565000	-2.016764000
C	-0.592384000	-3.039363000	0.230931000
N	0.855897000	-0.079225000	-0.687466000
H	0.746445000	0.149803000	-1.670418000

TS(**W(CO)$_5$(*E*-2) → [W(CO)$_5$···(*E*-3)]**)

Fe	-2.830114000	-2.312847000	-0.166930000
O	-0.517992000	5.399615000	-0.008177000
O	-1.973837000	2.377282000	-2.819785000
Fe	3.544013000	-1.371790000	0.120343000
W	-0.464356000	2.205082000	0.021424000
C	-0.428045000	-0.090284000	-0.049438000
C	-1.458744000	-0.822293000	-0.847371000
C	-2.828578000	-0.428357000	-1.126817000
H	-3.330127000	0.452192000	-0.759286000
O	-3.301185000	2.190621000	1.549158000
C	-3.416133000	-1.410651000	-1.993606000
H	-4.424483000	-1.386834000	-2.381738000
O	0.910720000	2.501573000	2.918632000
C	-2.428820000	-2.432772000	-2.250000000
H	-2.566610000	-3.306845000	-2.870816000

O	2.254017000	2.317720000	-1.701492000
C	-1.233602000	-2.085878000	-1.534381000
H	-0.306920000	-2.638700000	-1.515008000
C	-2.719563000	-2.560037000	1.940963000
H	-2.097568000	-1.991796000	2.619471000
C	-2.350701000	-3.789082000	1.279960000
H	-1.400607000	-4.297416000	1.363850000
C	-3.477122000	-4.220286000	0.486718000
H	-3.515131000	-5.103031000	-0.136441000
C	-4.538739000	-3.257234000	0.656589000
H	-5.509539000	-3.291694000	0.182032000
C	-4.070219000	-2.229497000	1.554993000
H	-4.630234000	-1.363661000	1.879428000
C	1.741706000	-0.852331000	1.070498000
C	2.701090000	0.249077000	1.172887000
H	2.599726000	1.243356000	0.779947000
C	3.835009000	-0.229797000	1.902587000
H	4.716386000	0.349983000	2.137999000
C	3.616350000	-1.615349000	2.252732000
H	4.304301000	-2.244474000	2.799152000
C	2.348705000	-2.011283000	1.730016000
H	1.873733000	-2.977259000	1.818794000
C	3.239663000	-1.791785000	-1.941996000
H	2.282938000	-1.752206000	-2.444155000
C	3.802789000	-2.943702000	-1.279928000
H	3.352330000	-3.924005000	-1.213731000
C	5.080169000	-2.556223000	-0.729505000
H	5.748540000	-3.192185000	-0.165741000
C	5.299714000	-1.165485000	-1.046227000
H	6.160893000	-0.577541000	-0.760679000
C	4.159594000	-0.690838000	-1.794171000
H	4.011457000	0.313592000	-2.164308000
C	-0.503265000	4.210277000	0.001872000
C	-1.423590000	2.264095000	-1.779253000
C	-2.254585000	2.176430000	0.994061000
C	0.434959000	2.329399000	1.846488000
C	1.271172000	2.255129000	-1.041949000
N	0.525477000	-1.038194000	0.419728000
H	-0.515494000	-0.521561000	1.158028000

TS(**W(CO)$_5$(*Z*-2) → W(CO)$_5$(*Z*-3)**)

Fe	3.816730000	-1.134316000	0.084911000
O	-0.903993000	5.030967000	-0.683250000
O	1.906773000	2.855988000	1.827147000
Fe	-2.887945000	-2.137365000	-0.146567000
W	-0.622853000	1.959126000	0.029742000
C	1.013016000	-0.454623000	1.190129000
C	1.927400000	-1.538699000	0.968556000
C	3.021093000	-1.801763000	1.910026000
H	3.143062000	-1.303975000	2.861036000
O	1.197876000	1.550640000	-2.595427000

C	3.834675000	-2.843927000	1.369546000
H	4.709771000	-3.273718000	1.835750000
O	-3.279844000	1.648421000	-1.744396000
C	3.302663000	-3.209955000	0.072707000
H	3.722906000	-3.951424000	-0.592548000
O	-2.538633000	2.371861000	2.583727000
C	2.144834000	-2.414629000	-0.188603000
H	1.530600000	-2.461877000	-1.074403000
C	4.067477000	0.898437000	-0.516257000
H	3.342781000	1.685759000	-0.376094000
C	4.166288000	0.029975000	-1.662739000
H	3.532081000	0.062246000	-2.536771000
C	5.267353000	-0.875359000	-1.434858000
H	5.596487000	-1.657570000	-2.104738000
C	5.840854000	-0.567390000	-0.146817000
H	6.673294000	-1.079490000	0.315646000
C	5.094322000	0.528851000	0.424339000
H	5.272681000	0.993214000	1.383725000
C	-0.870506000	-1.459477000	-0.150994000
C	-1.377933000	-1.494485000	-1.503389000
H	-1.433307000	-0.660966000	-2.185662000
C	-1.757665000	-2.853229000	-1.793809000
H	-2.176849000	-3.203739000	-2.726365000
C	-1.498867000	-3.656238000	-0.622828000
H	-1.696635000	-4.713794000	-0.520063000
C	-0.962179000	-2.797266000	0.398318000
H	-0.651255000	-3.096601000	1.388785000
C	-4.186257000	-0.830588000	0.916829000
H	-3.914147000	0.119871000	1.352131000
C	-4.123019000	-2.111748000	1.577124000
H	-3.789664000	-2.290584000	2.590003000
C	-4.588172000	-3.113846000	0.647966000
H	-4.650888000	-4.176476000	0.837752000
C	-4.937932000	-2.449853000	-0.585015000
H	-5.307747000	-2.928484000	-1.481273000
C	-4.688239000	-1.039308000	-0.419689000
H	-4.837302000	-0.273526000	-1.166657000
C	-0.801769000	3.873707000	-0.414423000
C	1.000025000	2.458867000	1.181369000
C	0.538219000	1.688906000	-1.621691000
C	-2.294052000	1.680991000	-1.085528000
C	-1.822301000	2.201742000	1.656168000
N	-0.265494000	-0.331003000	0.535429000
H	-0.178091000	-0.141062000	1.752804000

$W(CO)_4$(*E*-2)

Fe	3.449042000	-1.518441000	0.074666000
O	-1.241340000	4.555656000	-0.302089000
O	-0.228459000	2.012366000	3.313698000
Fe	-3.748834000	-1.125990000	-0.254628000
W	0.052120000	1.667858000	0.131141000

C	0.412957000	-0.513165000	0.325259000
C	1.538771000	-1.216161000	0.942244000
C	2.569804000	-0.590034000	1.756088000
H	2.623991000	0.460315000	1.993187000
O	2.936011000	2.859191000	0.453436000
C	3.474002000	-1.604435000	2.206744000
H	4.339546000	-1.445578000	2.833816000
O	0.676154000	1.496705000	-3.020870000
C	3.038928000	-2.871925000	1.667982000
H	3.524812000	-3.825755000	1.818881000
C	1.860779000	-2.645019000	0.882768000
H	1.320587000	-3.418874000	0.353141000
C	3.544117000	-0.741272000	-1.905345000
H	2.725816000	-0.277814000	-2.437423000
C	3.872300000	-2.145441000	-1.911192000
H	3.348091000	-2.919768000	-2.454041000
C	5.030946000	-2.334404000	-1.069582000
H	5.520933000	-3.275782000	-0.862404000
C	5.414914000	-1.045153000	-0.546073000
H	6.237238000	-0.853418000	0.129112000
C	4.493263000	-0.060185000	-1.058359000
H	4.498910000	0.996828000	-0.833141000
C	-1.725027000	-0.816079000	-0.764127000
C	-2.329715000	0.441409000	-0.371172000
H	-2.161554000	0.971440000	0.570756000
C	-3.414734000	0.704123000	-1.286872000
H	-4.069233000	1.563033000	-1.257742000
C	-3.473605000	-0.381404000	-2.228441000
H	-4.177858000	-0.477406000	-3.042119000
C	-2.429679000	-1.325680000	-1.909083000
H	-2.189447000	-2.226136000	-2.456078000
C	-3.908939000	-2.514527000	1.339394000
H	-3.084252000	-3.002924000	1.839986000
C	-4.616464000	-3.008165000	0.181372000
H	-4.410685000	-3.929102000	-0.346476000
C	-5.648238000	-2.055289000	-0.150859000
H	-6.341895000	-2.131989000	-0.976510000
C	-5.579232000	-0.972717000	0.801853000
H	-6.214719000	-0.098308000	0.817341000
C	-4.505387000	-1.257263000	1.722465000
H	-4.195809000	-0.633727000	2.549710000
C	-0.727715000	3.490506000	-0.135045000
C	-0.164392000	1.860479000	2.135699000
C	1.824461000	2.416691000	0.332794000
C	0.411158000	1.543366000	-1.864110000
N	-0.545981000	-1.333537000	-0.177161000
H	-0.453450000	-2.347442000	-0.145165000

$W(CO)_4$(*Z*-2)

Fe	-2.299545000	2.395583000	0.136685000
O	0.525451000	-4.083424000	-0.681455000

O	-3.962459000	-3.710694000	0.082945000
Fe	4.206437000	0.513939000	-0.116315000
W	-1.432374000	-1.757512000	0.058007000
C	-0.154045000	0.031706000	0.221887000
C	-0.653100000	1.247786000	0.875458000
C	-1.769484000	1.235311000	1.819813000
H	-2.269150000	0.356246000	2.201596000
O	-1.766326000	-1.361201000	-3.111147000
C	-2.025770000	2.579624000	2.240394000
H	-2.776873000	2.886935000	2.953678000
C	-1.105853000	3.448896000	1.545708000
H	-1.061057000	4.525018000	1.636615000
O	-0.828331000	-2.529676000	3.123319000
C	-0.270882000	2.645229000	0.702358000
H	0.508739000	3.017042000	0.056908000
C	-2.840384000	1.920141000	-1.873005000
H	-2.306313000	1.252257000	-2.532748000
C	-2.612892000	3.335653000	-1.742716000
H	-1.872071000	3.912162000	-2.278831000
C	-3.542880000	3.848327000	-0.764069000
H	-3.616839000	4.875051000	-0.433672000
C	-4.345076000	2.746052000	-0.291228000
H	-5.127928000	2.804087000	0.452213000
C	-3.909737000	1.552038000	-0.973748000
H	-4.330344000	0.563062000	-0.854441000
C	2.156250000	0.982232000	-0.156254000
C	2.823051000	1.635575000	-1.258970000
H	2.600545000	1.494199000	-2.307234000
C	3.815405000	2.517331000	-0.697873000
H	4.488910000	3.148974000	-1.259491000
C	3.757675000	2.406701000	0.740668000
H	4.382180000	2.939895000	1.443246000
C	2.736672000	1.450138000	1.080716000
H	2.438614000	1.144216000	2.073062000
C	4.477894000	-1.584321000	-0.280571000
H	3.697455000	-2.326740000	-0.379558000
C	5.040989000	-1.109358000	0.960560000
H	4.743769000	-1.420556000	1.952247000
C	6.067826000	-0.145447000	0.644005000
H	6.669010000	0.399712000	1.358273000
C	6.140251000	-0.025530000	-0.792703000
H	6.806481000	0.623875000	-1.343476000
C	5.157742000	-0.914796000	-1.364203000
H	4.966532000	-1.056987000	-2.418862000
C	-0.254281000	-3.212293000	-0.395193000
C	-2.996415000	-3.010088000	0.062849000
C	-1.670919000	-1.467642000	-1.930034000
C	-1.075891000	-2.203127000	2.008563000
N	1.098869000	0.017678000	-0.282035000
H	1.359176000	-0.834789000	-0.771174000

W(CO)$_4$(κ*C*, κ*N*-2)

Fe	3.335715000	-1.208307000	0.165776000
O	-3.189688000	2.465260000	-1.430591000
O	1.224264000	4.755301000	-0.650824000
Fe	-2.775671000	-2.082185000	0.043360000
W	-0.323671000	2.056499000	-0.053370000
C	0.789450000	0.632960000	0.875072000
C	1.992027000	0.001573000	1.304396000
C	3.304590000	0.640516000	1.203227000
H	3.478976000	1.618137000	0.777524000
O	0.809570000	1.314437000	-2.965282000
C	4.269131000	-0.217701000	1.812830000
H	5.326708000	-0.017053000	1.908876000
C	3.590344000	-1.412069000	2.273177000
H	4.058237000	-2.250643000	2.769563000
O	-1.325084000	3.652125000	2.567278000
C	2.202649000	-1.301222000	1.946016000
H	1.437763000	-2.030412000	2.162028000
C	2.662294000	-1.577401000	-1.824519000
H	1.743303000	-1.196421000	-2.243514000
C	2.814375000	-2.821295000	-1.111872000
H	2.032346000	-3.542318000	-0.920854000
C	4.196089000	-2.931570000	-0.706394000
H	4.631537000	-3.746314000	-0.144602000
C	4.890696000	-1.751347000	-1.161930000
H	5.936398000	-1.528638000	-1.000926000
C	3.939520000	-0.910474000	-1.850140000
H	4.146952000	0.046560000	-2.307168000
C	-0.843980000	-1.313532000	0.423505000
C	-0.980507000	-1.624608000	-0.977321000
H	-0.904387000	-0.920825000	-1.791947000
C	-1.253644000	-3.035589000	-1.086706000
H	-1.410271000	-3.577541000	-2.008407000
C	-1.303434000	-3.590848000	0.244148000
H	-1.503194000	-4.622667000	0.496386000
C	-1.052812000	-2.525694000	1.183378000
H	-1.022136000	-2.614956000	2.261113000
C	-4.241855000	-0.666804000	0.630867000
H	-4.045392000	0.298207000	1.076997000
C	-4.444566000	-1.908507000	1.339019000
H	-4.421392000	-2.040903000	2.411983000
C	-4.685586000	-2.944186000	0.362818000
H	-4.864952000	-3.989061000	0.575488000
C	-4.631292000	-2.341337000	-0.947983000
H	-4.763900000	-2.855794000	-1.889671000
C	-4.358578000	-0.933990000	-0.782206000
H	-4.254728000	-0.204173000	-1.572653000
C	-2.106267000	2.403855000	-0.939381000
C	0.610137000	3.748828000	-0.447498000
C	0.370067000	1.533964000	-1.883137000
C	-0.977843000	3.017224000	1.629000000

N	-0.553567000	-0.012118000	0.978155000
H	-0.994905000	0.110083000	1.889702000

***cis*(N,H)-W(CO)$_4$(H)(*Z*-15)**

Fe	-3.349652000	-1.610392000	-0.265060000
O	0.506285000	5.060692000	0.184112000
O	-2.909669000	2.686557000	-0.943414000
Fe	3.546422000	-1.425313000	0.255756000
W	0.027145000	1.891541000	0.070906000
C	-0.512549000	-0.176457000	-0.399595000
C	-1.397619000	-1.160277000	-0.968920000
C	-2.392564000	-0.855738000	-1.987825000
H	-2.551640000	0.115456000	-2.432309000
O	-1.129644000	1.834725000	3.070554000
C	-3.072475000	-2.070379000	-2.326622000
H	-3.856026000	-2.172756000	-3.063586000
C	-2.536528000	-3.133127000	-1.506672000
H	-2.859204000	-4.164677000	-1.517169000
O	1.588548000	2.056438000	-2.742558000
C	-1.513254000	-2.583446000	-0.665021000
H	-0.943109000	-3.123141000	0.075331000
C	-3.813502000	-0.535957000	1.512572000
H	-3.152280000	0.147867000	2.024497000
C	-3.911484000	-1.954055000	1.753947000
H	-3.337615000	-2.516366000	2.477197000
C	-4.911367000	-2.488751000	0.859858000
H	-5.212413000	-3.524760000	0.788939000
C	-5.428387000	-1.399212000	0.066755000
H	-6.182981000	-1.476970000	-0.703631000
C	-4.747265000	-0.191518000	0.467878000
H	-4.903593000	0.795219000	0.055425000
C	1.516049000	-1.081344000	0.815331000
C	2.404175000	-0.727943000	1.899619000
H	2.470678000	0.251618000	2.349851000
C	3.130112000	-1.910740000	2.275349000
H	3.865870000	-1.976371000	3.064104000
C	2.716028000	-2.989414000	1.409082000
H	3.093178000	-4.002227000	1.430239000
C	1.727907000	-2.480716000	0.492737000
H	1.243310000	-3.036800000	-0.294883000
C	4.066875000	-0.346627000	-1.502720000
H	3.400099000	0.269905000	-2.087110000
C	4.307384000	-1.755175000	-1.698347000
H	3.847426000	-2.379199000	-2.452010000
C	5.270053000	-2.184740000	-0.711877000
H	5.652240000	-3.189014000	-0.591201000
C	5.622543000	-1.040008000	0.094198000
H	6.313611000	-1.037318000	0.925784000
C	4.876537000	0.095396000	-0.393004000
H	4.913487000	1.099753000	0.004893000
C	0.296292000	3.896917000	0.140872000
C	-1.816487000	2.402037000	-0.596683000

C	-0.722437000	1.825104000	1.958722000
C	1.004196000	1.964821000	-1.717193000
N	0.593402000	-0.188942000	0.258942000
H	1.502846000	2.377455000	0.943687000

cis(C,H)-W(CO)$_4$(H)(_Z_-15)

Fe	3.621436000	-1.266892000	0.253256000
O	0.322706000	5.059298000	0.267325000
O	-2.972675000	2.562843000	-1.331958000
Fe	-3.240001000	-1.766123000	-0.218562000
W	-0.122511000	1.897891000	0.039082000
C	0.683428000	-0.053210000	0.465091000
C	1.711378000	-0.848038000	1.085534000
C	2.759437000	-0.290926000	1.925237000
H	2.859088000	0.757231000	2.166680000
O	-1.415143000	1.916883000	2.984684000
C	3.586318000	-1.367022000	2.382299000
H	4.444741000	-1.275477000	3.032283000
C	3.079751000	-2.598618000	1.818028000
H	3.499731000	-3.583130000	1.969161000
O	1.550851000	2.024457000	-2.706383000
C	1.935264000	-2.289687000	1.010455000
H	1.343371000	-2.996393000	0.449733000
C	3.799026000	-0.560008000	-1.748113000
H	3.015363000	-0.081536000	-2.316433000
C	4.067143000	-1.975222000	-1.699208000
H	3.520457000	-2.744551000	-2.226457000
C	5.195557000	-2.184085000	-0.822387000
H	5.638098000	-3.138250000	-0.571867000
C	5.621476000	-0.895876000	-0.329726000
H	6.436047000	-0.717266000	0.358439000
C	4.755255000	0.108581000	-0.898714000
H	4.811218000	1.173296000	-0.721599000
C	-1.183697000	-1.360420000	-0.618077000
C	-1.949077000	-1.355742000	-1.845003000
H	-2.021756000	-0.526746000	-2.534059000
C	-2.533391000	-2.661454000	-2.002099000
H	-3.153143000	-2.980865000	-2.827809000
C	-2.158425000	-3.464329000	-0.862397000
H	-2.456468000	-4.487193000	-0.679891000
C	-1.334051000	-2.661709000	0.004835000
H	-0.913386000	-2.966457000	0.951580000
C	-4.055669000	-0.340997000	1.129083000
H	-3.514015000	0.475334000	1.584004000
C	-4.194430000	-1.669088000	1.674581000
H	-3.773614000	-2.019256000	2.606896000
C	-4.993260000	-2.446353000	0.757108000
H	-5.270118000	-3.484655000	0.877184000
C	-5.347119000	-1.597125000	-0.355292000
H	-5.933971000	-1.888355000	-1.215525000
C	-4.765995000	-0.296102000	-0.126601000

H	-4.845592000	0.559968000	-0.781593000
C	0.135861000	3.892584000	0.175196000
C	-1.926846000	2.320802000	-0.844622000
C	-0.959963000	1.878508000	1.891737000
C	0.920235000	1.941655000	-1.706264000
N	-0.408408000	-0.279635000	-0.179696000
H	1.346129000	2.431616000	0.869658000

W(CO)$_4$(*E*-3)

Fe	-3.175663000	-1.809806000	-0.124014000
O	2.025454000	3.990056000	0.275299000
O	0.133125000	2.049137000	-3.185687000
Fe	3.555141000	-1.354505000	0.153313000
W	-0.346362000	1.962623000	-0.006281000
C	-0.051375000	-1.124722000	-0.303416000
C	-1.380803000	-0.971396000	-0.848115000
C	-2.318586000	0.089310000	-0.497741000
H	-2.269494000	0.741059000	0.381683000
O	-2.360445000	4.380791000	-0.344772000
C	-3.488066000	-0.063053000	-1.319865000
H	-4.368462000	0.562249000	-1.287060000
O	-0.679686000	2.188831000	3.180994000
C	-3.288277000	-1.206550000	-2.173889000
H	-3.990472000	-1.577517000	-2.906591000
C	-2.003366000	-1.778810000	-1.879219000
H	-1.551323000	-2.626498000	-2.374112000
C	-2.822200000	-3.011966000	1.589898000
H	-1.858723000	-3.180880000	2.050484000
C	-3.413661000	-3.807506000	0.540615000
H	-2.973157000	-4.678256000	0.075321000
C	-4.699758000	-3.235168000	0.221270000
H	-5.386567000	-3.596327000	-0.531500000
C	-4.901283000	-2.086195000	1.071420000
H	-5.765852000	-1.437227000	1.068567000
C	-3.740360000	-1.948036000	1.917114000
H	-3.584480000	-1.180483000	2.662369000
C	1.842052000	-0.315699000	0.856015000
C	2.983676000	0.565026000	0.870964000
H	3.081421000	1.489914000	0.325310000
C	3.972956000	-0.012678000	1.740633000
H	4.942870000	0.411464000	1.957180000
C	3.449389000	-1.249960000	2.267044000
H	3.951131000	-1.911182000	2.959103000
C	2.135049000	-1.447723000	1.717204000
H	1.458590000	-2.257821000	1.951705000
C	3.281972000	-1.941526000	-1.865217000
H	2.413786000	-1.714140000	-2.468710000
C	3.463242000	-3.108381000	-1.033487000
H	2.759258000	-3.919521000	-0.907259000
C	4.758515000	-3.008287000	-0.402877000
H	5.188056000	-3.723358000	0.284974000

C	5.375752000	-1.781239000	-0.845266000
H	6.346100000	-1.412947000	-0.542651000
C	4.463473000	-1.122013000	-1.748476000
H	4.630912000	-0.176222000	-2.244467000
C	1.112214000	3.217940000	0.169739000
C	-0.053559000	1.977709000	-2.016210000
C	-1.575026000	3.486845000	-0.214204000
C	-0.571530000	2.062395000	2.004476000
N	0.598151000	-0.086995000	0.180032000
H	0.423497000	-2.105768000	-0.328691000

W(CO)$_4$(*Z*-3)

Fe	-4.191313000	0.182615000	0.040480000
O	4.305805000	-2.782944000	-0.727582000
O	0.167109000	-4.224553000	0.258994000
Fe	2.021295000	2.249455000	-0.137765000
W	1.545669000	-1.425009000	0.054643000
C	-1.313729000	-0.476941000	1.040423000
C	-2.508853000	0.298574000	1.314501000
C	-3.643848000	-0.291522000	2.018181000
H	-3.661483000	-1.280106000	2.456118000
O	0.805790000	-1.427594000	-3.074150000
C	-4.699508000	0.675276000	2.054640000
H	-5.668187000	0.539667000	2.513776000
C	-4.247903000	1.859620000	1.360953000
H	-4.825031000	2.760243000	1.206421000
O	2.365799000	-1.750847000	3.148435000
C	-2.908621000	1.637048000	0.899416000
H	-2.308760000	2.342960000	0.348593000
C	-3.758762000	-0.994892000	-1.678430000
H	-2.813239000	-1.478547000	-1.881718000
C	-4.130266000	0.341345000	-2.074963000
H	-3.516089000	1.032545000	-2.634758000
C	-5.469714000	0.590671000	-1.596290000
H	-6.031827000	1.504870000	-1.726719000
C	-5.922542000	-0.590782000	-0.902273000
H	-6.882277000	-0.715574000	-0.420374000
C	-4.863444000	-1.570491000	-0.950115000
H	-4.893069000	-2.562062000	-0.520669000
C	0.133074000	1.269853000	0.260441000
C	0.131300000	1.867087000	-1.057384000
H	-0.045694000	1.345715000	-1.986385000
C	0.340103000	3.280892000	-0.890410000
H	0.384851000	4.010397000	-1.686490000
C	0.500656000	3.550991000	0.519311000
H	0.689146000	4.518249000	0.962928000
C	0.390945000	2.305349000	1.232885000
H	0.443517000	2.163259000	2.302687000
C	3.865248000	1.134683000	0.114052000
H	4.024478000	0.131907000	0.482081000
C	3.873197000	2.332531000	0.914572000

H	3.985393000	2.376593000	1.988719000
C	3.739121000	3.459752000	0.026207000
H	3.711652000	4.499900000	0.319975000
C	3.626513000	2.956248000	-1.322893000
H	3.502539000	3.554085000	-2.215117000
C	3.694515000	1.519319000	-1.269072000
H	3.660135000	0.847437000	-2.114686000
C	3.256515000	-2.286854000	-0.432416000
C	0.720125000	-3.159126000	0.174100000
C	1.068188000	-1.384482000	-1.914609000
C	2.057181000	-1.587877000	2.012671000
N	-0.150203000	-0.109613000	0.550381000
H	-1.396611000	-1.537529000	1.272200000

[W(CO)$_4$···(*E*-3)]

Fe	-4.501020000	0.222160000	0.111791000
O	3.989649000	-3.617938000	-1.025505000
O	3.138308000	-2.081093000	2.995121000
Fe	2.231285000	2.612829000	-0.261130000
W	1.737786000	-1.767780000	0.122778000
C	-1.408891000	0.694823000	0.637780000
C	-2.768314000	0.969533000	1.068500000
C	-3.530594000	0.136281000	1.984388000
H	-3.172070000	-0.763142000	2.466342000
O	0.019572000	-4.282139000	0.897328000
C	-4.825563000	0.733658000	2.151803000
H	-5.622497000	0.355486000	2.776177000
O	0.316014000	-1.900372000	-2.755401000
C	-4.880289000	1.925428000	1.334372000
H	-5.728806000	2.588589000	1.240558000
C	-3.619766000	2.071083000	0.661386000
H	-3.334773000	2.856760000	-0.022309000
C	-3.975851000	-0.915443000	-1.606034000
H	-2.966038000	-1.094292000	-1.948713000
C	-4.813724000	0.195345000	-1.985560000
H	-4.543868000	0.991022000	-2.665551000
C	-6.074247000	0.058554000	-1.293738000
H	-6.912622000	0.738130000	-1.360040000
C	-6.012494000	-1.136394000	-0.486550000
H	-6.796919000	-1.508708000	0.157699000
C	-4.714038000	-1.737501000	-0.677252000
H	-4.358363000	-2.645222000	-0.210096000
C	0.607289000	1.234567000	-0.459579000
C	1.667552000	0.676446000	0.402728000
H	1.628421000	0.663460000	1.486114000
C	2.907057000	0.631357000	-0.409165000
H	3.893376000	0.364485000	-0.056292000
C	2.578591000	1.121858000	-1.727833000
H	3.264009000	1.186490000	-2.559968000
C	1.195296000	1.514219000	-1.748420000
H	0.651197000	1.904760000	-2.595001000

C	1.508025000	4.402512000	0.610300000
H	0.485554000	4.554836000	0.926002000
C	2.036415000	4.669247000	-0.705070000
H	1.480120000	5.062015000	-1.544560000
C	3.431218000	4.295779000	-0.710661000
H	4.104103000	4.365906000	-1.553885000
C	3.765459000	3.800933000	0.603965000
H	4.733977000	3.437713000	0.918743000
C	2.577578000	3.867059000	1.418827000
H	2.497927000	3.557045000	2.451720000
C	3.111385000	-2.937466000	-0.577772000
C	2.619215000	-1.911557000	1.939668000
C	0.692941000	-3.339692000	0.590823000
C	0.839737000	-1.795801000	-1.695912000
N	-0.728056000	1.492433000	-0.133576000
H	-0.981657000	-0.252947000	1.006997000

TS(**W(CO)$_5$(*E*-2) → W(CO)$_4$(κ*C*, κ*N*-2)**)

Fe	-3.571560000	-1.105930000	-0.047590000
O	3.060593000	3.506087000	-0.407128000
O	-0.875673000	3.598559000	-2.296468000
Fe	2.987429000	-1.916702000	-0.158101000
W	0.365085000	1.855852000	0.101890000
C	-0.840539000	0.300725000	-0.565229000
C	-2.171607000	0.106403000	-1.071537000
C	-3.329951000	0.900156000	-0.669819000
H	-3.294288000	1.759766000	-0.016407000
O	-1.133613000	3.968470000	1.881919000
C	-4.486944000	0.360765000	-1.312692000
H	-5.496511000	0.735105000	-1.220010000
O	1.573041000	0.855544000	2.911877000
C	-4.080730000	-0.784752000	-2.098410000
H	-4.736675000	-1.409267000	-2.688429000
C	-2.668400000	-0.962203000	-1.944685000
H	-2.078602000	-1.742713000	-2.404373000
C	-2.747011000	-1.972798000	1.713599000
H	-1.736570000	-1.809854000	2.059228000
C	-3.171505000	-3.008923000	0.803718000
H	-2.540379000	-3.770748000	0.368474000
C	-4.590071000	-2.850921000	0.584925000
H	-5.207677000	-3.467023000	-0.053803000
C	-5.036759000	-1.714081000	1.353528000
H	-6.046581000	-1.329735000	1.390223000
C	-3.895138000	-1.168646000	2.049791000
H	-3.899576000	-0.309555000	2.705621000
C	0.909052000	-1.586701000	-0.615065000
C	1.062495000	-1.958333000	0.767342000
H	0.746977000	-1.390092000	1.626141000
C	1.680887000	-3.262973000	0.804436000
H	1.919691000	-3.816064000	1.701727000
C	1.926569000	-3.687648000	-0.549891000

H	2.384321000	-4.616759000	-0.857854000
C	1.464280000	-2.642266000	-1.431611000
H	1.493143000	-2.656973000	-2.513049000
C	4.165432000	-0.194118000	-0.567149000
H	3.806109000	0.743330000	-0.966255000
C	4.642922000	-1.315586000	-1.339200000
H	4.691702000	-1.369369000	-2.418083000
C	5.044095000	-2.353005000	-0.419704000
H	5.436672000	-3.323989000	-0.688709000
C	4.812073000	-1.872622000	0.921643000
H	5.003926000	-2.418590000	1.834975000
C	4.269902000	-0.539183000	0.830167000
H	3.989462000	0.086762000	1.664777000
C	2.039691000	2.937847000	-0.175499000
C	-0.406490000	2.914641000	-1.448967000
C	-0.525188000	3.183725000	1.211218000
C	1.140454000	1.151042000	1.847816000
N	0.203733000	-0.475839000	-1.188406000
H	0.069958000	-0.620544000	-2.191222000

TS(**W(CO)$_4$(*Z*-2) → W(CO)$_4$(κ*C*, κ*N*-2)**)

Fe	-3.164893000	-1.449252000	-0.129533000
O	2.581885000	2.990069000	1.896481000
O	-1.615144000	4.774077000	0.239200000
Fe	2.938370000	-1.992735000	-0.019420000
W	0.133958000	2.159268000	0.016403000
C	-0.666832000	0.530557000	-0.984672000
C	-1.840985000	-0.226169000	-1.301786000
C	-3.182919000	0.352992000	-1.251328000
H	-3.408795000	1.343767000	-0.884174000
O	-1.459865000	1.382456000	2.691154000
C	-4.097778000	-0.575566000	-1.834240000
H	-5.161205000	-0.427255000	-1.957157000
C	-3.357122000	-1.757665000	-2.224993000
H	-3.776044000	-2.639792000	-2.688638000
O	1.499527000	3.641211000	-2.500215000
C	-1.981973000	-1.567520000	-1.879742000
H	-1.181706000	-2.270710000	-2.049842000
C	-2.528577000	-1.748650000	1.884913000
H	-1.627538000	-1.342547000	2.318166000
C	-2.649514000	-3.014988000	1.207955000
H	-1.855013000	-3.730724000	1.051683000
C	-4.020943000	-3.154920000	0.777350000
H	-4.434102000	-3.991349000	0.230840000
C	-4.739467000	-1.971015000	1.184098000
H	-5.784008000	-1.766467000	0.993926000
C	-3.813953000	-1.097728000	1.865572000
H	-4.039272000	-0.130316000	2.290361000
C	1.009520000	-1.369627000	-0.602030000
C	0.975826000	-1.854720000	0.755567000
H	0.703706000	-1.272932000	1.623791000

C	1.390462000	-3.234294000	0.734478000
H	1.477230000	-3.881123000	1.595949000
C	1.693280000	-3.594381000	-0.630453000
H	2.039620000	-4.559960000	-0.971004000
C	1.462079000	-2.439975000	-1.462953000
H	1.584219000	-2.382929000	-2.535891000
C	4.300867000	-0.374942000	-0.182747000
H	4.063048000	0.622032000	-0.527413000
C	4.745351000	-1.477624000	-1.001823000
H	4.892760000	-1.456230000	-2.072800000
C	4.960612000	-2.614809000	-0.139405000
H	5.288936000	-3.596474000	-0.451883000
C	4.648751000	-2.214274000	1.211978000
H	4.701394000	-2.843752000	2.089415000
C	4.241594000	-0.829962000	1.185279000
H	3.944693000	-0.235586000	2.037929000
C	1.637064000	2.755279000	1.208062000
C	-0.922604000	3.799799000	0.175184000
C	-0.852746000	1.614194000	1.695927000
C	1.020829000	3.047344000	-1.591663000
N	0.659216000	-0.040189000	-1.019515000
H	1.298101000	0.358937000	-1.701786000

TS(**W(CO)$_5$(*E*-2) → [W(CO)$_4$···(*E*-3)]**)

Fe	3.517871000	-1.435081000	0.102501000
O	-1.206174000	4.509894000	-0.621579000
O	-0.645678000	2.230151000	3.220658000
Fe	-3.704177000	-1.178103000	-0.272936000
W	-0.031817000	1.644754000	0.120121000
C	0.395086000	-0.575741000	0.389097000
C	1.582927000	-1.229993000	0.960262000
C	2.580700000	-0.596236000	1.805950000
H	2.599125000	0.445253000	2.085617000
O	2.813241000	2.855936000	0.656394000
C	3.515876000	-1.599232000	2.223580000
H	4.373441000	-1.436957000	2.860999000
O	0.829376000	1.353229000	-2.966527000
C	3.123265000	-2.858906000	1.631570000
H	3.637923000	-3.802009000	1.751800000
C	1.944677000	-2.637591000	0.845667000
H	1.391772000	-3.376878000	0.286429000
C	3.653171000	-0.611372000	-1.860880000
H	2.843964000	-0.155140000	-2.411497000
C	4.007625000	-2.008121000	-1.885075000
H	3.510939000	-2.780518000	-2.455413000
C	5.145707000	-2.192288000	-1.014916000
H	5.645363000	-3.129075000	-0.810127000
C	5.489565000	-0.907455000	-0.454104000
H	6.288780000	-0.714740000	0.248077000
C	4.563418000	0.070179000	-0.972478000
H	4.547221000	1.123240000	-0.729753000

C	-1.672098000	-0.977476000	-0.776106000
C	-2.266700000	0.347511000	-0.605298000
H	-2.167590000	1.019376000	0.262218000
C	-3.325743000	0.474671000	-1.571923000
H	-3.972926000	1.332211000	-1.686923000
C	-3.381521000	-0.742020000	-2.341922000
H	-4.066254000	-0.944520000	-3.152766000
C	-2.384176000	-1.649591000	-1.843030000
H	-2.140341000	-2.628727000	-2.226444000
C	-3.919698000	-2.366916000	1.470127000
H	-3.106212000	-2.835033000	2.007030000
C	-4.658664000	-2.951797000	0.376951000
H	-4.496532000	-3.932961000	-0.046600000
C	-5.646357000	-1.992036000	-0.055368000
H	-6.349923000	-2.126237000	-0.865288000
C	-5.515328000	-0.813385000	0.766873000
H	-6.108996000	0.086459000	0.687502000
C	-4.448569000	-1.046312000	1.711316000
H	-4.108495000	-0.352370000	2.467654000
C	-0.743680000	3.446632000	-0.337033000
C	-0.450296000	1.979445000	2.074989000
C	1.723398000	2.397705000	0.455708000
C	0.492581000	1.433678000	-1.832776000
N	-0.527448000	-1.516269000	-0.161482000
H	-0.587845000	-0.900293000	1.121571000

TS(**W(CO)$_4$(*Z*-2) → W(CO)$_4$(*Z*-3)**)

Fe	3.405244000	-1.502222000	0.206081000
O	-2.437633000	4.018910000	-0.148026000
O	2.105565000	4.163699000	0.649865000
Fe	-3.358401000	-1.689509000	-0.167494000
W	-0.018996000	1.962888000	-0.061528000
C	0.593108000	-0.165617000	0.052234000
C	1.440603000	-1.036936000	0.842627000
C	2.423114000	-0.511921000	1.784605000
H	2.539936000	0.530987000	2.039977000
O	0.527636000	2.721661000	-3.140858000
C	3.157851000	-1.614422000	2.324863000
H	3.945313000	-1.550285000	3.061912000
C	2.673373000	-2.828640000	1.706998000
H	3.045695000	-3.825807000	1.895043000
O	-0.530268000	1.827028000	3.108304000
C	1.627915000	-2.485172000	0.788698000
H	1.078919000	-3.177214000	0.169459000
C	3.820903000	-0.697597000	-1.720446000
H	3.131358000	-0.118647000	-2.319620000
C	3.969965000	-2.132139000	-1.742197000
H	3.417361000	-2.820996000	-2.365638000
C	4.994718000	-2.484543000	-0.787326000
H	5.335711000	-3.485186000	-0.560803000
C	5.473969000	-1.267795000	-0.177380000

H	6.234971000	-1.198228000	0.587356000
C	4.745626000	-0.161839000	-0.751767000
H	4.870220000	0.882421000	-0.502361000
C	-1.333404000	-1.638334000	-0.783153000
C	-2.202538000	-1.885521000	-1.920293000
H	-2.270706000	-1.258164000	-2.797223000
C	-2.899272000	-3.116940000	-1.679166000
H	-3.611818000	-3.582081000	-2.345161000
C	-2.498968000	-3.618174000	-0.384362000
H	-2.868807000	-4.516893000	0.088799000
C	-1.538842000	-2.709161000	0.181191000
H	-1.066196000	-2.802718000	1.146564000
C	-3.905868000	0.129716000	0.785673000
H	-3.242861000	0.965381000	0.959069000
C	-4.125393000	-0.979318000	1.680771000
H	-3.661148000	-1.117216000	2.647296000
C	-5.076525000	-1.870540000	1.059474000
H	-5.443962000	-2.798411000	1.475490000
C	-5.442152000	-1.310919000	-0.219707000
H	-6.128989000	-1.748415000	-0.931016000
C	-4.715305000	-0.075441000	-0.390718000
H	-4.768480000	0.582148000	-1.246841000
C	-1.499451000	3.281600000	-0.107674000
C	1.269693000	3.347807000	0.377119000
C	0.325799000	2.377561000	-2.021216000
C	-0.355093000	1.824962000	1.934015000
N	-0.536066000	-0.480812000	-0.705066000
H	0.538363000	0.061855000	-1.268466000

TS(**W(CO)$_4$(*Z*-2) → *cis*(N,H)-W(CO)$_4$(H)(*Z*-15)**)

Fe	3.472870000	-1.453508000	0.241152000
O	-2.154723000	3.877688000	-1.452203000
O	1.614657000	4.490454000	0.877229000
Fe	-3.351093000	-1.712272000	-0.207353000
W	-0.073452000	1.941092000	-0.006465000
C	0.619010000	-0.077998000	0.526487000
C	1.585892000	-0.979744000	1.096933000
C	2.668643000	-0.500604000	1.947836000
H	2.815928000	0.532551000	2.227615000
O	1.460746000	1.881170000	-2.837407000
C	3.441817000	-1.628148000	2.367468000
H	4.304231000	-1.599713000	3.017954000
C	2.876174000	-2.814961000	1.763311000
H	3.252332000	-3.821842000	1.878395000
O	-1.415204000	2.262249000	2.898504000
C	1.744846000	-2.428246000	0.972898000
H	1.122869000	-3.090492000	0.391363000
C	3.684789000	-0.718035000	-1.747890000
H	2.921194000	-0.204707000	-2.312978000
C	3.906148000	-2.141502000	-1.722366000
H	3.338525000	-2.882895000	-2.267084000

C	5.021615000	-2.402171000	-0.842740000
H	5.430513000	-3.374484000	-0.605215000
C	5.486520000	-1.137301000	-0.325953000
H	6.302094000	-0.997582000	0.370001000
C	4.656896000	-0.095435000	-0.881357000
H	4.749368000	0.963693000	-0.686937000
C	-1.285311000	-1.392105000	-0.550441000
C	-1.997455000	-1.405697000	-1.807611000
H	-2.023165000	-0.598755000	-2.525763000
C	-2.618711000	-2.697682000	-1.936607000
H	-3.218592000	-3.027693000	-2.772604000
C	-2.308069000	-3.470800000	-0.758218000
H	-2.641263000	-4.478320000	-0.553204000
C	-1.494161000	-2.662184000	0.114614000
H	-1.112425000	-2.944523000	1.084106000
C	-4.142754000	-0.221775000	1.085647000
H	-3.586029000	0.582970000	1.544359000
C	-4.357582000	-1.531904000	1.650218000
H	-3.981085000	-1.882692000	2.600991000
C	-5.162063000	-2.290678000	0.722318000
H	-5.488049000	-3.313375000	0.852073000
C	-5.443465000	-1.448827000	-0.416104000
H	-6.017035000	-1.730113000	-1.288407000
C	-4.811961000	-0.170836000	-0.192562000
H	-4.839156000	0.677597000	-0.862165000
C	-1.342198000	3.205607000	-0.898519000
C	0.978005000	3.546372000	0.532845000
C	0.895593000	1.856631000	-1.794951000
C	-0.932650000	2.110338000	1.826204000
N	-0.517061000	-0.299370000	-0.090006000
H	-1.502795000	0.849040000	-0.140578000

TS(***cis*(N,H)-W(CO)$_4$(H)(*Z*-15) → *cis*(C,H)-W(CO)$_4$(H)(*Z*-15)**)

Fe	3.432344000	-1.589899000	0.099552000
O	1.845212000	4.436577000	-0.783457000
O	-2.396161000	2.485490000	1.866776000
Fe	-3.423386000	-1.508024000	-0.201042000
W	0.107972000	1.859762000	-0.070116000
C	0.570859000	-0.230681000	0.192782000
C	1.444325000	-1.236185000	0.743153000
C	2.384514000	-1.011657000	1.833317000
H	2.513812000	-0.084927000	2.370470000
O	1.519405000	2.349753000	2.752038000
C	3.065738000	-2.244683000	2.094847000
H	3.812924000	-2.403106000	2.859158000
C	2.591034000	-3.235215000	1.156064000
H	2.931525000	-4.258826000	1.088484000
O	-1.890687000	3.227229000	-2.243733000
C	1.601393000	-2.624110000	0.316851000
H	1.084223000	-3.098717000	-0.502916000
C	3.925453000	-0.338912000	-1.544411000

H	3.233841000	0.366970000	-1.982219000
C	4.077697000	-1.723882000	-1.916149000
H	3.545154000	-2.231092000	-2.708538000
C	5.062260000	-2.314701000	-1.039474000
H	5.394491000	-3.343483000	-1.056736000
C	5.515595000	-1.292921000	-0.126250000
H	6.244893000	-1.423602000	0.661278000
C	4.810109000	-0.071833000	-0.436435000
H	4.920333000	0.874930000	0.073208000
C	-1.491900000	-1.071008000	-1.005641000
C	-2.525066000	-0.639930000	-1.921494000
H	-2.665919000	0.368326000	-2.281211000
C	-3.292371000	-1.794628000	-2.298910000
H	-4.131124000	-1.800141000	-2.980177000
C	-2.755451000	-2.939674000	-1.603681000
H	-3.125303000	-3.953266000	-1.668170000
C	-1.654062000	-2.499085000	-0.787474000
H	-1.061961000	-3.118805000	-0.133010000
C	-3.659627000	-0.699187000	1.750931000
H	-2.895209000	-0.188647000	2.317540000
C	-3.923386000	-2.116708000	1.766843000
H	-3.386302000	-2.856428000	2.344126000
C	-5.028106000	-2.374822000	0.873841000
H	-5.460249000	-3.342788000	0.660462000
C	-5.445001000	-1.114998000	0.305387000
H	-6.245144000	-0.974347000	-0.408116000
C	-4.597858000	-0.079552000	0.845904000
H	-4.657950000	0.975770000	0.621574000
C	1.171924000	3.512386000	-0.476507000
C	-1.475019000	2.195897000	1.174678000
C	1.017799000	2.141329000	1.694591000
C	-1.152089000	2.720777000	-1.483585000
N	-0.512173000	-0.219109000	-0.493617000
H	1.255622000	1.461056000	-1.380663000

TS(***cis*(C,H)-W(CO)$_4$(H)(*Z*-15) → W(CO)$_4$(*Z*-3)**)

Fe	3.578710000	-1.329485000	0.242575000
O	1.152152000	4.697161000	0.718606000
O	-2.785354000	3.310044000	-1.121346000
Fe	-3.262121000	-1.750424000	-0.209769000
W	-0.138278000	1.887442000	-0.001646000
C	0.666858000	-0.134570000	0.426044000
C	1.663180000	-0.962374000	1.058164000
C	2.695429000	-0.462590000	1.954191000
H	2.799333000	0.564531000	2.273501000
O	-1.357986000	1.992853000	2.973947000
C	3.504128000	-1.572190000	2.361375000
H	4.347118000	-1.526295000	3.035707000
C	3.003539000	-2.762017000	1.711314000
H	3.415315000	-3.756584000	1.808203000
O	1.148532000	2.143997000	-2.942704000

C	1.877078000	-2.397200000	0.901794000
H	1.296315000	-3.062774000	0.282419000
C	3.776619000	-0.467775000	-1.693109000
H	2.999898000	0.063532000	-2.224322000
C	4.027115000	-1.886076000	-1.756443000
H	3.472617000	-2.603387000	-2.345159000
C	5.148105000	-2.178505000	-0.894106000
H	5.577599000	-3.155051000	-0.718615000
C	5.587866000	-0.939367000	-0.299066000
H	6.402581000	-0.826215000	0.402660000
C	4.737476000	0.118556000	-0.789776000
H	4.808387000	1.165238000	-0.529537000
C	-1.214193000	-1.395943000	-0.652844000
C	-2.008217000	-1.353201000	-1.862371000
H	-2.077408000	-0.508693000	-2.532506000
C	-2.624828000	-2.642040000	-2.024897000
H	-3.271068000	-2.934906000	-2.839993000
C	-2.243262000	-3.471030000	-0.905678000
H	-2.563019000	-4.488759000	-0.731489000
C	-1.378460000	-2.704326000	-0.046414000
H	-0.944753000	-3.035797000	0.885236000
C	-3.996099000	-0.324526000	1.183447000
H	-3.411860000	0.460639000	1.639867000
C	-4.174905000	-1.657562000	1.704379000
H	-3.750388000	-2.042749000	2.621051000
C	-5.020332000	-2.384700000	0.787267000
H	-5.334663000	-3.413985000	0.891636000
C	-5.362704000	-1.499181000	-0.300173000
H	-5.976444000	-1.749916000	-1.154334000
C	-4.727518000	-0.226193000	-0.056483000
H	-4.788097000	0.646834000	-0.691106000
C	0.639823000	3.658921000	0.437557000
C	-1.802326000	2.802517000	-0.695786000
C	-0.912551000	1.901111000	1.877204000
C	0.668451000	2.004606000	-1.867346000
N	-0.422182000	-0.323037000	-0.223862000
H	1.514678000	1.334041000	0.385149000
